# Supplementary material for: A sex-specific switch between visual and olfactory inputs underlies adaptive sex differences in behavior
Source: Curr Biol. 2021 Mar 22;31(6):1175–1191.e6. doi: 10.1016/j.cub.2020.12.047 (PMC7987718; doi:10.1016/j.cub.2020.12.047)
Supplement: Document S1. Figures S1–S6 and Tables S1 and S3 [file mmc1.pdf]

Current Biology, Volume *31*

Supplemental Information

A sex-specific switch between visual and olfactory inputs underlies adaptive sex differences in behavior

Tetsuya Nojima, Annika Rings, Aaron M. Allen, Nils Otto, Thomas A. Verschut, Jean-Christophe Billeter, Megan C. Neville, and Stephen F. Goodwin

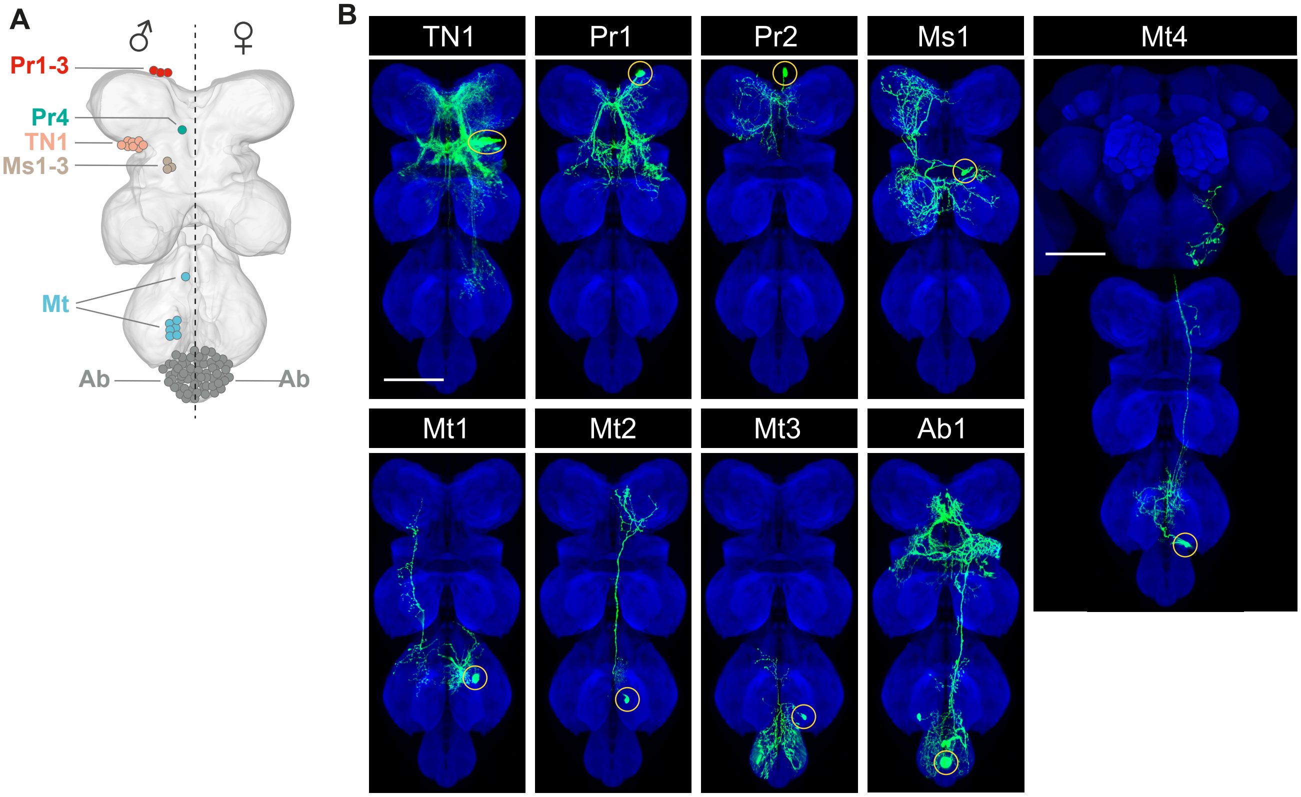
A
♂
♀
Pr1-3
Pr4
TN1
Ms1-3
Mt
Ab
Ab
B
TN1
Pr1
Pr2
Ms1
Mt4
Mt1
Mt2
Mt3
Ab1

Figure S1: Comprehensive single cluster-level mapping of *dsx*-expressing neurons in the male VNC. Related to Figure 1.

(**A**) A schematic drawing representing *dsx* expression pattern in the VNC. Male neurons are shown in the thoracic neuromeres on the left, the absence of female neurons is shown on the right. Neuronal differences between the sexes are not distinguished in the abdominal neuromere. (**B**) Male single neuroblast clones generated by dsx^{Gal4}-driven MARCM. Cell body positions are indicated by yellow circles. Scale bars, 50 μm.

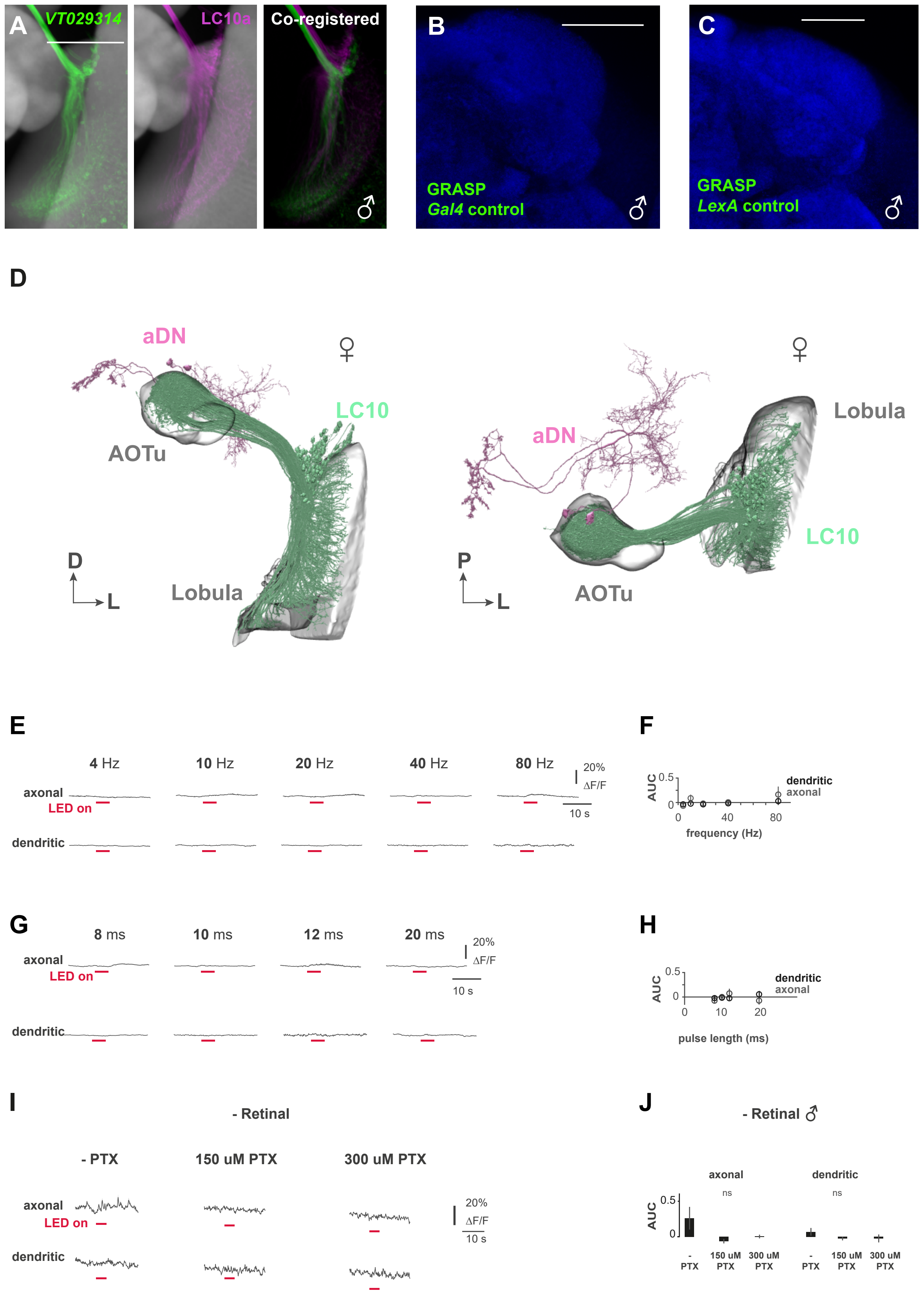
A
VT029314
LC10a
Co-registered
B
GRASP
Gal4 control
C
GRASP
LexA control
D
aDN
LC10
AOTu
Lobula
D
L
aDN
Lobula
LC10
AOTu
P
L
E
4 Hz
10 Hz
20 Hz
40 Hz
80 Hz
20%
ΔF/F
10 s
axonal
LED on
dendritic
F
AUC
0.5
0
0
40
80
frequency (Hz)
dendritic
axonal
G
8 ms
10 ms
12 ms
20 ms
20%
ΔF/F
10 s
axonal
LED on
dendritic
H
AUC
0.5
0
0
10
20
pulse length (ms)
dendritic
axonal
I
- Retinal
- PTX
150 uM PTX
300 uM PTX
axonal
LED on
dendritic
20%
ΔF/F
10 s
J
- Retinal
axonal
dendritic
ns
ns
AUC
0.5
0
- PTX
150 uM PTX
300 uM PTX
- PTX
150 uM PTX
300 uM PTX

Figure S2: Connectivity between LC10a and aDN is male-specific. Related to Figure 3.

(**A**) LC10 neurons labeled by *VT029314-LexA* (green) and *OL0019B* split-*Gal4* (magenta) are co-registered (right). Scale bar, 50 µm. (**B and C**) Genetic controls for the GRASP experiment either without *LexA* (**B**) or *Gal4* (**C**) driver. Scale bars, 30 µm. (**D**) Positions of LC10 (green) axon terminals and aDN (magenta) dendritic fields in females are analyzed using the EM-level connectomics database neuPrint. Anterior (left) and dorsal (right) views are shown. Two neuropil regions, lobula and AOTu, which are innervated by LC10 dendrites and axons, respectively, are indicated in gray. (**E**) Mean (dark gray line) and standard error (SE; light gray shaded area) of ΔF/F in aDN axonal (top) and dendritic (bottom) compartments in response to a 5 s (40 Hz, 10 ms pulses) optogenetic stimulation of LC10a neurons (pink bar) in control males not fed on all-*trans*-retinal; Left: without picrotoxin (- PTX) and CGP54626 (n = 13 male); Middle: 150 µM picrotoxin and 50 µM CGP54626 (n = 8 male flies); Right: 300 µM picrotoxin and 50 µM CGP54626 (n = 10 and 8 male flies in axonal and dendritic compartments, respectively); (**F**) Mean and SE of the area under the ΔF/F curve (AUC) from beginning until 1s after the end of the stimulus for experiments in (**E**). (**G**) Mean and SE of ΔF/F traces in aDN axonal (top traces) and dendritic (bottom traces) compartments in response to different frequencies (4-80 Hz, 10 ms pulses) of a 5 s optogenetic activation of LC10a neurons in 9-28 males for each frequency. (**H**) AUC vs. frequency for data in (**G**) in axonal (light gray circles) and dendritic (dark gray circles) compartments. (**I**) Mean ΔF/F traces in aDN axonal (top traces) and dendritic (bottom traces) compartments in response to different pulse lengths (8-20 ms, 40 Hz pulses) of a 5 s optogenetic activation of LC10a neurons in 5-28 males for each pulse length. (**J**) AUC vs. pulse length for data in (**I**). ns – *p.adj*>0.05 by Mann-Whitney-U test adjusted for multiple comparisons with the Holm method.

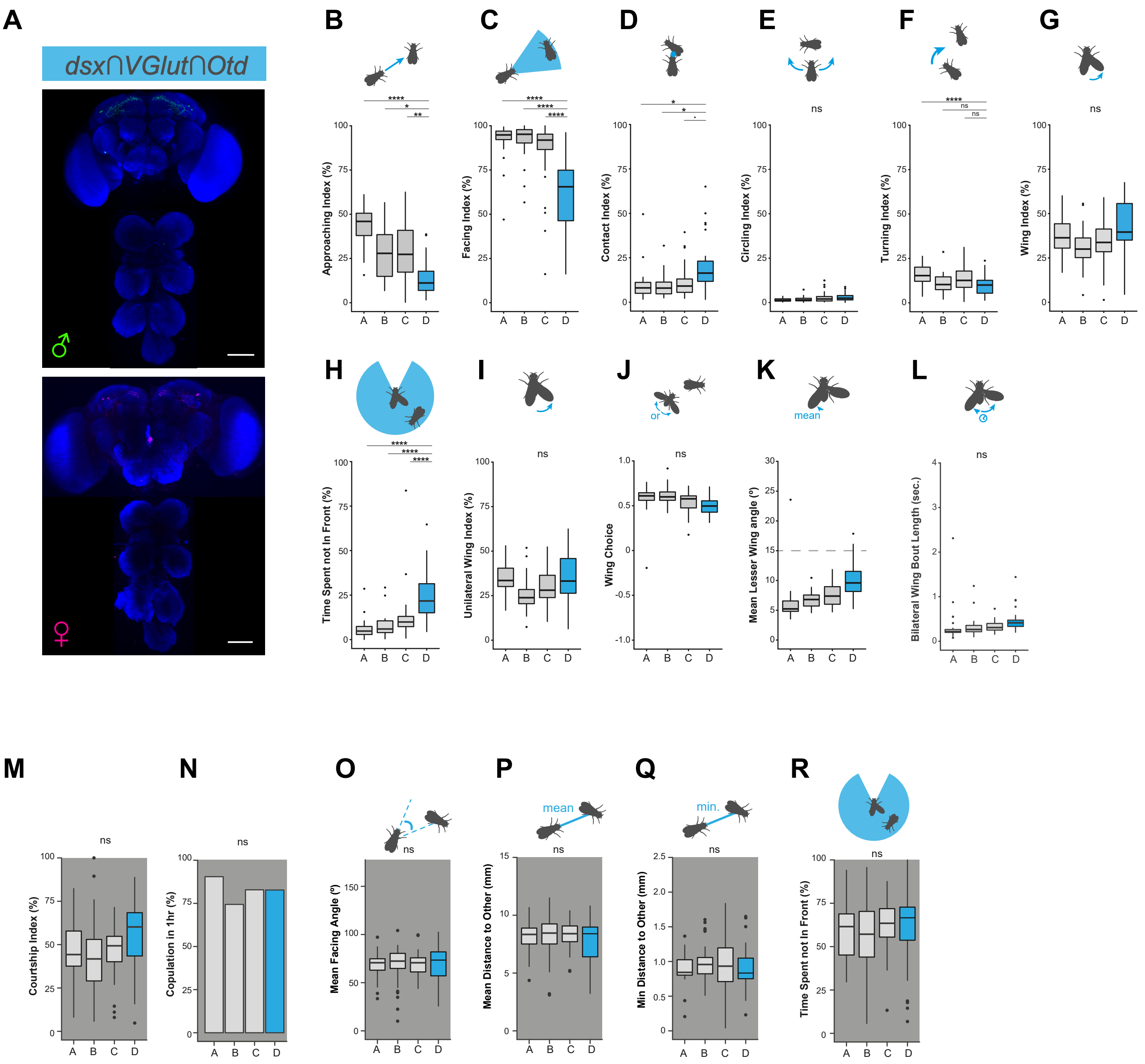
A
dsx∩VGlut∩Otd
B
Approaching Index (%)
C
Facing Index (%)
D
Contact Index (%)
E
Circling Index (%)
F
Turning Index (%)
G
Wing Index (%)
H
Time Spent not In Front (%)
I
Unilateral Wing Index (%)
J
or
Wing Choice
K
mean
Mean Lesser Wing angle (º)
L
Bilateral Wing Bout Length (sec.)
M
Courtship Index (%)
N
Copulation in 1hr (%)
O
Mean Facing Angle (º)
P
mean
Mean Distance to Other (mm)
Q
min.
Min Distance to Other (mm)
R
Time Spent not In Front (%)
ns
A B C D

Figure S3: Silencing male aDN alters visually guided courtship behavior. Related to Figure 4.

(**A**) Spatially restricted expression in aDN cluster. *dsx∩VGlut∩Otd* expression patterns shown using *dsx*$^{Gal4.DBD}$*/VGlut*$^{dVP16.AD}$*/Otd-FLP > UAS>stop>mCD8::GFP* in the male (green) and female (magenta) brains and VNCs. Scale bar, 50 μm. (**B-L**) Schematic representations of the behaviors are inset. (**B-F**) Individual indices of each component of courtship behavior for which a JAABA classifier was trained. The reported *p.adj* are *p* values adjusted (Holm method) for comparison of multiple behaviors. (**B**) Approaching index. ($F_{(3,114)}$ = 26.2, *p.adj <0.0001*). (**C**) Facing index ($F_{(3,114)}$ = 23.7, *p.adj <0.0001*). (**D**) Contact index ($F_{(3,114)}$ = 6.91, *p.adj* = 0.008). (**E**) Circling index ($F_{(3,114)}$ = 4.11, *p.adj* = 0.16). (**F**) Turning index ($F_{(3,114)}$ = 6.61, *p.adj* = 0.011). (**G**) Wing extension index. ($F_{(3,114)}$ = 3.87, *p* = 0.012, *p.adj* = 0.19). (**H**) Percent of time when the female was not in front of the male while the males wing was extended. ($F_{(3,114)}$ = 28.6, *p.adj <0.0001*). (**I**) Unilateral wing index. ($F_{(3,114)}$ = 3.40, *p.adj* = 0.32). (**J**) Wing choice index. ($F_{(3,114)}$ = 4.41, *p.adj* = 0.11). (**K**) Bilateral wing bout length. ($F_{(3,114)}$ = 0.619, *p.adj* = 1.0). (**L**) Mean lesser wing angle. (**M**) Courtship Index (%) in the dark. ($F_{(1,123)}$ = 3.143, *p.adj* = 0.17). (**N**) Copulation (%) over a 60-minute time period in the dark. (Fisher's exact test, *p.adj* = 1.00). (**O**) Mean facing angle in the dark. ($F_{(1,123)}$ = 0.173, *p.adj* = 1.00). (**P**) Mean distance to other in the dark. ($F_{(1,123)}$ = 0.84, *p.adj* = 1.00). (**Q**) Minimum distance to other in the dark. ($F_{(1,123)}$ = 0.453, *p.adj* = 1.00). (**R**) Percentage of time when the female was not in front of the male while the male's wing was extended in the dark. ($F_{(1,123)}$ = 0.536, *p.adj* = 1.00) Full genotypes: A=*Otd-FLP/+; dsx*$^{Gal4.DBD}$*/+* (n_{light}=31, n_{dark}=21), B=*VGlut*$^{dVP16.AD}$*, UAS>stop>TNT/+* (n_{light} =20, n_{dark}=43), C=*VGlut*$^{dVP16.AD}$*, UAS>stop>TNT/+; dsx*$^{Gal4.DBD}$*/+* (n_{light}=34, n_{dark}=35), D=*VGlut*$^{dVP16.AD}$*, UAS>stop>TNT/Otd-FLP; dsx*$^{Gal4.DBD}$*/+* (n_{light} =33, n_{dark}=29). ˙*p.adj*<0.1, **p.adj*<0.05, ***p.adj*<0.01, ****p.adj*<0.001, *****p.adj*<0.0001 by t-test (**B-R**) and adjusted with the Holm method. ns – *p.adj*>0.1.

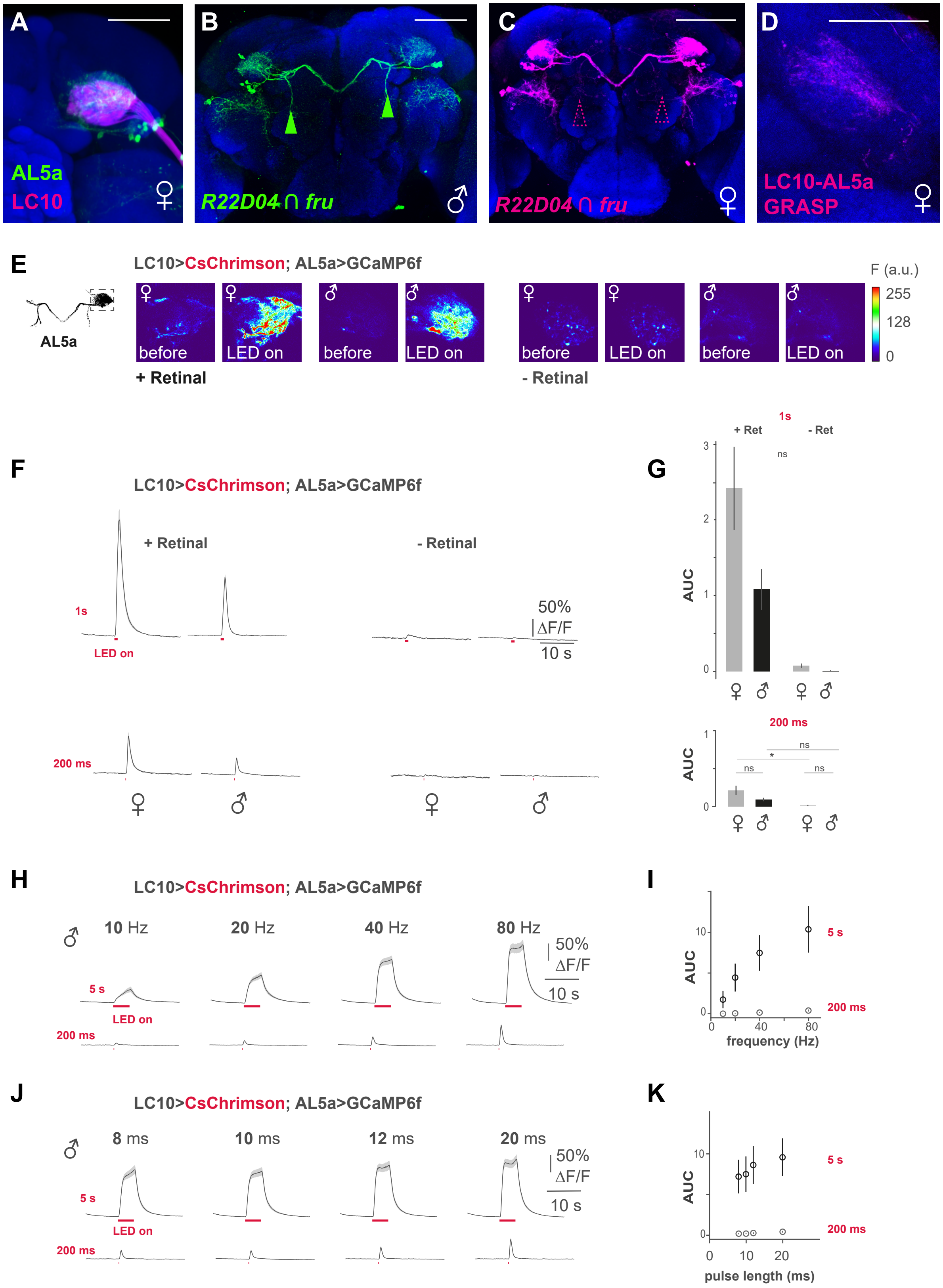

A
AL5a
LC10
B
R22D04∩fru
C
R22D04∩fru
D
LC10-AL5a GRASP
E
AL5a
LC10>CsChrimson; AL5a>GCaMP6f
before
LED on
+ Retinal
- Retinal
F (a.u.)
255
128
0
F
LC10>CsChrimson; AL5a>GCaMP6f
+ Retinal
- Retinal
1s
LED on
200 ms
50% ΔF/F
10 s
G
1s
+ Ret
- Ret
ns
AUC
200 ms
*
H
LC10>CsChrimson; AL5a>GCaMP6f
10 Hz
20 Hz
40 Hz
80 Hz
5 s
LED on
200 ms
50% ΔF/F
10 s
I
AUC
frequency (Hz)
5 s
200 ms
J
LC10>CsChrimson; AL5a>GCaMP6f
8 ms
10 ms
12 ms
20 ms
5 s
LED on
200 ms
50% ΔF/F
10 s
K
AUC
pulse length (ms)
5 s
200 ms

Figure S4: *fru*-expressing AL5a is an additional downstream cluster of LC10a. Related to Figure 5.

(**A**) The female LC10a (magenta) and AL5a (green) co-registered on to the template brain. Scale bar, 30 µm. (**B,C**) Expression patterns of *GMR22D04-Gal4*∩*fru*^{FLP} in the male (green) and female (magenta) brains. Solid filled (**B**) and dashed open (**C**) arrowheads indicate the presence and absence, respectively, of male-specific longitudinal neurite branches. Scale bars, 50 µm. (**D**) GRASP signals between LC10a and AL5a in the female (magenta). Scale bar, 30 µm. (**E**) Left: Maximum projection of a confocal stack of mCD8::GFP-labeled AL5a in a male. Recording region in the AOTu for calcium imaging is indicated by a gray box. Right: Example fluorescence recordings from the input sites of AL5a neurons in the AOTu expressing GCaMP6f in males and females before (left panel) and during (right panel) a 5 s (40 Hz, 10 ms) optogenetic stimulation of LC10a neurons expressing CsChrimson. Left (+ Retinal) Right (-Retinal). (**F**) Mean (dark gray) and SE (light gray shaded area) of ΔF/F traces in AL5a neuron input sites in response to a 1 s (top) and 200 ms (bottom) optogenetic activation (pink bar, 40 Hz, 10 ms pulses) of LC10a neurons; Averages of 4 pulses each in retinal fed (+Retinal, left) female (left, n=7) and male (right, n=11) flies and in non-retinal control (-Retinal, right) female (left, n=8) and male (right, n=7) flies. (**G**) Mean and SE of AUC for experiments in (**F**); Top: 1 s optogenetic stimulation; Bottom: 200 ms optogenetic stimulation. (**H**) Mean ΔF/F traces in AL5a neurons in response to different frequencies (10 - 80 Hz, 10 ms pulses) of a 5 s (top) and 200 ms (bottom) optogenetic activation of LC10a in n = 11-13 males for each frequency. (**I**) Mean and SE of AUC for experiments in (**H**) for 5 s (dark gray circles) and 200 ms (light gray circles) optogenetic stimulation of LC10a. (**J**) Mean ΔF/F traces in AL5a neurons in response to different pulse lengths (8 - 20 ms, 40 Hz pulses) of a 5 s (top) and 200 ms (bottom) optogenetic activation of LC10a in 10 -13 males for each pulse length. (**K**) Mean and SE of AUC for experiments in (J) for 5 s (dark gray circles) and 200 ms (light gray circles) optogenetic stimulation of LC10a. ns – *p.adj*>0.05, **p.adj*<0.05, ***p.adj*<0.01, ****p.adj*<0.001, *****p.adj*<0.0001 by Mann-Whitney-U test adjusted for multiple comparisons with the Holm method.

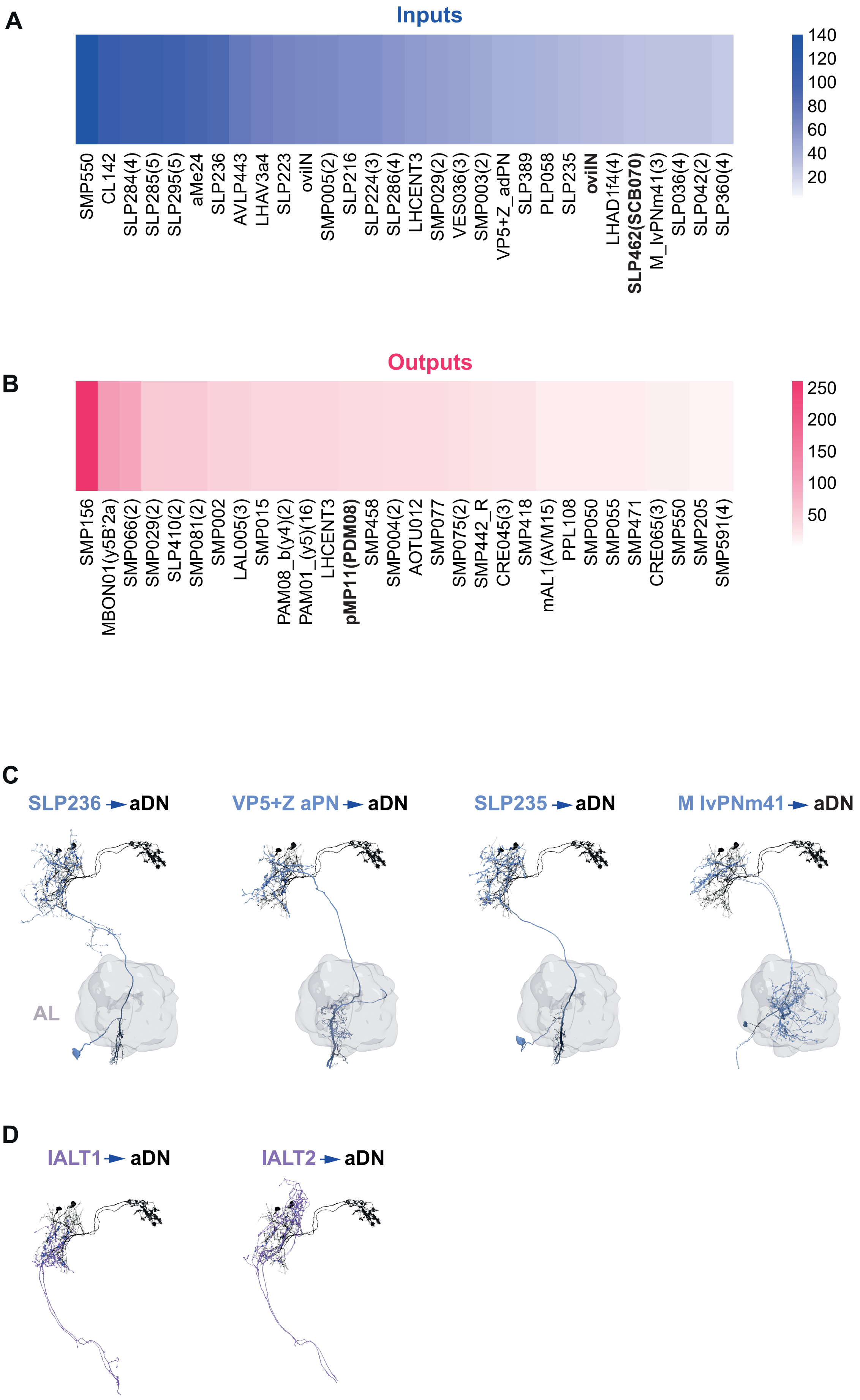
A
Inputs
140
120
100
80
60
40
20
SMP550
CL142
SLP284(4)
SLP285(5)
SLP295(5)
aMe24
SLP236
AVLP443
LHAV3a4
SLP223
oviIN
SMP005(2)
SLP216
SLP224(3)
SLP286(4)
LHCENT3
SMP029(2)
VES036(3)
SMP003(2)
VP5+Z_adPN
SLP389
PLP058
SLP235
oviIN
LHAD1f4(4)
SLP462(SCB070)
M_lvPNm41(3)
SLP036(4)
SLP042(2)
SLP360(4)
B
Outputs
250
200
150
100
50
SMP156
MBON01(y5B'2a)
SMP066(2)
SMP029(2)
SLP410(2)
SMP081(2)
SMP002
LAL005(3)
SMP015
PAM08_b(y4)(2)
PAM01_(y5)(16)
LHCENT3
pMP11(PDM08)
SMP458
SMP004(2)
AOTU012
SMP077
SMP075(2)
SMP442_R
CRE045(3)
SMP418
mAL1(AVM15)
PPL108
SMP050
SMP055
SMP471
CRE065(3)
SMP550
SMP205
SMP591(4)
C
SLP236 aDN
VP5+Z aPN aDN
SLP235 aDN
M lvPNm41 aDN
AL
D
lALT1 aDN
lALT2 aDN

Figure S5: Female aDN connectivity. Related to Figure 6.

(**A and B**) Heatmaps representing number of synaptic connections to both aDN combined input (**A**) and output (**B**) by neuronal types. Neurons labeled in bold are contralateral, all others are ipsilateral. Color scales represent the number of synapses. (**C and D**) 3D reconstructions of volumetric EM data of aDN (2 neurons, grey) and projection neurons in the right-brain hemisphere. (**C**) The multiglomerular multimodal projection neurons VP5+Z aPN (bodyid: 5813063239) and M lvPNm41 (bodyids: 730252777, 1765415970, 1795410644) are shown as well as the uncharacterized non-olfactory projection neurons SLP236 (bodyid: 420274150) and SLP235 (5813056890). Inputs into the aDN are shown in blue. The antennal lobe (AL) is shown. (**D**) The lALT-tract neurons classes lALT1 (bodyid: 888500672) and lALT2 (bodyid: 5901195362) are shown.

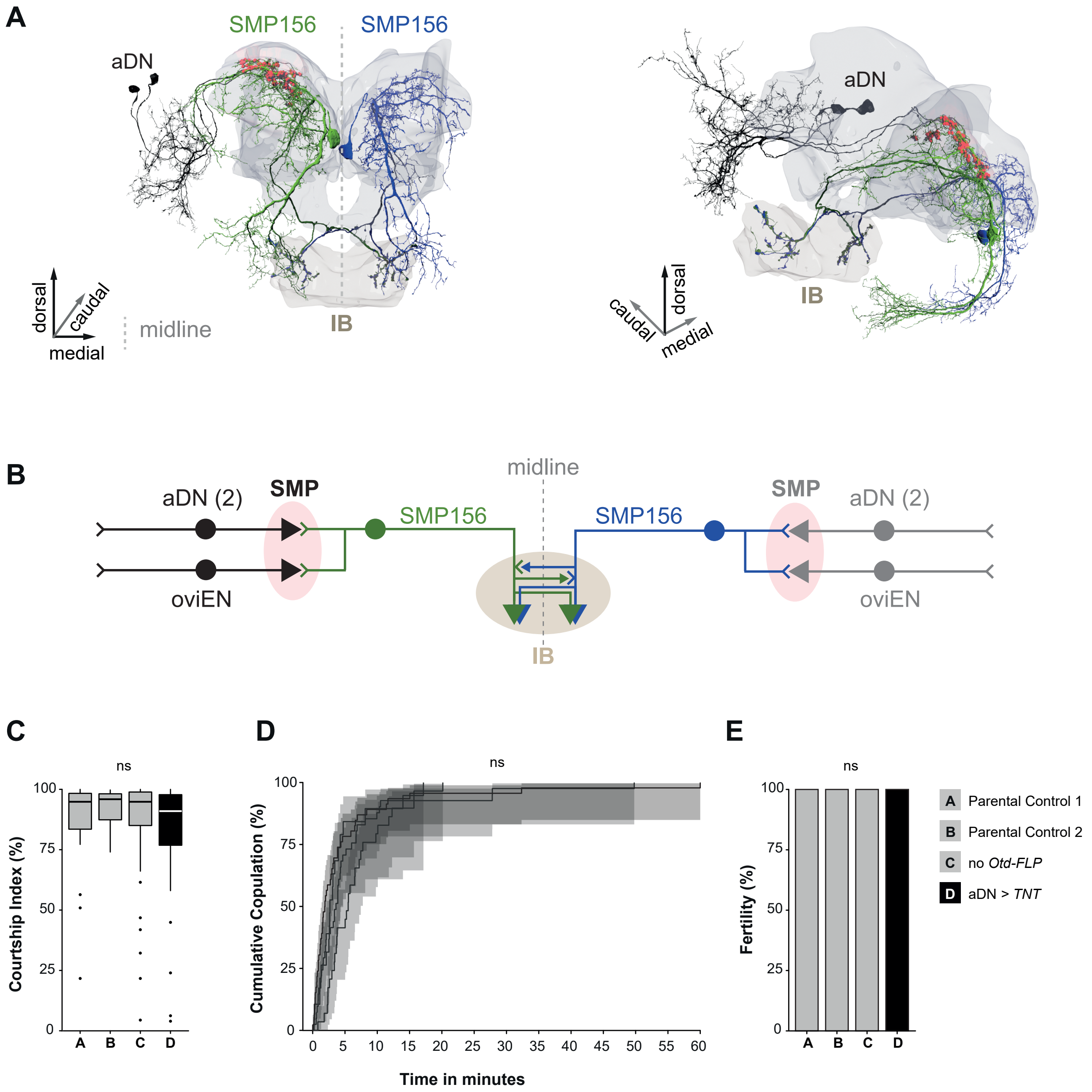
A
SMP156
SMP156
aDN
dorsal
caudal
medial
midline
IB
aDN
IB
caudal
dorsal
medial
B
midline
aDN (2)
SMP
SMP156
SMP156
SMP
aDN (2)
oviEN
oviEN
IB
C
ns
Courtship Index (%)
100
75
50
25
0
A
B
C
D
D
ns
Cumulative Copulation (%)
100
75
50
25
0
0
5
10
15
20
25
30
35
40
45
50
55
60
Time in minutes
E
ns
Fertility (%)
100
75
50
25
0
A
B
C
D
A Parental Control 1
B Parental Control 2
C no Otd-FLP
D aDN > TNT

Figure S6: Female aDNs are not involved in virgin female behavior. Related to Figure 7.

(**A**) 3D reconstructions of volumetric EM data from aDN (2 neurons, grey) and the contra- and ipsilateral downstream neurons SMP156 (bodyids: 5813022337, 673776769). The inferior bridge (IB) and the SMP for both hemispheres are shown as well. Synapses from aDN to SMP156 are shown in red. (**B**) Circuit diagram shows aDN and oviEN inputs to SMP156 that projects to the IB. (**C-E**) Silenced female aDN behaviors (ns – *p.adj*>0.05). (**C**) Control male courtship indices toward female. ($F_{(3,131)} = 1.1$, $p = 0.49$, $p.adj = 1$). (**D**) Cumulative proportional copulation (% ± 95% confidence intervals) over a 60-minute time period. ($\chi^2_{(3)} = 5.5$, $p = 0.14$, $p.adj = 1$). (**E**) Individual female fertility over a 1-week period. (Fisher's Exact Test, $p = 1$).

	**Male**	**Female**
	Mean ± SEM (n)	Mean ± SEM (n)
**pC1**	48 ± 3.1 (10)	6 ± 0.3 (8)
**pC2m**	31 (1)	NA
**pC2l**	44 ± 2.4 (10)	13 ± 0.5 (7)
**pCd-1**	15 (2)	8 ± 1.1 (2)
**pCd-2**	3 (10)	3 (10)
**pMN1**	NA	NA
**pMN2**	NA	1 (10)
**pMN3**	1 (10)	NA
**pLN**	1 (10)	NA
**aDN**	2 (10)	2 (10)
**SN**	1 (10)	NA
**TN1**	24 ± 0.8 (6)	NA
**Pr1**	1 (3)	NA
**Pr2**	1 (1)	NA
**Ms1**	1 (1)	NA
**Mt1**	1 (1)	NA
**Mt2**	1 (1)	NA
**Mt3**	1 (1)	NA
**Mt4**	1 (1)	NA
**Ab1**	1 (1)	NA

Table S1. Numbers of cells in *dsx*$^{+}$ single clusters. Related to Figure 1.

Figure	Full genotype
1C-1E, S1B	*y, w, hs-flp(22); FRTG13, UAS-mCD8::GFP/FRTG13, tubP-Gal80; dsx*^{Gal4}*/UAS-mCD8::GFP*
1C, 2A-2C, 2E-2G, 3B	*10xUAS-IVS-mCD8::GFP/VGlut*$^{dVP16.AD}$*; dsx*$^{Gal4.DBD}$*/+*
1D	*10xUAS-IVS-mCD8::GFP/+; dsx*$^{Gal4.DBD}$*/Trh-p65.AD*
2I-2K	*UAS-DenMark/VGlut*$^{dVP16.AD}$*; dsx*$^{Gal4.DBD}$*/+*
2L-2N	*UAS-Syb::GFP/VGlut*$^{dVP16.AD}$*; dsx*$^{Gal4.DBD}$*/+*
3A-3B, 5B-5C, S2A, S4A	*R35D04-p65.AD/10xUAS-IVS-mCD8::GFP; R22D06-Gal4.DBD/+*
3D-3G	*VGlut*$^{dVP16.AD}$*/VT029314-LexA; dsx*$^{Gal4.DBD}$*/UAS-CD4::spGFP1-10, lexAop-CD4::spGFP11*
3H-3I, S2E-S2J	*lexAop-CsChrimson::tdTomato, UAS-GCaMP6f/+; VGlut*$^{dVP16.AD}$*/VT029314-LexA; dsx*$^{Gal4.DBD}$*/+*
4, 7E-7G, S3B-S3R, S6C-S6E	*Otd-FLP/+; dsx*$^{Gal4.DBD}$*/+*
4, 7E-7G, S3B-S3R, S6C-S6E	*VGlut*$^{dVP16.AD}$*, UAS>stop>TNT/+*
4, 7E-7G, S3B-S3R, S6C-S6E	*VGlut*$^{dVP16.AD}$*, UAS>stop>TNT/+; dsx*$^{Gal4.DBD}$*/+*
4, 7E-7G, S3B-S3R, S6C-S6E	*VGlut*$^{dVP16.AD}$*, UAS>stop>TNT/Otd-FLP; dsx*$^{Gal4.DBD}$*/+*
5A-5C	*y, hs-flp; FRTG13, UAS-mCD8::GFP/FRTG13, tubP-Gal80; fru*^{NP21}*/UAS-mCD8::GFP*
5D-5E, S4A	*UAS-DenMark/+; GMR22D04-Gal4/+*
5F, S4D	*VT029314-LexA/+; GMR22D04-Gal4/UAS-CD4::spGFP1-10, lexAop-CD4::spGFP11*
5G-5H, S4E-S4K	*lexAop-CsChrimson::tdTomato, UAS-GCaMP6f/+;VT029314-LexA/+; GMR22D04-Gal4/+*
6F-6G	*lexAop-CsChrimson::tdTomato, UAS-GCaMP6f/+; VGlut*$^{dVP16.AD}$*/GH146-LexA; dsx*$^{Gal4.DBD}$*/+*
6H-6I	*lexAop-CsChrimson::tdTomato, UAS-GCaMP6f/+; VGlut*$^{dVP16.AD}$*/+; dsx*$^{Gal4.DBD}$*/Orco-LexA::VP16*
S2A	*VT029314-LexA/13xlexAop2-IVS-myr::GFP*
S2B	*VGlut*$^{dVP16.AD}$*/+; dsx*$^{Gal4.DBD}$*/UAS-CD4-spGFP1-10, lexAop-CD4-spGFP11*
S2C	*VT029314-LexA/+; UAS-CD4-spGFP1-10, lexAop-CD4-spGFP11/+*
S3A	*VGlut*$^{dVP16.AD}$*/Otd-FLP; dsx*$^{Gal4.DBD}$*/UAS>stop>mCD8::GFP*
S4B-S4C	*UAS>stop>mCD8::GFP/+; GMR22D04-Gal4/fru*^{FLP}

Table S3. Full genotype list. Related to Figures 1-7 and Figures S1-S4, S6.
