# Supplementary material for: A sex-specific switch between visual and olfactory inputs underlies adaptive sex differences in behavior
Source: Curr Biol. 2021 Mar 22;31(6):1175–1191.e6. doi: 10.1016/j.cub.2020.12.047 (PMC7987718; doi:10.1016/j.cub.2020.12.047)
Supplement: Document S2. Article plus supplemental information [file mmc5.pdf]

Article

Current Biology

A sex-specific switch between visual and olfactory inputs underlies adaptive sex differences in behavior

Graphical abstract

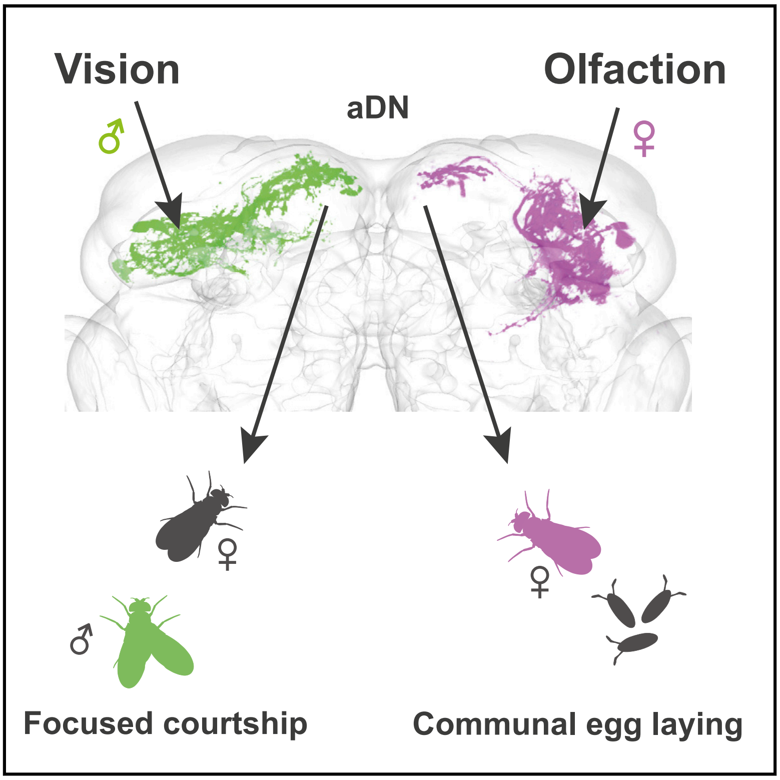

Highlights

- *doublesex*-expressing aDN neurons have sexually dimorphic inputs
- Male aDNs receive visual inputs via LC10 visual projection neurons
- Female aDNs receive olfactory inputs via the lateral horn
- aDNs control visual courtship pursuit in males and communal egg laying in females

Authors

Tetsuya Nojima, Annika Rings, Aaron M. Allen, ..., Jean-Christophe Billeter, Megan C. Neville, Stephen F. Goodwin

Correspondence

megan.goodwin@cncb.ox.ac.uk (M.C.N.), stephen.goodwin@cncb.ox.ac.uk (S.F.G.)

In Brief

How does the nervous system produce sex differences in behavior? Nojima et al. report a sexually dimorphic neuronal cluster in the *Drosophila* brain that receives sex-specific sensory information: visual signals required for courtship pursuit in males and olfactory signals required for communal egg laying in females.

Nojima et al., 2021, Current Biology *31*, 1175–1191
March 22, 2021 © 2021 The Authors. Published by Elsevier Inc.
https://doi.org/10.1016/j.cub.2020.12.047

Current Biology

CellPress
OPEN ACCESS

Article

A sex-specific switch between visual and olfactory inputs underlies adaptive sex differences in behavior

Tetsuya Nojima,[1,4] Annika Rings,[1,4] Aaron M. Allen,[1] Nils Otto,[1] Thomas A. Verschut,[2] Jean-Christophe Billeter,[2] Megan C. Neville,[1,*] and Stephen F. Goodwin[1,3,5,*]
[1]Centre for Neural Circuits and Behaviour, University of Oxford, Oxford OX1 3SR, UK
[2]Groningen Institute for Evolutionary Life Sciences, University of Groningen, Groningen, the Netherlands
[3]Twitter: @SFGLab
[4]These authors contributed equally
[5]Lead contact
*Correspondence: megan.goodwin@cncb.ox.ac.uk (M.C.N.), stephen.goodwin@cncb.ox.ac.uk (S.F.G.)
https://doi.org/10.1016/j.cub.2020.12.047

SUMMARY

Although males and females largely share the same genome and nervous system, they differ profoundly in reproductive investments and require distinct behavioral, morphological, and physiological adaptations. How can the nervous system, while bound by both developmental and biophysical constraints, produce these sex differences in behavior? Here, we uncover a novel dimorphism in *Drosophila melanogaster* that allows deployment of completely different behavioral repertoires in males and females with minimum changes to circuit architecture. Sexual differentiation of only a small number of higher order neurons in the brain leads to a change in connectivity related to the primary reproductive needs of both sexes—courtship pursuit in males and communal oviposition in females. This study explains how an apparently similar brain generates distinct behavioral repertoires in the two sexes and presents a fundamental principle of neural circuit organization that may be extended to other species.

INTRODUCTION

Sexually reproducing species exhibit sex differences in social interactions to boost reproductive success and survival of progeny. Comparing and contrasting the anatomy, activity, and function of sexually dimorphic neurons in the brain of males and females across taxa are starting to reveal the fundamental principles of neural circuit organization underlying these sex differences in behavior. A variety of alternative neuronal circuit configurations have been proposed to generate sexually dimorphic behaviors.[1] Many studies have identified sex differences in sensory inputs in various species; however, such differences in higher order brain circuits that organize species- and sex-specific instinctive behaviors in response to sensory cues are still poorly characterized.

Sex is determined early in an animal's development and initiates many irreversible sexual differentiation events that influence how the genome and the environment interact to give rise to sex-specific morphology and behavior. In *Drosophila*, selective expression of two sex determination transcription factors (TFs), Doublesex (Dsx) and Fruitless (Fru), define cell-type-specific developmental programs that govern functional connectivity and lay the foundations through which innate sexual behaviors are genetically predetermined.[2–6] Because both *fru*- and *dsx*-expressing neurons are essential for male and female reproductive behaviors, studies in the adult have focused on neurons that express these TFs to identify anatomical or molecular sex differences in neuronal populations.[7] This allows us to gain entry to the neural circuits underlying sex-typical behaviors and identify the neuronal nodes that control component behaviors and behavioral sequencing.

Dsx proteins, which are part of the structurally and functionally conserved Doublesex and Male-abnormal-3 Related Transcription factors (DMRT) protein family, are critical for sex-specific differentiation throughout the animal kingdom.[8] In the insect phylum, Dsx proteins act at the interface between sex determination and sexual differentiation, regulating a myriad of somatic sexual differences both inside and outside the nervous system.[9] The *dsx* gene has functions in both sexes: its transcripts undergo sex-specific alternative splicing to encode either a male- or female-specific isoform.[10,11] *dsx* expression is highly regulated in both male and female flies, as shown by its temporally and spatially restricted expression patterns through development, with only a select group of neurons expressing *dsx*.[12–17] The *dsx* gene is expressed in some 150 and 30–40 neurons per hemisphere in the male and female brains, which reside in 10 and 7 to 8 discrete anatomical clusters, respectively.[12,14,15,17,18] This restricted expression of *dsx* in higher order neurons in the brain suggests these neurons may act as key sex-specific processing nodes of sensory information.

To study the fundamental principles of neural circuit organization underlying sex differences in behavior, we identified and mapped dsx^+ sexual dimorphisms in the CNS. Our analyses revealed that all dsx^+ clusters are either sexually dimorphic or sex

This is an open access article under the CC BY license (http://creativecommons.org/licenses/by/4.0/).

specific; none are sexually monomorphic. To examine higher order processing differences between the sexes, we focused on the *dsx*+ anterior dorsal neuron (aDN) cluster, as it is present in both sexes yet has sexually dimorphic dendritic arborizations associated with sensory perception. We show that these anatomical differences lead to sex-specific connectivity, with male aDN inputs being exclusively visual, while female inputs are primarily olfactory. Finally, we show that this unique sexually dimorphic neuronal hub that reroutes distinct sensory pathways gives rise to functionally distinct social behaviors between the sexes: visual tracking during courtship in males and communal egg-laying site selection in females.

RESULTS

dsx-expressing neurons in the CNS are either sexually dimorphic or sex specific

To systematically investigate the anatomy of *dsx*-expressing neuronal clusters in the CNS of both sexes, we carried out a single cluster-level screen of *dsx*+ neurons employing genetic mosaic and intersectional approaches.[19,20] We used image co-registration to compare individually labeled male and female clusters to identify anatomical sexual dimorphisms (data available through Virtual Fly Brain).[21] After dissecting approximately 3,500 brains, we found that all *dsx*+ clusters examined in the brain showed anatomical sex differences in their cell numbers and/or neurite morphologies (Figure 1; Table S1; see Lee et al.,[12] Robinett et al.,[17] and Kimura et al.[18] for cluster nomenclature). A similarly detailed analysis of dimorphism in the ventral nerve cord (VNC) is described in Figure S1 and Table S1. Collectively, our single-cluster mapping demonstrates that *dsx*+ neuronal clusters in the CNS are either sex specific or sexually dimorphic, while none are sexually monomorphic.

dsx+ aDNs have sexually dimorphic input sites

dsx expression is restricted to a small number of sexually dimorphic higher order neurons in the brain in both sexes, suggesting they may act as key processing nodes of sensory information that subserve sex-specific behaviors. The sexually dimorphic *dsx*+ aDN cluster, consisting of two neurons per hemisphere with their somata located anteriorly in the superior lateral protocerebrum (SLP), is a striking example of this functional organization (Figure 1).

Overt sexual dimorphisms in sensory connectivity with the aDNs were apparent when examining their neurites. All aDN neurites are restricted to the ipsilateral protocerebra and do not cross the midline (Figures 2A–2H). Male and female laterally localized neurites terminate in noticeably different neuropils, exhibiting clear sexually dimorphic arborization patterns. Male neurites are restricted to the anterior optic tubercle (AOTu), one of the central-brain optic glomeruli through which visual signals are conveyed via visual projection neurons (VPNs) from the optic lobe to the central brain.[22–26] In the female, the lateral neurites are mainly in the posterior part of the superior lateral protocerebrum (pSLP), with minor processes in the superior clamp (SCL) and ventrolateral protocerebrum (VLP) (Figures 2A–2H). These neuropil regions receive multiple sensory inputs, including those from contact chemosensory, mechanosensory, olfactory, and auditory interneurons.[27–33] aDN medial neurites, in contrast, terminate in the same areas in both sexes, the superior medial protocerebrum (SMP) (Figures 2A–2C). These sex-specific neurite patterns make it a particularly interesting candidate for a node that could process sensory inputs differently between the sexes.

To determine the flow of information through the aDNs in males and females, we examined pre- and postsynaptic markers using a split-*Gal4* hemi-driver combination, *dsx*∩*VGlut*, which specifically labels the aDN in the brain.[34] In males, postsynaptic input (DenMark)[35] signals were detected in the AOTu (Figures 2I and 2K), while in females, input sites were observed in the pSLP/SCL/VLP regions (Figures 2J and 2K). In contrast, aDN presynaptic output sites (Syb::EGFP)[36] were restricted to the SMP in both sexes (Figures 2L–2N). These sex differences in dendritic arborization lead to dimorphic connectivity in aDN neurons and suggest they receive information from different sensory modalities in males and females.

Male aDNs receive inputs from visual projection neurons

aDNs receive extensive visual inputs from the AOTu in males, but not in females. Among more than 20 known classes of VPNs, a single class called the lobula columnar 10 (LC10) neurons send their axonal projections exclusively to the AOTu (Figure 3A).[22] A subpopulation of LC10 neurons, LC10a, regulates visual aspects of male courtship behavior, whereas no clear behavioral function has been characterized in females.[25] Intriguingly, the anatomy and physiological responses of these LC10 neurons are similar between the sexes.[25] These findings led us to hypothesize that males and females might receive the same visual stimuli through LC10 neurons but produce distinct behavioral responses due to sex differences in their central-brain connectivity.

We examined connectivity between LC10s and aDNs in males using an LC10a-specific transgenic driver (Figure S2A).[25] Co-registration of LC10a presynaptic sites with the postsynaptic dendritic fields of the aDNs revealed a clear overlap in males, but not in females (Figures 3B and 3C). GFP reconstitution across synaptic partners (GRASP)[37] also detected proximity between LC10a and aDN in males, but not in females (Figures 3D–3G); control males showed no GFP signals (Figures S2B and S2C). These observations suggest that LC10a neurons connect to aDNs in males, but not in females. To corroborate the absence of LC10a inputs to the aDN in females, we plotted aDN and LC10a neurons in the same reference space using connectivity information from the densely reconstructed hemibrain connectome via the neuPrint (v1.1) database (https://neuprint.janelia.org/).[38] We confirmed that female aDNs do not receive input from any of the approximately 450 LC10a single neurons annotated in the hemibrain dataset (Figure S2D).

To test whether aDN and LC10a neurons are functionally connected in males, we light-stimulated LC10a neurons expressing the red-light-activated channelrhodopsin *CsChrimson*[39] while simultaneously recording fluorescence from aDNs expressing *GCaMP6f*.[34,40] Initially, we did not see a response in aDNs in either sex with different stimulation patterns (Figures 3H and 3I, top, and S2E–S2H), in apparent contrast to their anatomical connection revealed by GRASP (Figures 3D and 3E). We

CellPress
OPEN ACCESS

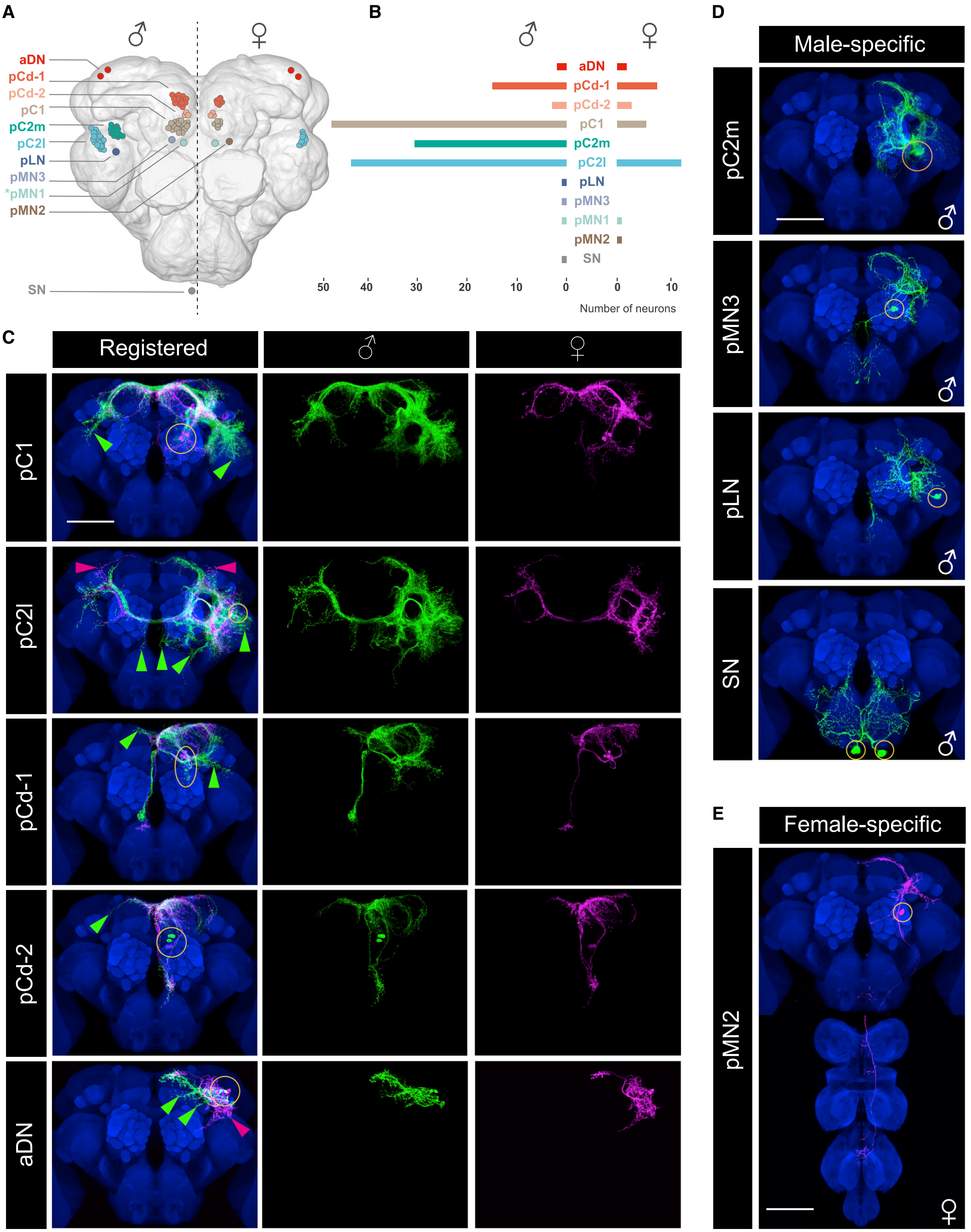

(legend on next page)

reasoned that the response in aDNs might be masked by inhibition from other upstream neurons, potentially dependent on the male's courtship arousal state.[41] To test this possibility, we blocked inhibitory inputs by applying picrotoxin (PTX), an inhibitor of glutamate-gated chloride channels and gamma-aminobutyric acid (GABA)-A receptors, and CGP54626, a GABA-B receptor antagonist, to the brain 15 min before the imaging experiment.[41–43] After blocking inhibition, we observed a significant calcium response in male aDN dendrites during stimulation of LC10a, which increased with a higher concentration of PTX (Figures 3H and 3I). The apparent axonal responses in males were not significant, probably due to larger variability, with only some of the dendritic inputs leading to synaptic output from axon terminals. Light-evoked responses were male specific (Figures 3H and 3I) and could not be detected in controls (Figures S2I and S2J), even though both sexes showed spontaneous activity after application of PTX (data not shown), probably as a result of disinhibition of other inputs to aDNs. Therefore, anatomical and physiological results confirm that the male, but not female, aDNs receive visual inputs from LC10a VPNs. This striking dimorphism in aDN input sites generates different routing of sensory information between males and females.

Male aDNs function in visually guided courtship behavior

LC10a VPNs have been shown to extract object motion relative to the background, recognizing the current position of a potential mate during courtship.[25] We therefore tested whether aDNs might be a necessary downstream target to mediate their role in courtship. We blocked evoked transmission from aDNs in male flies using expression of a *TNT* transgene[44] and tested their courtship behavior. To specifically target aDNs, we combined the split-*Gal4* hemi-driver *dsx*∩*VGlut* and a brain-specifically expressed FLP recombinase (*Otd-FLP*)[45] with a *Gal4/FLP*-responsive TNT effector (Figure S3A).[46]

Courtship intensity and copulation success were markedly reduced in aDN-silenced males (*dsx*∩*VGlut*∩*Otd* > *TNT*) compared with genetic controls (Figures 4A and 4B). However, they exhibited normal latencies to initiate courtship (Figure 4C). Pronounced abnormalities were apparent when examining videos of aDN-silenced males (Videos S1 and S2). To quantify specific behavioral deficits, we trained an automated behavioral annotation system to detect the following male courtship behaviors: wing extension (a courtship-specific behavior used to generate song), approaching, facing, turning, circling, and contact (STAR methods). We observed a reduction in both approaching and facing indices and a trend for an increase in the contact index (a measure of the minimum distance between the male and female; Figures S3B–S3D). Importantly, all other indices examined were unaffected, suggesting specific rather than general deficits (Figures S3E–S3G). As approaching and facing behaviors both depend on being able to localize the female, we hypothesized that aDN-inactivated males have difficulties tracking females. We therefore measured how the male orients toward the female during courtship by examining the position of the female relative to the male while he was unilaterally extending his wing. aDN-silenced males exhibited an increase in the amount of misdirected courtship behavior (i.e., the male extended his wing when the female was not in front of him; Figures 4D and S3H; Videos S1 and S2). This defect is more discernible by examining the facing angle (Figures 4E and 4G) and distance between the male and female (Figures 4F and 4H), which were both increased compared to controls, a phenotype reminiscent of LC10a-silenced males.[25] The minimum distance, on the other hand, was decreased (Figure 4I), showing that aDN-silenced males position themselves at a broader range of distances while singing to the female. These manipulations demonstrate that aDN-silenced males were impaired in their ability to orient and track female movement during courtship. These deficits are linked to a difficulty in locating and following the female, not a reduced motivation to court. aDN-silenced males are not blind in general, as they showed the same preference for light as controls in a phototaxis assay (data not shown). Our data are consistent with the male aDN playing a role in motion detection during male courtship behavior.

While courting, males unilaterally extend their wing closest to the female (ipsilateral wing) to produce courtship song.[47] Although aDNs and LC10a neurons in males are both involved in visually guided courtship pursuit of the female, their roles in wing extension appear to differ. aDN-silenced males displayed normal levels of unilateral wing extension (Figure S3I) and, in contrast to LC10a-silenced males,[25] exhibited normal ipsi- versus contralateral wing choice (Figure S3J). aDN-silenced males did, however, display an increase in bilateral wing extension (Figures 4J and S3K). This increase was due to an increase in the number of bouts (Figure 4K) and not the bout length (Figure S3I). To test whether these phenotypes depend on visual cues, we tested courtship behavior in the dark and found no significant differences between aDN-silenced males and controls

Figure 1. Comprehensive single cluster-level mapping of *dsx*-expressing neurons in the brain

(A) A schematic drawing of *dsx*-expressing neurons in the male (left) and female (right) brain.

(B) Mean *dsx*$^+$ neuron numbers per cluster in the male and female central brain (see Table S1 for more detail). pC1, pC2l, and pCd-1 have more cells in males than in females, while pCd-2 and aDN have the same number of cells in both sexes (see also Table S1).

(C) Sexually dimorphic *dsx*-expressing neuronal clusters in the brain individually visualized by MARCM, as driven by *dsx*^{Gal4}, except for the aDN cluster, which was visualized by a split-*Gal4* combination (for simplicity, only the unilateral cluster is shown). The male (green) and female (magenta) corresponding clusters are co-registered onto a template brain (blue; left). The male (middle) and female (right) clusters are shown individually. Yellow ellipses show cell body positions. Male- and female-specific neurite arbors are indicated with green and magenta arrowheads, respectively. NB: pMN1 is not shown, as we failed to label a single cell in isolation. We observed five sexually dimorphic clusters with male-specific neurite arbors (pC1, pC2l, pCd-1, pCd-2, and aDN) and two with both male- and female-specific arbors (pC2l and aDN).

(D) Male-specific *dsx*$^+$ clusters/neurons in the brain individually visualized by MARCM, except for the SN neuron, which was visualized by a split-*Gal4* combination.

(E) The female-specific *dsx*$^+$ pMN2 neuron in the brain visualized by MARCM.

(C–E) Scale bars: 50 μm. See also Figure S1. See Table S3 for the full genotypes.

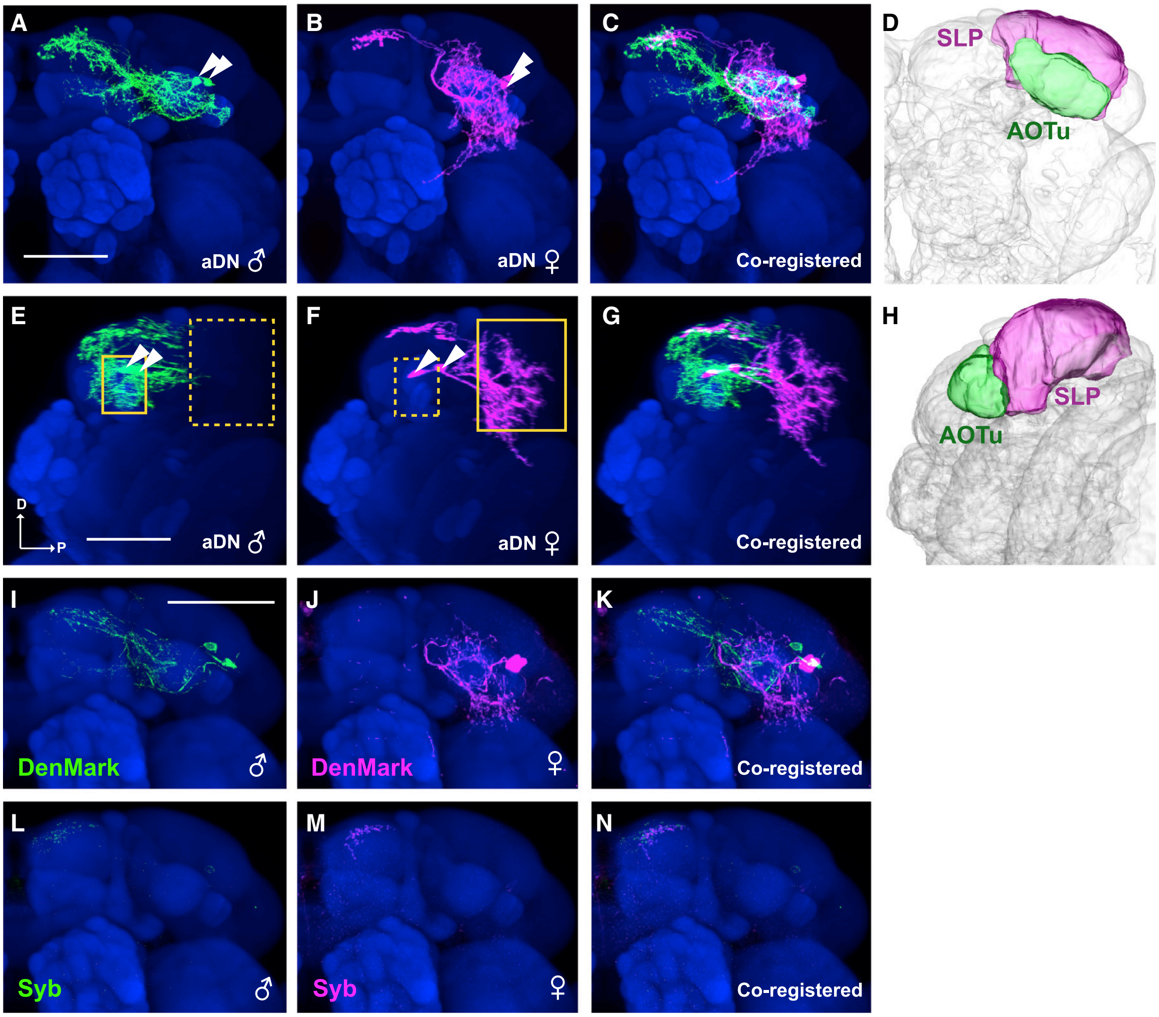

Figure 2. *dsx*⁺ aDNs have sexually dimorphic dendritic input sites

(A–C) The male (green) and female (magenta) aDNs, visualized using the $dsx^{Gal4.DBD}$ and $VGlut^{dVP16.AD}$ hemi-driver combination, co-registered onto a template brain (blue). Arrowheads indicate cell bodies. We note that we did not find any consistent morphological difference in neurite distribution of individual aDNs within either sex (data not shown).

(D) A 3D-rendered schematic of the neuropil regions, AOTu (green) and SLP (magenta).

(E–G) Lateral view images of (A)–(C). Small and large yellow boxes in (E) and (F) indicate the positions of AOTu and SLP, respectively. Solid and dashed lines of the boxes indicate the presence and absence of neurites in the defined neuropil regions, respectively. "D" and "P" in (E) represent dorsal and posterior, respectively.

(H) A lateral view image of (D).

(I–N) DenMark (I–K) and Syb (L–N) signals driven by the $dsx^{Gal4.DBD}$ and $VGlut^{dVP16.AD}$ hemi-driver combination in the male (green; I, K, L, and N) and female (magenta; J, K, M, and N) brain.

(A–N) Scale bars: 50 μm. See Table S3 for the full genotypes.

(Figures S3M–S3R), suggesting that, in the absence of visual cues, aDN neurons are not necessary for normal courtship behavior. Bilateral thermogenetic activation of aDNs in solitary males did not induce any wing extensions (data not shown), in contrast to LC10a bilateral activation, which induces unilateral wing extensions.[25] Our findings suggest that visually guided courtship behavior through LC10a VPNs consists of two separable components—visual tracking and wing choice—likely mediated by different sets of downstream neurons. aDN is involved in the former, but not the latter. We therefore set out to identify additional downstream targets of LC10a that might control wing choice.

The *fru*-expressing AL5a cluster is downstream of LC10a VPNs

To identify additional neurons downstream of LC10a, we examined a candidate neuronal cluster, AL5a, which innervates the AOTu and expresses the sex-determination gene *fru*.[21,48,49] Interestingly, when AL5a is unilaterally activated, male flies frequently display unilateral wing extension,[50] a behavioral phenotype reminiscent of unilateral LC10a activation.[25] Based on these observations, we hypothesized that AL5a (also known as aSP-I and aSP11)[21,49] is downstream of LC10a.

Co-registration of LC10a with the AL5a cluster revealed clear overlap in the AOTu region in males and females (Figures 5A–5C

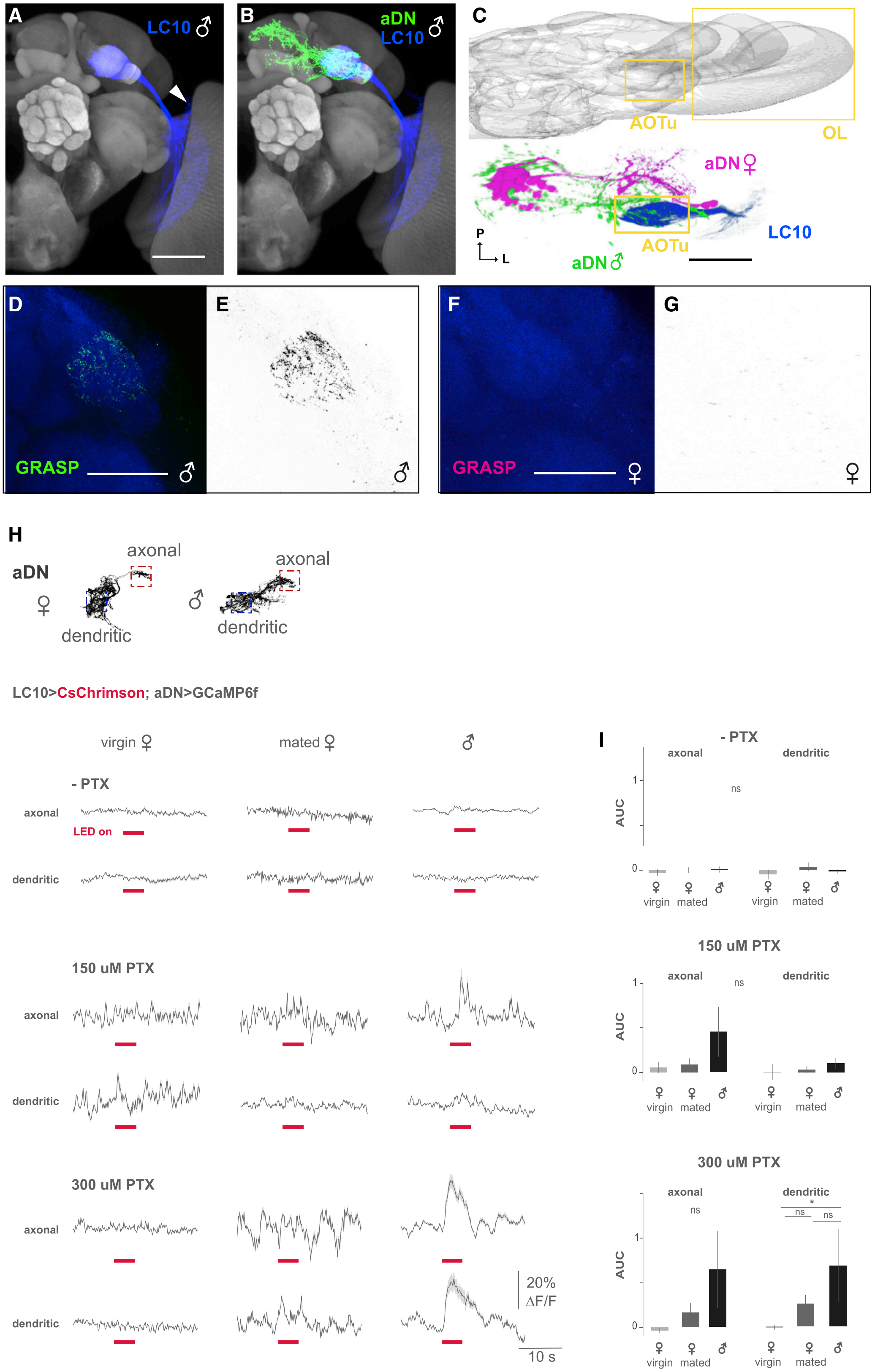

(legend on next page)

and S4A). To investigate the anatomical and physiological connectivity between LC10a and AL5a, we identified a *Gal4* line that is expressed in AL5a neurons and confirmed *fru*$^+$ AL5a AOTu innervation (Figures S4B and S4C).[21,49] We found that AL5a input sites are associated with the AOTu (Figures 5D and 5E), and GRASP detected robust signals in both sexes between LC10a and AL5a neurons in the dorsal side of the AOTu (Figures 5F and S4D). Our findings suggest that the AL5a neurons connect with LC10a neurons in both sexes.

To confirm that LC10a and AL5a neurons are functionally connected, we optogenetically activated the LC10a neuronal population while simultaneously imaging calcium transients in the dendritic arbors of AL5a neurons in the AOTu. We observed a large calcium response in AL5a input sites in both sexes (Figures 5G, 5H, and S4E–S4K), suggesting that, unlike aDNs, AL5a receives inputs from LC10a in both sexes. Together, these data indicate AL5a neurons are downstream of LC10a neurons in both sexes, whereas connectivity from LC10a to aDN is male specific.

Female aDNs receive multimodal sensory inputs

While males utilize aDNs to process visual inputs, female aDNs have alternative inputs. Using ultrastructural connectivity, we found female aDNs process information from multiple sensory modalities, the most prominent of which are olfactory and thermo- and hygrosensory cues. We examined direct synaptic connectivity between annotated aDNs and circuit elements in the female hemibrain electron microscopy (EM) dataset (Figure 6A). In agreement with our light imaging results, we found the dendrites of aDNs span multiple neuropils in females (Figures 6A and 6B), whereas the axonal region is restricted to the SMP neuropil. Although over 80% of aDN inputs and outputs were restricted to the dendritic and axonal regions, respectively, many axo-axonic inputs and dendro-dendritic outputs were found (Figure 6C). We next examined the identity of the neurons pre- and postsynaptic to the aDN (Figures S5A and S5B; Table S2). We focused on the 30 neuron types with the largest contribution to the synaptic input budget (Figure 6D). A clear connection between olfactory input processing neurons and the aDN was apparent, as 9 of the neuron types are associated with the lateral horn (LH) and receive inputs from olfactory projection neurons (PNs) (Figures 6D and S5A). The LH is involved in innate odor responses as well as thermo- and hygrosensory responses.[51] We also identified direct inputs from potential olfactory and thermo-/hygrosensory PNs into the aDN, including the multi-glomerular VP5+ adPN, known to have extensive innervation in the humid air sensing VP5 glomerulus in the antennal lobe (AL) as well as the subesophageal zone (SEZ) (Figures 6D and S5C).[52] In addition to antennal sensory inputs, multiple neurons ascending through the lateral antennal lobe tract (lALT) synapse onto the aDN (lALT1 and lALT2; Figures 6D and S5D). The morphology of these lALT-tract axons is reminiscent of contact chemosensory and mechanosensory ascending neurons from the VNC.[31,32] Together, this indicates that female aDNs receive multimodal sensory inputs, many of which are pre-processed olfactory and thermo- and hygrosensory cues inputting through the LH.

Female aDNs receive functionally relevant olfactory inputs

To analyze functional connectivity between the olfactory system and the aDNs, we expressed the optogenetic activator *CsChrimson* under the control of a broad antennal-lobe projection neuron driver line, *GH146-LexA*, which expresses in two-thirds of all second-order olfactory PNs,[53,54] and monitored the activity of aDNs expressing *GCaMP6f* upon light stimulation. When PNs were optogenetically activated, female, but not male, aDNs showed an increase in fluorescence in both their dendritic and axonal compartments (Figures 6E–6G), with similar responses in both virgin and mated females. These responses confirm that female aDNs are downstream of the antennal-lobe PNs and, importantly, that this functional connectivity is female specific. To test whether this sex-specific response was at least in part olfactory in origin, we expressed the optogenetic activator *CsChrimson* under the control of a broad olfactory sensory neuron (OSN) driver line, *Orco-LexA*, which expresses in most OSNs in the basiconic and trichoid sensilla.[55] We found that female, but not male, aDNs showed an increase in calcium signals during stimulation of OSNs. Intriguingly, these responses were more robust in 24–48 h post-mated females compared to virgin females, suggesting a change in aDN responses to odors after mating (Figures 6H and 6I) and predicting a role for aDN neurons

Figure 3. Male, but not female, aDN is a downstream cluster of LC10a

(A) Male LC10a cluster labeled by OL0019B (*R35D04-p65.AD/R22D06-Gal4.DBD*; blue) registered onto a template brain (gray). White arrowhead indicates the cell bodies. Scale bar: 50 μm.

(B) Male aDN (green) and LC10a (blue) are co-registered onto a template brain (gray).

(C) A dorsal view image of the brain is shown in the upper panel, and the male (green) and female (magenta) aDN and sexually monomorphic LC10a (blue) are co-registered and shown in the same view as above in the lower panel. Yellow boxes indicate the AOTu and optic lobe (OL). P and “L” represent posterior and lateral, respectively. Scale bar: 30 μm.

(D–G) GRASP experiments between LC10a and aDN. Male (D and E) and female (F and G) AOTu regions are shown. 10 samples in each sex were observed. Only male samples showed GRASP-positive fluorescence. Scale bars: 30 μm.

(H) Black and white insets: maximum projections of confocal stacks of male aDNs; red boxes indicate the axonal and blue boxes the dendritic recording regions. Mean (dark gray line) and standard error (SE) (light gray shaded area) of ΔF/F in aDN axonal and dendritic compartments in response to a 5-s (40-Hz, 10-ms pulses) red light-emitting diode (LED) optogenetic stimulation (stimulus time indicated by pink bar) of LC10a neurons expressing CsChrimson in virgin females (left), mated females (middle), and males (right) are shown; top: without picrotoxin (−PTX) and CGP54626 are shown (n for axonal/dendritic = virgin females 10/10, mated female 13/12, and male 24/18); middle: 150 μM picrotoxin and 50 μM CGP54626 are shown (n for axonal/dendritic = virgin females 8/8 virgin female flies, mated female flies 18/19, and male flies 13/14); bottom: 300 μM picrotoxin and 50 μM CGP54626 are shown (n for axonal/dendritic = virgin females 12/11 virgin female flies, mated female flies 13/14, and male flies 16/13).

(I) Mean and SE of the area under the ΔF/F curve (AUC) from beginning until 1 s after the end of the stimulus for experiments in (H). Not significant (ns) p.adj > 0.05; *p.adj < 0.05 by Mann-Whitney-U-test adjusted for multiple comparisons with the Holm method.

See also Figure S2. See Table S3 for the full genotypes.

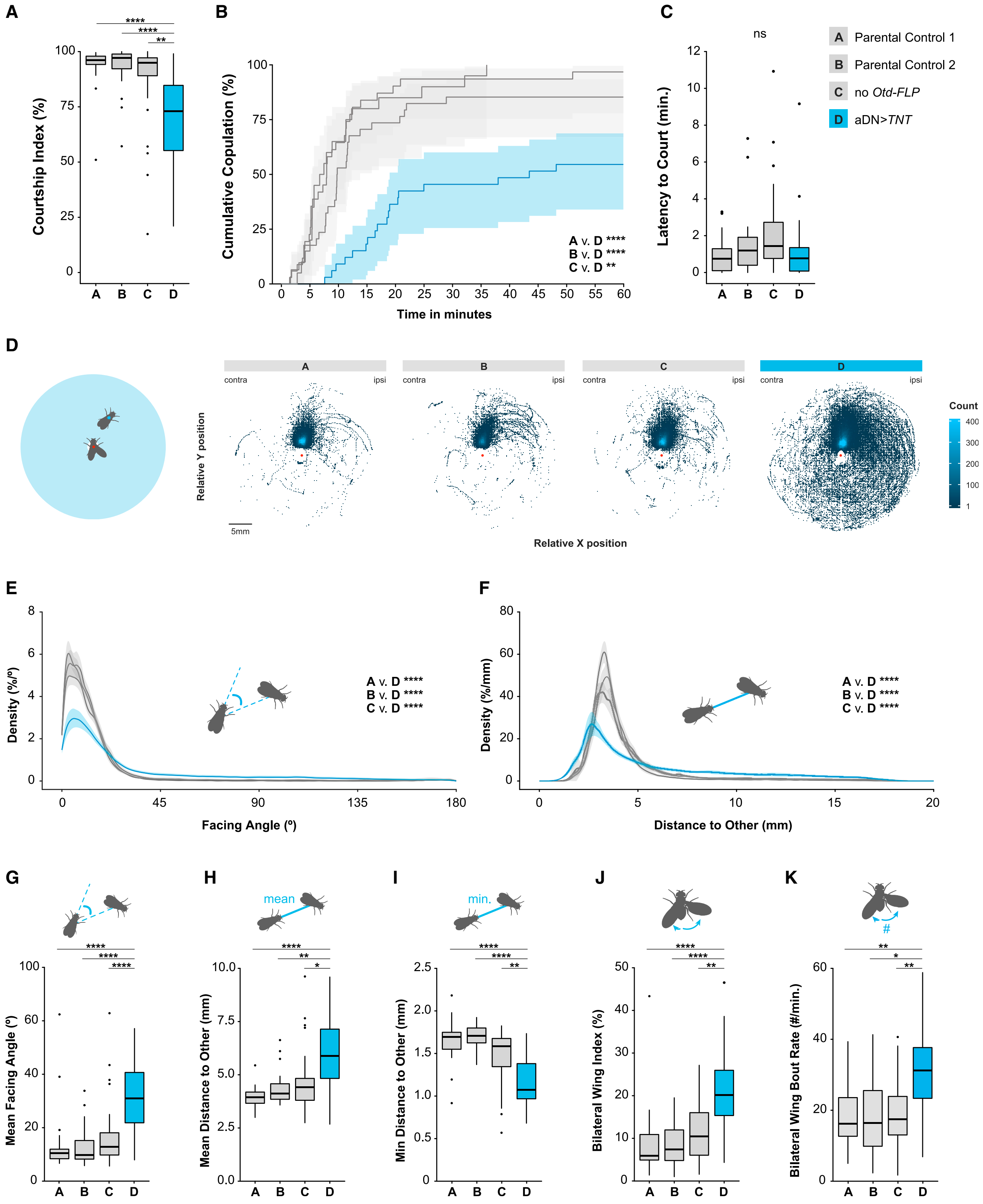

(legend on next page)

CellPress
OPEN ACCESS

in female post-mating behavior. These physiological data, in conjunction with the pattern of female-specific aDN connectivity in the brain, confirm that female aDNs respond to olfactory rather than visual input.

The female aDNs play a role in egg-laying site selection

When examining aDN connectivity, we found striking recurrent connectivity between aDNs and recently identified egg-laying circuitry (Figures 6D and 7A–7D).[56] Indeed, the neuron with the most synaptic inputs to the aDNs, with axo-axonic connections making up 15.5% of the axonal input budget, is the oviposition excitatory neuron oviEN, which mediates external sensory signals involved in egg-laying site selection in mated females (Figures 7A and S5A).[56] oviEN and aDN cell bodies are distinct and derived from different neuroblasts; however, their neuronal processes have nearly identical layouts (Figure 7B), suggesting they may integrate similar information (e.g., LH pre-processed olfactory). oviENs are excitatory cholinergic neurons, whereas aDNs are glutamatergic and could be inhibitory, excitatory, or both depending on postsynaptic receptor expression,[57,58] suggesting there may be differences in the way these two types of neurons process sensory information. The oviposition inhibitory neuron (oviIN) also forms synapses onto the axons of the aDNs from both the ipsilateral and contralateral hemispheres (Figure 7C), together accounting for 18.4% of the aDNs' axonal budget (Figure 6D). The oviIN conveys information about female mating status, potentially inhibiting aDN axonal output in virgin females only.[56] We found that aDNs directly output onto oviDNa, a female-specific descending neuron controlling egg laying (Figures 7A and 7D),[56] and an uncharacterized neuron SMP156, which appears highly integrated into the egg-laying circuitry (Figure 7A). aDNs devote the largest proportion of their axonal budget to SMP156, contributing the highest fraction to SMP156s inputs (5.7%). The oviEN contributes the 2nd highest (1.8%) to the SMP156 inputs, with oviIN neuron inputs being less prominent (0.86%). SMP156 outputs across both hemispheres in the inferior bridge (IB), a neuropil that spans the midline, implying it is involved in comparison and analysis of signals from different directions (Figures S6A and S6B).[59] The prominent connectivity of SMP156 neurons with egg-laying circuitry suggests it might act as an integrator of egg-laying signals.

The connections between aDNs, egg-laying circuitry, and the olfactory system, as well as their mating state-dependent responses to olfactory signals, predict a role in the social aspects of egg laying. In a social context, females rely on olfactory pheromonal cues deposited by resident flies at shared egg-laying sites.[60] We tested the hypothesis that the aDNs are necessary for this female-specific social and reproductive behavior by giving mated females the choice to lay eggs either on a patch marked with male pheromone extracts or a patch devoid of social cues (Figure 7E). Male pheromones attracted control females to lay their eggs on the pheromone-marked patch, while females in which aDNs where silenced by *TNT* did not distinguish between patches marked with pheromones or those devoid of them, resulting in a significant difference among genotypes (Figure 7F). Moreover, as silencing of aDNs did not have an effect on the number of eggs laid (Figure 7G) but did on the preference for pheromone-marked egg-laying sites, these data indicate that aDNs have a specific role in egg-laying site selection and not on fecundity. Finally, we asked whether the aDNs play a role in female pre-mating behaviors. Copulation latency and overall fertility were unaffected in aDN-silenced virgin females (Figures S6C–S6E). These data suggest a specific role for female aDNs in egg-laying site selection based on olfactory cues rather than a general role in mating behavior.

DISCUSSION

We have identified a small cluster of two neurons per hemisphere in the central brain, which reconfigures circuit logic in a sex-specific manner. Perhaps most surprising is the seemingly unrelated behaviors these equivalent neurons control in each sex—visual tracking during courtship in males and communal egg laying in females. Ultimately, these circuit reconfigurations lead to the

Figure 4. Silencing male aDN alters visually guided courtship behavior

(A–K) In boxplots, boxes represent 1st to 3rd quartile, bar represents the median, lower whisker represents the smallest value at most 1.5 * inter-quartile range from the 1st quartile, upper whisker represents the largest value at most 1.5 * inter-quartile range from the 3rd quartile, points represent data points beyond the whiskers.

(A) Courtship index (%; $F_{(3,114)} = 16.8$; p.adj < 0.0001).

(B) Cumulative proportional copulation (% ± 95% confidence intervals) over a 60-min time period ($\chi^2_{(3)} = 38.2$; p.adj < 0.0001).

(C) Latency to court (minutes; $F_{(3,114)} = 3.04$; p.adj = 0.42).

(D) Heatmap of the relative position of the female to the male (red dot) while the male's wing was extended. A schematic representation of the behavior is on the far left. For each genotype, the female's relative location during contralateral extensions is on the left and ipsilateral on the right. The color of each square represents the number of occurrences (non-occurrences are white).

(E–K) Schematic representations of the behaviors are inset.

(E) Mean probability density functions (%/° ± 95% confidence intervals) of the facing angle (°) of the male.

(F) Mean probability density functions (%/mm ± 95% confidence intervals) of the distance to other (mm).

(G–K) Summary characteristic for each male during the courtship period.

(G) Mean facing angle ($F_{(3,114)} = 18.5$; p.adj < 0.0001).

(H) Mean distance to other ($F_{(3,114)} = 15.6$; p.adj < 0.0001).

(I) Minimum distance to other ($F_{(3,114)} = 24.4$; p.adj < 0.0001).

(J) Bilateral wing index ($F_{(3,114)} = 17.5$; p.adj < 0.0001).

(K) Bilateral wing bout rate ($F_{(3,114)} = 9.22$; p.adj < 0.001).

Full genotypes: (A) *Otd-FLP/+; dsx$^{Gal4.DBD}$/+* (n = 31); (B) *VGlut$^{dVP16.AD}$, UAS > stop > TNT/+* (n = 20); (C) *VGlut$^{dVP16.AD}$, UAS > stop > TNT/+; dsx$^{Gal4.DBD}$/+* (n = 34); (D) *VGlut$^{dVP16.AD}$, UAS > stop > TNT/Otd-FLP; dsx$^{Gal4.DBD}$/+* (n = 33). *p.adj < 0.05; **p.adj < 0.01; ***p.adj < 0.001; ****p.adj < 0.0001 by t test (B and G–K) or log rank test (C) or Kolmogorov-Smirnov test (E and F) and adjusted with the Holm method. ns, p.adj > 0.05. See also Figure S3 and Videos S1 and S2. See Table S3 for the full genotypes.

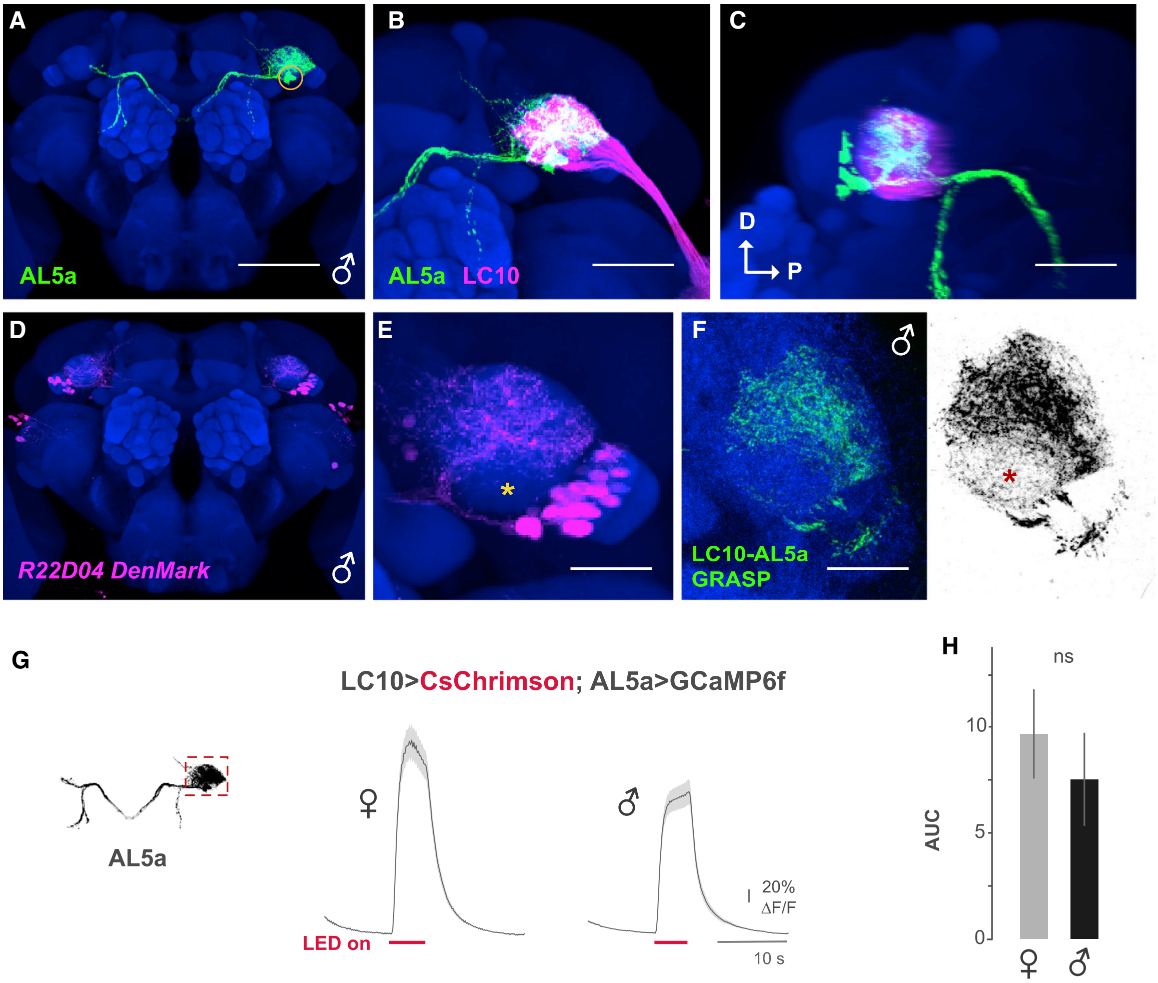

Figure 5. *fru*-expressing AL5a is an additional downstream cluster of LC10a

(A) A neuroblast clone of the male AL5a as visualized by *fru*^{NP21}-driven MARCM. Scale bar: 50 μm.

(B) Male AL5a (green) and LC10a (magenta) are co-registered onto the template brain (blue). Scale bar: 30 μm.

(C) A lateral view image of (B). Scale bar: 30 μm.

(D) Expression pattern of *R22D04-Gal4*-driven *DenMark* in the male.

(E) The AOTu region of (D) is magnified. AL5a input sites are notably most intense in the dorsal side of the AOTu, whereas the ventral side showed minimal, if any, labeling. A yellow asterisk indicates the absence of dense innervation of AL5a dendrites. Scale bar: 30 μm.

(F) GRASP experiment between LC10a and AL5a in the male in color (left) and black and white (right). A red asterisk indicates the absence of intense GFP signals. 10 samples were observed. All samples showed GRASP-positive fluorescence. Scale bar: 30 μm.

(G) Black and white inset: maximum projection of a confocal stack of mCD8::GFP-labeled AL5a in a male. Recording region in the AOTu for calcium imaging is indicated by a gray box. Mean (dark gray) and SE (light gray) of ΔF/F in AL5a neuron input sites expressing *GCaMP6f* under control of the *R22D04-Gal4* driver in response to 5 s optogenetic activation (pink bars) of LC10a neurons in females (left, n = 7) and males (right, n = 13).

(H) Mean and SE of the AUC for experiments in (G).

ns, p.adj > 0.05 by Mann-Whitney U-test adjusted for multiple comparisons with the Holm method. See also Figure S4. See Table S3 for the full genotypes.

same end result—an increase in reproductive success. Our findings highlight a flexible strategy used to structure the nervous system, where relatively minor modifications in neuronal networks allow each sex to respond to their social environment in a sex-appropriate manner.

The behavioral function of the male aDN cluster appears to be related to visual aspects of courtship behavior. A set of visual projection neurons, LC10a, was previously identified as involved in tracking and following behaviors in the male during courtship; however, no apparent sex differences in their anatomy or their physiological responses to visual stimuli were detected.[25] It would seem these sex differences in behavior arise from the sex-specific downstream connectivity of LC10a neurons in the central brain. Here, we identify aDNs connecting downstream to LC10a in males only. aDN inactivation mirrors visual tracking defects displayed upon LC10a inactivation (Figure 4); therefore, the male aDN cluster confers sex specificity to visually guided tracking of females during courtship.

We additionally identified AL5a neurons to be downstream of LC10a in both sexes. Interestingly, it has been reported that

CellPress
OPEN ACCESS

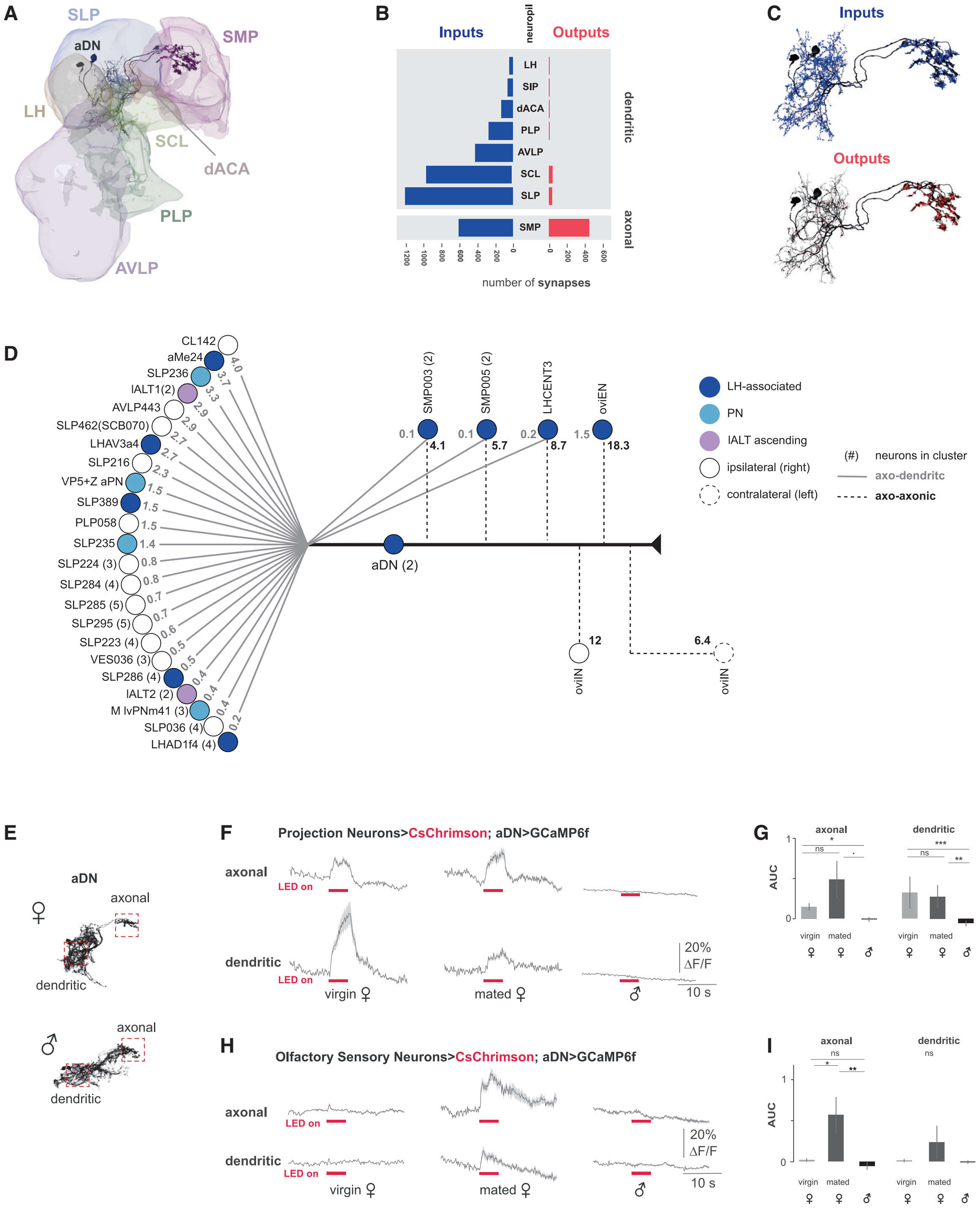

Figure 6. Female aDN neurons are downstream of olfactory projection neurons

(A) 3D representation of reconstructions from volumetric EM data of aDN (black; BodyIDs: 541347811 and 604070433) and synaptic neuropils in the right-brain hemisphere. The neuropils innervated by dendrites, SLP, SCL, LH, posterior lateral protocerebrum (PLP), dorsal accessory calyx (dACA), and anterior ventrolateral protocerebrum (AVLP), as well as the axon innervated SMP are shown.

(B) Synapse number of aDN inputs (postsynapses) and outputs (presynapses) by neuropil in blue and red, respectively.

(C) Spatial distribution of aDN inputs (top; postsynapses: blue) and outputs (bottom; presynapses: red).

(D) Synaptic connectivity between female aDN and upstream neurons. The 30 top neuron types with most synaptic connections to aDN are shown. The median percentage of contribution to each aDN's input budget (i.e., the contribution of a single upstream neuron's synaptic input as a fraction of the total number of inputs to aDN and, for clusters, the median of contributions) is indicated. Dendritic and axonal input budgets are shown in gray and black, respectively. For clusters types

(legend continued on next page)

AL5a is likely upstream of the *fru*$^{+}$ cluster Lv2/pIP-b/pIP8,[48,49] thought to exchange and integrate visual information from the right and left hemispheres of the brain. This male-specific connectivity is compatible with a potential role for AL5a in mediating visual information necessary for wing choice during courtship, a behavior these neurons have been shown to elicit when activated.[50]

The two LC10a downstream clusters we identified, aDN and AL5a, also show differences in their anatomical connectivity and physiological responses. Whereas AL5a is downstream of LC10a in both sexes, aDN is only connected to LC10a in the male. Despite direct anatomical connectivity between LC10a and aDN in males (Figure 3), functional connectivity was only uncovered under conditions of pharmacological disinhibition. This observation might hint at inhibitory modulation of aDN that depends on the male's internal state, e.g., his mating drive, or additional cues that influence his courtship arousal. A previous study found that, in sexually satiated males, calcium responses in courtship "decision-making" P1 neurons were absent when stimulating upstream neurons but could be restored to the levels observed in naive males by application of PTX.[41] It is tempting to speculate that inhibition in the LC10a → aDN pathway is similarly linked to sexual arousal. In contrast, AL5a responses to LC10a stimulation occurred in the absence of PTX and were markedly larger in AL5a than in aDN (compare Figures 3H, 3I, 5G, and 5H). The variation in calcium signals could be due to the considerable difference in cell numbers comprising each cluster (2 aDN versus 24 AL5a) or due to inputs from different AOTu regions. aDNs sample from the whole glomerulus region, whereas the AL5a cluster is restricted to the dorsal part of the AOTu, suggesting they extract information from broad versus specific parts of the visual field, respectively. Future investigation will be aimed at linking the clusters' anatomical differences with their differential processing of visual information to facilitate distinct behavioral roles.

In females, the aDN cluster does not receive visual information but appears to sample from a range of sensory modalities, with information received via the antennal lobe dominating its inputs, suggesting its involvement in a complex behavior requiring multisensory integration. One such behavior is female egg-laying site selection, which is critical to the success of offspring.[61] For *Drosophila*, offspring survival rates depend on the selection of oviposition sites that are shared with conspecifics, a process known to rely on olfaction.[60] We have shown that aDNs are highly integrated into circuitry known to regulate oviposition (Figure 7A).[56] The excitatory oviEN, which is anatomically similar to the aDNs, responds to information about substrate suitability via gustatory and mechanosensory cues in the legs and directly influences aDN output. Silencing oviEN function suppresses egg laying itself, whereas silencing aDN does not affect the overall number of eggs laid. Instead, aDN-silenced females are no longer able to show a preference to lay eggs communally, losing a female-specific social behavior essential for offspring survival.[62] While both oviEN and aDN output directly onto the oviposition motor program (through oviDNs), oviENs are the largest contributors to oviDN dendritic budgets, with aDN being relatively minor contributors. Thus, the aDN cluster acts as a modulator of egg laying choice, whereas the oviEN more generally affects the mechanics of egg laying.

As the oviposition of fertilized eggs is a female behavior that can only be displayed after mating, the behavioral programs required are likely inhibited in virgin females. The activity of the inhibitory neuron oviIN depends on female mating status and thus appears to act as a general inhibitor of egg-laying circuitry in virgin females.[56] oviINs form axo-axonic synapses with both the aDN and oviEN, suggesting they gate their outputs by presynaptic inhibition in a state-dependent manner.[63] Intriguingly, as both oviEN and oviIN form axo-axonic synapses with aDN, this suggests a potential gating mechanism by which their relative strengths inhibit or facilitate output from aDN onto downstream targets.

Consistent with aDNs' behavioral function in egg-laying site selection, a female post-mating behavior, we found differences in the aDN physiological responses in mated versus virgin females. Stimulation of OSNs resulted in significantly stronger aDN calcium responses in mated females compared to virgins (Figures 6E–6I). This finding might hint at a state-dependent inhibition of olfactory inputs into aDN in females, potentially analogous to the inhibition of visual inputs to aDN observed in males. The difference in physiological responses between mated and virgin females was not observed when stimulating PNs, which are downstream of OSNs but upstream of aDN. There are different possible explanations for this discrepancy, including differences in the populations of neurons targeted by the driver lines used to target PNs versus OSNs or inhibition in virgin females occurring at the level of OSN to PN connectivity; therefore, activating PNs directly bypasses the state-dependent inhibition. In addition to state-dependent effects, there also seemed to be differences in the calcium responses in different neuronal compartments (Figures 6E–6I). This finding could be explained by the position of the input synapses of different upstream neurons into the aDN (e.g., dendritic versus axonic). The exact mechanism of how aDN integrates these different inputs and transforms them into an output that guides egg-laying site selection remains to be examined.

The principal output of the female aDN is the previously undescribed SMP156 neuron, which itself outputs primarily in the IB,

consisting of several single neurons, the number of neurons per cluster is indicated in parenthesis. Neuron types classified as LH-associated, putative PNs, and ascending lALT neurons are color coded. BodyIDs and metadata of neurons in Table S2 are shown.
(E) Confocal light microscopy images of mCD8::GFP-labeled female (top) and male (bottom) aDNs. Boxes indicate the recording regions for calcium imaging.
(F) Mean (dark gray line) and SE (light gray shaded area) of ΔF/F in aDN axonal (top) and dendritic (bottom) compartments in response to a 5-s optogenetic stimulation of olfactory projection neurons (pink bar) in virgin females (left, n = 18), mated females (middle, n = 11), and males (right, n = 10).
(G) Mean and SE of AUC for experiments in (F).
(H) Mean (dark gray) and SE (light gray) of ΔF/F in aDN axonal (top) and dendritic (bottom) compartments in response to a 5-s optogenetic stimulation of olfactory sensory neurons (pink bar) in virgin females (left, n = 14), mated females (middle, n = 10), and males (right, n = 9).
(I) Mean and SE of AUC for experiments in (H).
ns, p.adj > 0.1, •p.adj < 0.1, *p.adj < 0.05, **p.adj < 0.01, ***p.adj < 0.001, and ****p.adj < 0.0001 by Mann-Whitney U-test adjusted for multiple comparisons with the Holm method. See also Figure S5. See Table S3 for the full genotypes.

CellPress
OPEN ACCESS

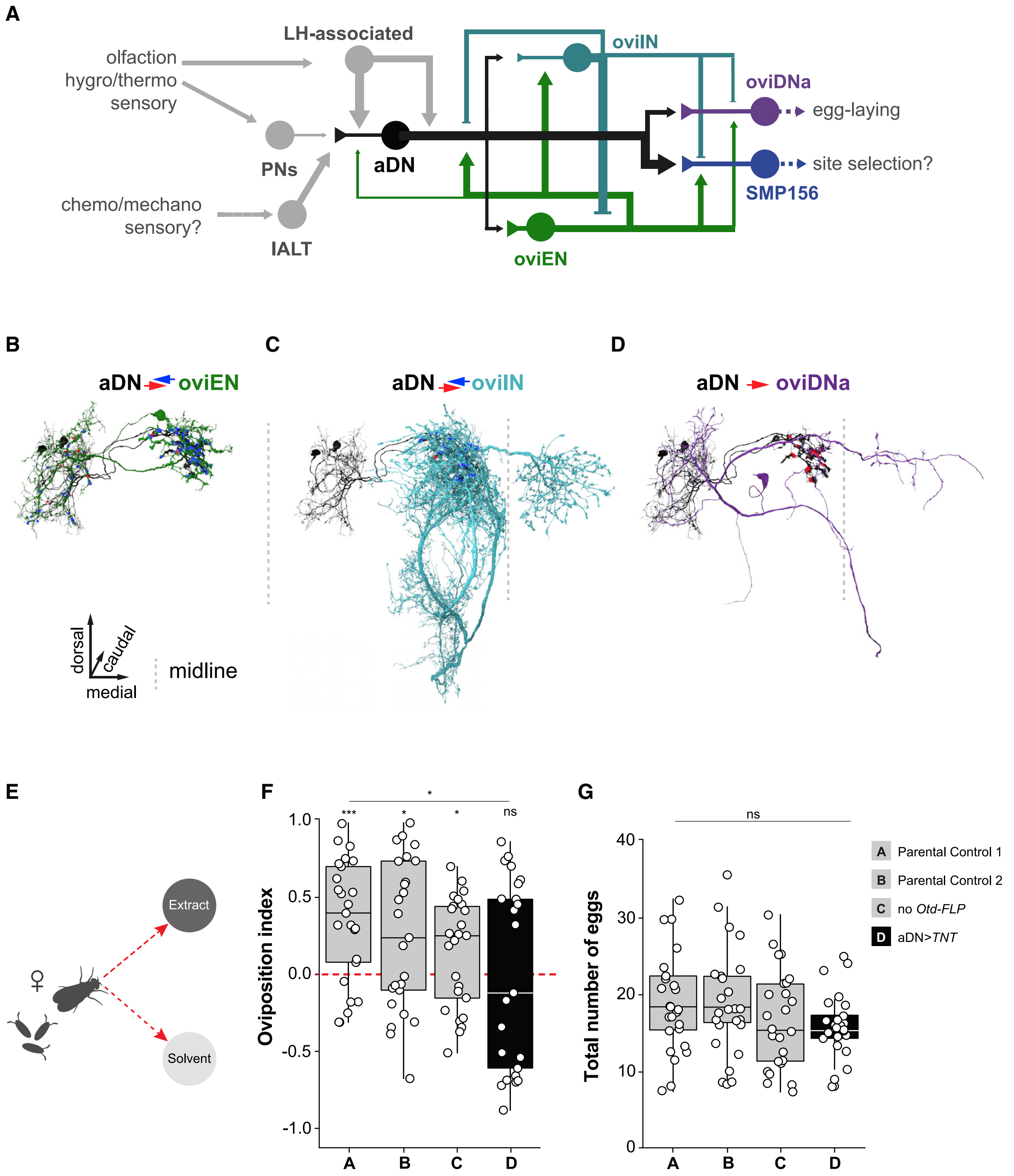

Figure 7. Female aDNs are involved in egg-laying site selection

(A) Wiring diagram depicting the connectivity between aDN (dark gray) and neurons involved in egg laying. The thickness of the lines represents the relative strengths of synaptic connectivity as determined by the number of synaptic connections in the electron microscopy dataset. Inputs from sensory-processing neuropils and the LH into the aDN are shown (light gray).

(B–D) 3D representation of reconstructions from volumetric EM data of aDN (2 neurons in black) and individual egg-laying neurons in the flies' right-brain hemisphere (green and blue) are shown. Input synapses from egg-laying neurons into the aDN are shown in blue and output synapses from aDN into egg-laying neurons are shown in red.

(B) oviEN (type: SMP550; BodyID: 452689494, green) and aDN. aDN receives mainly axo-axonic synapses from oviEN. The aDN provides a small number of inputs into oviEN dendrites.

(C) oviIN (BodyID: 485934965, light blue) and aDN. The ipsilateral oviIN is shown. Synapses between the neuron types are restricted to the axonal filed.

(D) oviDNa (type: SLP410; BodyID: 450971893, purple) and aDN. aDN axonal outputs onto the descending oviDNa restricted to the SMP are shown.

(E) Schematic representation of the two-choice oviposition assay. The oviposition assay contained two 0.75% agar zones with 100 mM of sucrose either containing a pheromone extract (dark gray zone) or a solvent control treatment (light gray zone).

(legend continued on next page)

where its axons show cross-hemisphere connectivity, suggesting it acts as integrators of sensory information from different directions. The major SMP156 output neuron type (IB011) projects to the lobula in the opposite hemisphere, potentially integrating olfactory and visual information as observed in other flying insects during pheromone orientation.[64,65] Olfactory navigation requires comparisons of left and right inputs, e.g., when male moths orient themselves toward conspecific females in response to sex pheromones.[66] Determination of position and direction applies to males pursuing females and females following pheromonal cues to locate a communal egg-laying site. We propose that the aDN cluster in females selectively integrates sensory information, relaying it to SMP156, which confers directionality and processes information relevant to locating an appropriate egg-laying site. In the absence of a male connectome for comparison, we can only speculate about potential shared downstream connectivity. As the male aDN output sites are mainly overlapping with female sites in the SMP (Figures 2J and 2L), it is possible that the male visual pathway also inputs into SMP156, or a similar neuron associated with the IB, potentially feeding back onto visual pathways, supporting appropriate tracking of the female. A male connectome and more genetic tools will help reveal the full extent of downstream functional connectivity and convergence between the sexes.

As fundamental features of most animal species, sexual dimorphisms and sex differences have particular importance for the function of the nervous system. These innate sex-specific adaptations are built during development and orchestrate interactions between sensory information and specific brain regions to shape the phenotype, including the emergent properties of the sex-specific neural circuitry. Evolutionary forces acting on these neural systems have generated adaptive sex differences in behavior.[67] In *Drosophila*, males compete for a mate through courtship displays, while a female's investment is focused on the success of their offspring. These sex-specific behaviors are guided by the perception and processing of sensory cues, ensuring responses lead to reproductive success. In this study, we have shown how a sex-specific switch between visual and olfactory inputs underlies adaptive sex differences in behavior and provides insight on how similar mechanisms may be implemented in the brains of other sexually dimorphic species.

STAR★METHODS

Detailed methods are provided in the online version of this paper and include the following:

- KEY RESOURCES TABLE
- RESOURCE AVAILABILITY
  - Lead contact
  - Materials availability
  - Data and code availability
- EXPERIMENTAL MODEL AND SUBJECT DETAILS
  - Fly strains
- METHOD DETAILS
  - Generation of mosaic clones
  - Immunohistochemistry
  - Confocal image acquisition and processing
  - Representations of anatomical hemibrain data
  - Neuron identification/classification
  - Calcium imaging
  - Courtship assays
  - Automated behavior tracking
  - Female fertility
  - Female egg-laying preference assay
- QUANTIFICATION AND STATISTICAL ANALYSIS
  - Analysis of courtship data
  - Statistical analysis of oviposition
  - Statistical analysis of calcium imaging data

SUPPLEMENTAL INFORMATION

Supplemental Information can be found online at https://doi.org/10.1016/j.cub.2020.12.047.

ACKNOWLEDGMENTS

We thank A. Barrios, A. Dornan, S. Heinze, G. Jefferis, M. Juusola, D. Shepherd, and the Goodwin Lab for helpful discussions and critical reading of the manuscript. We are grateful to D. Anderson, B. Dickson, T. Lee, G. Rubin, D. Yamamoto, and the Bloomington Stock Center (NIH P40OD018537) for sharing important reagents with us. We thank A. de Haan for genotyping and S. Waddell for support with EM reconstruction. N.O. was supported by a Wellcome Trust Collaborative Award (203261/Z/16/Z) to S. Waddell. This work was supported by grants from the BBSRC (BB/T001348/1) to T.N. and S.F.G. and a Wellcome Trust Senior Investigator Award to S.F.G. (106189/Z/14/Z).

AUTHOR CONTRIBUTIONS

Conceptualization, T.N., A.R., A.M.A., M.C.N., and S.F.G.; methodology, T.N., A.R., A.M.A., N.O., T.A.V., M.C.N., J.-C.B., and S.F.G.; investigation, T.N., A.R., A.M.A., N.O., T.A.V., and M.C.N.; resources, S.F.G.; writing, T.N., A.R., A.M.A., N.O., T.A.V., J.-C.B., M.C.N., and S.F.G.; supervision, M.C.N. and S.F.G.; funding acquisition, S.F.G.

DECLARATION OF INTERESTS

The authors declare no competing interests.

(F) The oviposition preference to the pheromone extracts. A preference for the pheromone extract would result in an oviposition index of 1.0 and an aversion would result in an oviposition index of −1.0. The significance of the preferences being different from 0 ("no preference") are indicated above each boxplot as determined by a two-tailed Wilcoxon signed rank tests. The difference in preference across the genotypes was tested with a generalized linear model (GLM) with quasibinomial error distribution and is indicated above the bar (*$p < 0.05$; ***$p < 0.001$; ns, $p > 0.05$).
(G) The total number of eggs laid by the different genotypes in the oviposition assay. Difference across genotypes was tested with a GLM with negative binomial error distribution (ns, $p > 0.05$).
(F and G) For boxplots, boxes represent 1st to 3rd quartile, bar represents the median, lower whisker represents the smallest value at most 1.5 * inter-quartile range from the 1st quartile, upper whisker represents the largest value at most 1.5 * inter-quartile range from the 3rd quartile. Points represent all individual data points. Full genotypes: (A) *Otd-FLP/+; dsx$^{Gal4.DBD}$/+* (n = 25); (B) *VGlut$^{dVP16.AD}$, UAS > stop > TNT/+* (n = 25); (C) *VGlut$^{dVP16.AD}$, UAS > stop > TNT/+; dsx$^{Gal4.DBD}$/+* (n = 25); (D) *VGlut$^{dVP16.AD}$, UAS > stop > TNT/Otd-FLP; dsx$^{Gal4.DBD}$/+* (n = 25). See also Figure S6. See Table S3 for the full genotypes.

Received: November 12, 2020
Revised: December 15, 2020
Accepted: December 24, 2020
Published: January 27, 2021

REFERENCES

1. Yang, T., and Shah, N.M. (2016). Molecular and neural control of sexually dimorphic social behaviors. Curr. Opin. Neurobiol. *38*, 89–95.
2. Luo, S.D., Shi, G.W., and Baker, B.S. (2011). Direct targets of the *D. melanogaster* DSX[F] protein and the evolution of sexual development. Development *138*, 2761–2771.
3. Dalton, J.E., Fear, J.M., Knott, S., Baker, B.S., McIntyre, L.M., and Arbeitman, M.N. (2013). Male-specific Fruitless isoforms have different regulatory roles conferred by distinct zinc finger DNA binding domains. BMC Genomics *14*, 659.
4. Neville, M.C., Nojima, T., Ashley, E., Parker, D.J., Walker, J., Southall, T., Van de Sande, B., Marques, A.C., Fischer, B., Brand, A.H., et al. (2014). Male-specific fruitless isoforms target neurodevelopmental genes to specify a sexually dimorphic nervous system. Curr. Biol. *24*, 229–241.
5. Vernes, S.C. (2014). Genome wide identification of *fruitless* targets suggests a role in upregulating genes important for neural circuit formation. Sci. Rep. *4*, 4412.
6. Clough, E., Jimenez, E., Kim, Y.A., Whitworth, C., Neville, M.C., Hempel, L.U., Pavlou, H.J., Chen, Z.X., Sturgill, D., Dale, R.K., et al. (2014). Sex- and tissue-specific functions of *Drosophila doublesex* transcription factor target genes. Dev. Cell *31*, 761–773.
7. Asahina, K. (2018). Sex differences in *Drosophila* behavior: qualitative and quantitative dimorphism. Curr. Opin. Physiol. *6*, 35–45.
8. Verhulst, E.C., and van de Zande, L. (2015). Double nexus–Doublesex is the connecting element in sex determination. Brief. Funct. Genomics *14*, 396–406.
9. Clough, E., and Oliver, B. (2012). Genomics of sex determination in Drosophila. Brief. Funct. Genomics *11*, 387–394.
10. Baker, B.S., and Wolfner, M.F. (1988). A molecular analysis of *doublesex*, a bifunctional gene that controls both male and female sexual differentiation in *Drosophila melanogaster*. Genes Dev. *2*, 477–489.
11. Burtis, K.C., and Baker, B.S. (1989). Drosophila *doublesex* gene controls somatic sexual differentiation by producing alternatively spliced mRNAs encoding related sex-specific polypeptides. Cell *56*, 997–1010.
12. Lee, G., Hall, J.C., and Park, J.H. (2002). *Doublesex* gene expression in the central nervous system of *Drosophila melanogaster*. J. Neurogenet. *16*, 229–248.
13. Billeter, J.C., Villella, A., Allendorfer, J.B., Dornan, A.J., Richardson, M., Gailey, D.A., and Goodwin, S.F. (2006). Isoform-specific control of male neuronal differentiation and behavior in *Drosophila* by the *fruitless* gene. Curr. Biol. *16*, 1063–1076.
14. Rideout, E.J., Dornan, A.J., Neville, M.C., Eadie, S., and Goodwin, S.F. (2010). Control of sexual differentiation and behavior by the *doublesex* gene in *Drosophila melanogaster*. Nat. Neurosci. *13*, 458–466.
15. Sanders, L.E., and Arbeitman, M.N. (2008). Doublesex establishes sexual dimorphism in the *Drosophila* central nervous system in an isoform-dependent manner by directing cell number. Dev. Biol. *320*, 378–390.
16. Rideout, E.J., Billeter, J.C., and Goodwin, S.F. (2007). The sex-determination genes *fruitless* and *doublesex* specify a neural substrate required for courtship song. Curr. Biol. *17*, 1473–1478.
17. Robinett, C.C., Vaughan, A.G., Knapp, J.M., and Baker, B.S. (2010). Sex and the single cell. II. There is a time and place for sex. PLoS Biol. *8*, e1000365.
18. Kimura, K., Sato, C., Koganezawa, M., and Yamamoto, D. (2015). *Drosophila* ovipositor extension in mating behavior and egg deposition involves distinct sets of brain interneurons. PLoS ONE *10*, e0126445.
19. Lee, T., and Luo, L. (1999). Mosaic analysis with a repressible cell marker for studies of gene function in neuronal morphogenesis. Neuron *22*, 451–461.
20. Luan, H., Peabody, N.C., Vinson, C.R., and White, B.H. (2006). Refined spatial manipulation of neuronal function by combinatorial restriction of transgene expression. Neuron *52*, 425–436.
21. Cachero, S., Ostrovsky, A.D., Yu, J.Y., Dickson, B.J., and Jefferis, G.S. (2010). Sexual dimorphism in the fly brain. Curr. Biol. *20*, 1589–1601.
22. Wu, M., Nern, A., Williamson, W.R., Morimoto, M.M., Reiser, M.B., Card, G.M., and Rubin, G.M. (2016). Visual projection neurons in the *Drosophila* lobula link feature detection to distinct behavioral programs. eLife *5*, e21022.
23. Panser, K., Tirian, L., Schulze, F., Villalba, S., Jefferis, G.S.X.E., Bühler, K., and Straw, A.D. (2016). Automatic segmentation of Drosophila neural compartments using GAL4 expression data reveals novel visual pathways. Curr. Biol. *26*, 1943–1954.
24. Omoto, J.J., Keleş, M.F., Nguyen, B.M., Bolanos, C., Lovick, J.K., Frye, M.A., and Hartenstein, V. (2017). Visual input to the Drosophila central complex by developmentally and functionally distinct neuronal populations. Curr. Biol. *27*, 1098–1110.
25. Ribeiro, I.M.A., Drews, M., Bahl, A., Machacek, C., Borst, A., and Dickson, B.J. (2018). Visual projection neurons mediating directed courtship in *Drosophila*. Cell *174*, 607–621.e18.
26. Lamaze, A., Krätschmer, P., Chen, K.F., Lowe, S., and Jepson, J.E.C. (2018). A wake-promoting circadian output circuit in *Drosophila*. Curr. Biol. *28*, 3098–3105.e3.
27. Ruta, V., Datta, S.R., Vasconcelos, M.L., Freeland, J., Looger, L.L., and Axel, R. (2010). A dimorphic pheromone circuit in *Drosophila* from sensory input to descending output. Nature *468*, 686–690.
28. Kohl, J., Ostrovsky, A.D., Frechter, S., and Jefferis, G.S. (2013). A bidirectional circuit switch reroutes pheromone signals in male and female brains. Cell *155*, 1610–1623.
29. Clowney, E.J., Iguchi, S., Bussell, J.J., Scheer, E., and Ruta, V. (2015). Multimodal chemosensory circuits controlling male courtship in *Drosophila*. Neuron *87*, 1036–1049.
30. Kallman, B.R., Kim, H., and Scott, K. (2015). Excitation and inhibition onto central courtship neurons biases *Drosophila* mate choice. eLife *4*, e11188.
31. Kim, H., Kirkhart, C., and Scott, K. (2017). Long-range projection neurons in the taste circuit of *Drosophila*. eLife *6*, e23386.
32. Tsubouchi, A., Yano, T., Yokoyama, T.K., Murtin, C., Otsuna, H., and Ito, K. (2017). Topological and modality-specific representation of somatosensory information in the fly brain. Science *358*, 615–623.
33. Dolan, M.J., Belliart-Guérin, G., Bates, A.S., Frechter, S., Lampin-Saint-Amaux, A., Aso, Y., Roberts, R.J.V., Schlegel, P., Wong, A., Hammad, A., et al. (2018). Communication from learned to innate olfactory processing centers is required for memory retrieval in *Drosophila*. Neuron *100*, 651–668.e8.
34. Pavlou, H.J., Lin, A.C., Neville, M.C., Nojima, T., Diao, F., Chen, B.E., White, B.H., and Goodwin, S.F. (2016). Neural circuitry coordinating male copulation. eLife *5*, e20713.
35. Nicolaï, L.J., Ramaekers, A., Raemaekers, T., Drozdzecki, A., Mauss, A.S., Yan, J., Landgraf, M., Annaert, W., and Hassan, B.A. (2010). Genetically encoded dendritic marker sheds light on neuronal connectivity in Drosophila. Proc. Natl. Acad. Sci. USA *107*, 20553–20558.
36. Estes, P.S., Ho, G.L., Narayanan, R., and Ramaswami, M. (2000). Synaptic localization and restricted diffusion of a *Drosophila* neuronal synaptobrevin–green fluorescent protein chimera in vivo. J. Neurogenet. *13*, 233–255.
37. Gordon, M.D., and Scott, K. (2009). Motor control in a *Drosophila* taste circuit. Neuron *61*, 373–384.
38. Scheffer, L.K., Xu, C.S., Januszewski, M., Lu, Z., Takemura, S.Y., Hayworth, K.J., Huang, G.B., Shinomiya, K., Maitlin-Shepard, J., Berg,

S., et al. (2020). A connectome and analysis of the adult *Drosophila* central brain. eLife *9*, e57443.

39. Klapoetke, N.C., Murata, Y., Kim, S.S., Pulver, S.R., Birdsey-Benson, A., Cho, Y.K., Morimoto, T.K., Chuong, A.S., Carpenter, E.J., Tian, Z., et al. (2014). Independent optical excitation of distinct neural populations. Nat. Methods *11*, 338–346.
40. Chen, T.W., Wardill, T.J., Sun, Y., Pulver, S.R., Renninger, S.L., Baohan, A., Schreiter, E.R., Kerr, R.A., Orger, M.B., Jayaraman, V., et al. (2013). Ultrasensitive fluorescent proteins for imaging neuronal activity. Nature *499*, 295–300.
41. Zhang, S.X., Miner, L.E., Boutros, C.L., Rogulja, D., and Crickmore, M.A. (2018). Motivation, perception, and chance converge to make a binary decision. Neuron *99*, 376–388.e6.
42. Seeholzer, L.F., Seppo, M., Stern, D.L., and Ruta, V. (2018). Evolution of a central neural circuit underlies *Drosophila* mate preferences. Nature *559*, 564–569.
43. Hamasaka, Y., Wegener, C., and Nässel, D.R. (2005). GABA modulates *Drosophila* circadian clock neurons via GABAB receptors and decreases in calcium. J. Neurobiol. *65*, 225–240.
44. Sweeney, S.T., Broadie, K., Keane, J., Niemann, H., and O'Kane, C.J. (1995). Targeted expression of tetanus toxin light chain in *Drosophila* specifically eliminates synaptic transmission and causes behavioral defects. Neuron *14*, 341–351.
45. Asahina, K., Watanabe, K., Duistermars, B.J., Hoopfer, E., González, C.R., Eyjólfsdóttir, E.A., Perona, P., and Anderson, D.J. (2014). Tachykinin-expressing neurons control male-specific aggressive arousal in *Drosophila*. Cell *156*, 221–235.
46. von Philipsborn, A.C., Liu, T., Yu, J.Y., Masser, C., Bidaye, S.S., and Dickson, B.J. (2011). Neuronal control of *Drosophila* courtship song. Neuron *69*, 509–522.
47. Pan, Y., Meissner, G.W., and Baker, B.S. (2012). Joint control of *Drosophila* male courtship behavior by motion cues and activation of male-specific P1 neurons. Proc. Natl. Acad. Sci. USA *109*, 10065–10070.
48. Kimura, K., Hachiya, T., Koganezawa, M., Tazawa, T., and Yamamoto, D. (2008). Fruitless and doublesex coordinate to generate male-specific neurons that can initiate courtship. Neuron *59*, 759–769.
49. Yu, J.Y., Kanai, M.I., Demir, E., Jefferis, G.S., and Dickson, B.J. (2010). Cellular organization of the neural circuit that drives *Drosophila* courtship behavior. Curr. Biol. *20*, 1602–1614.
50. Kohatsu, S., Koganezawa, M., and Yamamoto, D. (2011). Female contact activates male-specific interneurons that trigger stereotypic courtship behavior in *Drosophila*. Neuron *69*, 498–508.
51. Heimbeck, G., Bugnon, V., Gendre, N., Keller, A., and Stocker, R.F. (2001). A central neural circuit for experience-independent olfactory and courtship behavior in *Drosophila melanogaster*. Proc. Natl. Acad. Sci. USA *98*, 15336–15341.
52. Marin, E.C., Büld, L., Theiss, M., Sarkissian, T., Roberts, R.J.V., Turnbull, R., Tamimi, I.F.M., Pleijzier, M.W., Laursen, W.J., Drummond, N., et al. (2020). Connectomics analysis reveals first-, second-, and third-order thermosensory and hygrosensory neurons in the adult *Drosophila* brain. Curr. Biol. *30*, 3167–3182.e4.
53. Stocker, R.F., Heimbeck, G., Gendre, N., and de Belle, J.S. (1997). Neuroblast ablation in *Drosophila* P[GAL4] lines reveals origins of olfactory interneurons. J. Neurobiol. *32*, 443–456.
54. Lai, S.L., Awasaki, T., Ito, K., and Lee, T. (2008). Clonal analysis of *Drosophila* antennal lobe neurons: diverse neuronal architectures in the lateral neuroblast lineage. Development *135*, 2883–2893.
55. Lai, S.L., and Lee, T. (2006). Genetic mosaic with dual binary transcriptional systems in Drosophila. Nat. Neurosci. *9*, 703–709.
56. Wang, F., Wang, K., Forknall, N., Patrick, C., Yang, T., Parekh, R., Bock, D., and Dickson, B.J. (2020). Neural circuitry linking mating and egg laying in *Drosophila* females. Nature *579*, 101–105.
57. Liu, W.W., and Wilson, R.I. (2013). Glutamate is an inhibitory neurotransmitter in the *Drosophila* olfactory system. Proc. Natl. Acad. Sci. USA *110*, 10294–10299.
58. Felsenberg, J., Jacob, P.F., Walker, T., Barnstedt, O., Edmondson-Stait, A.J., Pleijzier, M.W., Otto, N., Schlegel, P., Sharifi, N., Perisse, E., et al. (2018). Integration of parallel opposing memories underlies memory extinction. Cell *175*, 709–722.e15.
59. Shih, C.T., Sporns, O., Yuan, S.L., Su, T.S., Lin, Y.J., Chuang, C.C., Wang, T.Y., Lo, C.C., Greenspan, R.J., and Chiang, A.S. (2015). Connectomics-based analysis of information flow in the *Drosophila* brain. Curr. Biol. *25*, 1249–1258.
60. Duménil, C., Woud, D., Pinto, F., Alkema, J.T., Jansen, I., Van Der Geest, A.M., Roessingh, S., and Billeter, J.C. (2016). Pheromonal cues deposited by mated females convey social information about egg-laying sites in *Drosophila melanogaster*. J. Chem. Ecol. *42*, 259–269.
61. Cury, K.M., Prud'homme, B., and Gompel, N. (2019). A short guide to insect oviposition: when, where and how to lay an egg. J. Neurogenet. *33*, 75–89.
62. Billeter, J.C., and Wolfner, M.F. (2018). Chemical cues that guide female reproduction in *Drosophila melanogaster*. J. Chem. Ecol. *44*, 750–769.
63. Cattaert, D., and El Manira, A. (1999). Shunting versus inactivation: analysis of presynaptic inhibitory mechanisms in primary afferents of the crayfish. J. Neurosci. *19*, 6079–6089.
64. Verspui, R., and Gray, J.R. (2009). Visual stimuli induced by self-motion and object-motion modify odour-guided flight of male moths (*Manduca sexta* L.). J. Exp. Biol. *212*, 3272–3282.
65. Pansopha, P., Ando, N., and Kanzaki, R. (2014). Dynamic use of optic flow during pheromone tracking by the male silkmoth, *Bombyx mori*. J. Exp. Biol. *217*, 1811–1820.
66. Hildebrand, J.G. (1996). Olfactory control of behavior in moths: central processing of odor information and the functional significance of olfactory glomeruli. J. Comp. Physiol. A Neuroethol. Sens. Neural Behav. Physiol. *178*, 5–19.
67. Tosches, M.A. (2017). Developmental and genetic mechanisms of neural circuit evolution. Dev. Biol. *431*, 16–25.
68. Gao, S., Takemura, S.Y., Ting, C.Y., Huang, S., Lu, Z., Luan, H., Rister, J., Thum, A.S., Yang, M., Hong, S.T., et al. (2008). The neural substrate of spectral preference in *Drosophila*. Neuron *60*, 328–342.
69. Eyjolfsdottir, E., Branson, S., Burgos-Artizzu, X., Hoopfer, E.D., Schor, J., Anderson, D.J., and Perona, P. (2014). Detecting social actions of fruit flies. In Computer Vision – ECCV 2014, 8690, D. Fleet, T. Pajdla, B. Schiele, and T. Tuytelaars, eds. (Springer, Cham), pp. 772–787. https://doi.org/10.1007/978-3-319-10605-2_50.
70. Kabra, M., Robie, A.A., Rivera-Alba, M., Branson, S., and Branson, K. (2013). JAABA: interactive machine learning for automatic annotation of animal behavior. Nat. Methods *10*, 64–67.
71. Jenett, A., Gerald, R.M., Ngo, T.-T.B., Shepherd, D., Murphy, C., Dionne, H., et al. (2012). A GAL4-Driver Line Resource for Drosophila Neurobiology. Cell Reports *2*, 991–1001.
72. Ito, K., Shinomiya, K., Ito, M., Armstrong, J.D., Boyan, G., Hartenstein, V., et al. (2014). A Systematic Nomenclature for the Insect Brain. Neuron *81*, 755–765.
73. Bates, A.S., Schlegel, P., Roberts, R.J.V., Drummond, N., Tamimi, I.F.M., Turnbull, R., Zhao, X., Marin, E.C., Popovici, P.D., Dhawan, S., et al. (2020). Complete connectomic reconstruction of olfactory projection neurons in the fly brain. Curr. Biol. *30*, 3183–3199.e6.
74. Frechter, S., Bates, A.S., Tootoonian, S., Dolan, M.J., Manton, J., Jamasb, A.R., Kohl, J., Bock, D., and Jefferis, G. (2019). Functional and anatomical specificity in a higher olfactory centre. eLife *8*, e44590.
75. Pologruto, T.A., Sabatini, B.L., and Svoboda, K. (2003). ScanImage: flexible software for operating laser scanning microscopes. Biomed. Eng. Online *2*, 13.

76. Wickham, H. (2016). ggplot2: Elegant Graphics for Data Analysis (Springer).

77. Therneau, T.M., and Grambsch, P.M. (2000). Modeling Survival Data: Extending the Cox Model (Springer-Verlag).

78. Kassambara, A., Kosinski, M., and Biecek, P. (2019). Survminer: drawing survival curves using 'ggplot2'. https://cran.r-project.org/web/packages/survminer/index.html.

79. Marsaglia, G., Tsang, W.W., and Wang, J. (2003). Evaluating Kolmogorov's distribution. J. Stat. Softw. *8*, 1–4.

80. Bates, D., Mächler, M., Bolker, B., and Walker, S. (2015). Fitting linear mixed-effects models using lme4. J. Stat. Softw. *67*, 1–48.

81. Fox, J., and Weisber, S. (2011). An R Companion to Applied Regression (Sage Publications).

82. Hothorn, T., Bretz, F., and Westfall, P. (2008). Simultaneous inference in general parametric models. Biom. J. *50*, 346–363.

STAR★METHODS

KEY RESOURCES TABLE

REAGENT or RESOURCE	SOURCE	IDENTIFIER
Antibodies		
Anti-GFP Polyclonal (rabbit)	Thermo Fisher Scientific	A6455; RRID: AB_221570
Anti-GFP Monoclonal (mouse)	Sigma-Aldrich	G6539; RRID: AB_259941
Anti-mCherry Monoclonal 16D7 (rat)	Thermo Fisher Scientific	M11217; RRID: AB_2536611
Anti-Brp (nc82) Monoclonal (mouse)	Developmental Studies Hybridoma Bank (University of Iowa)	RRID: AB_2314866
Anti-CadN DN-Ex #8 (mouse)	Developmental Studies Hybridoma Bank (University of Iowa)	RRID: AB_528121
Goat Anti-rabbit Alexa 488	Thermo Fisher Scientific	A11034; RRID: AB_2576217
Goat Anti-rat Alexa 546	Thermo Fisher Scientific	A11081; RRID: AB_2534125
Goat Anti-rat Alexa 633	Thermo Fisher Scientific	A21094; RRID: AB_2535749
Goat anti-mouse Alexa 488	Thermo Fisher Scientific	A28175; RRID: AB_2536161
Goat Anti-mouse Alexa 546	Thermo Fisher Scientific	A11030; RRID: AB_2534089
Goat Anti-mouse Alexa 633	Thermo Fisher Scientific	A21050; RRID: AB_2535718
Chemicals, peptides, and recombinant proteins		
N-hexane	Fisher Scientific	CAS Number-110-54-3
BD Difco	Fisher Scientific	CAS Number-9002-18-0
Yeast extract powder	Fisher Scientific	CAS Number-8013-01-02
Formaldehyde	Sigma-Aldrich	Cat# 47608-250ML-F
Normal Goat Serum	Sigma-Aldrich	Cat# G9023
Phosphate buffered saline (PBS)	Sigma-Aldrich	Cat# P3183-10PAK
Triton X-100	Sigma-Aldrich	Cat# T8787-100ML
All-trans retinal	Sigma-Aldrich	Cat# R2500
NaCl	Sigma-Aldrich	Cat# S7653
KCl	Sigma-Aldrich	Cat# P9333
$NaHCO_3$	Sigma-Aldrich	Cat# S6297
NaH_2PO_4	Sigma-Aldrich	Cat# S8282
$CaCl_2$	Sigma-Aldrich	Cat# 21115
$MgCl_2$	Sigma-Aldrich	Cat# M1028
N-Tris	Sigma-Aldrich	Cat# T5691
Trehalose	Sigma-Aldrich	Cat# T9531
Glucose	Sigma-Aldrich	Cat# G7528
Sucrose	Sigma-Aldrich	Cat# S0389
Vectashield mounting medium	Vector Laboratories	Cat# H-1000
Deposited data		
doublesex^{+} clones	This study	https://v2.virtualflybrain.org
Experimental models: organisms/strains		
Drosophila: wild-type Canton-S	Gift from Jeffrey Hall	N/A
Drosophila: dsx$^{Gal4.DBD}$	Pavlou et al.[34]	N/A
Drosophila: dsx^{Gal4}	Rideout et al.[16]	N/A
Drosophila: VGlut$^{dVP16.AD}$	Gao et al.[68]	N/A
Drosophila: VT029314-LexA	Ribeiro et al.[25]	N/A
Drosophila: R35D04-p65.AD; R22D06-Gal4.DBD	Bloomington DSC	RRID: BDSC_68336
Drosophila: fru^{NP21}	Bloomington DSC	RRID: BDSC_30027

(*Continued on next page*)

Continued

REAGENT or RESOURCE	SOURCE	IDENTIFIER
Drosophila: GMR22D04-Gal4	Bloomington DSC	RRID: BDSC_48981
Drosophila: fru^{FLP}	Bloomington DSC	RRID: BDSC_66870
Drosophila: UAS > mCherry > ReaChR	Bloomington DSC	RRID: BDSC_53740
Drosophila: 10xUAS-IVS-mCD8::GFP	Bloomington DSC	RRID: BDSC_32185
Drosophila: 40xUAS-IVS-mCD8::GFP	Bloomington DSC	RRID: BDSC_32195
Drosophila: UAS-DenMark, UAS-Syb::EGFP	Bloomington DSC	RRID: BDSC_33064
Drosophila: UAS-CD4::spGFP1-10, lexAop-CD4::spGFP11	Bloomington DSC	RRID: BDSC_58755
Drosophila: UAS-GCaMP6f	Bloomington DSC	RRID: BDSC_42747
Drosophila: 13xlexAop2-IVS-myr::GFP	Bloomington DSC	RRID: BDSC_32210
Drosophila: 13xLexAop2-IVS-CsChrimson::tdTomato	Bloomington DSC	RRID: BDSC_82183
Drosophila: UAS > stop > TNT	Bloomington DSC	RRID: BDSC_67690
Drosophila: GH146-LexA	Gift from Tzumin Lee	N/A
Drosophila: Orco-LexA::VP16	Gift from Tzumin Lee	N/A
Drosophila: Trh-p65.AD	Gift from Gerald Rubin	N/A
Drosophila: hs-FLP(22); FRTG13, UAS-mCD8::GFP	Gift from Daisuke Yamamoto	N/A
Drosophila: hs-FLP(22); FRTG13, tubP-Gal80	Gift from Daisuke Yamamoto	N/A
Drosophila: Otd-FLP	Gift from David Anderson	N/A
Drosophila: UAS > stop > mCD8::GFP	Gift from Barry Dickson	N/A
Software and algorithms		
Fiji	open source	https://fiji.sc
Image stabilizer	From Kang Li	http://www.cs.cmu.edu/~kangli/code/Image_Stabilizer.html
CMTK Registration Toolkit	From Gregory Jefferis	https://github.com/jefferis/fiji-cmtk-gui
Neuroglancer Hub	Google; Janelia EM; Janelia Scientific Computing	https://neuroglancerhub.github.io/
neuPRINT	Janelia EM	https://neuprint.janelia.org/
Blender v2.8.2	Blender	https://www.blender.org/download/releases/2-82/
navis v2.2.-blender interface	Python Software Foundation GitHub	https://pypi.org/project/navis/ https://github.com/schlegelp/navis
FlyLight	HHMI Janelia	http://splitgal4.janelia.org/cgi-bin/splitgal4.cgi
NeuronBridge	HHMI Janelia	https://neuronbridge.janelia.org
Amira 5.4.2	Thermo Fisher Scientific	RRID: SCR_007353
Caltech Fly Tracker	Eyjolfsdottir et al.[69]	http://www.vision.caltech.edu/Tools/FlyTracker/
JAABA	Kabra et al.[70]	http://jaaba.sourceforge.net/
GraphPad Prism 8	GraphPad Software, La Jolla, CA	https://www.graphpad.com/scientific-software/prism/
ImageJ	open source	https://imagej.nih.gov/ij/
Adobe Illustrator CC	Adobe Systems, San Jose, CA	https://www.adobe.com/uk/products/illustrator.html
MATLAB	The Mathworks, Natick, MA	https://uk.mathworks.com/products/matlab.html
LabView	National Instruments	https://www.ni.com/en-gb/shop/labview.html

(*Continued on next page*)

Continued		
REAGENT or RESOURCE	SOURCE	IDENTIFIER
any2ufmf	open source	http://ctrax.sourceforge.net/any2ufmf.html
R	R Development Core Team, 2020	https://www.r-project.org/
Calcium imaging analysis scripts	This study	https://github.com/AR2202/2-photon
Python (scipy and statsmodels)	Python Software Foundation	https://www.python.org/
Behavioral analysis scripts	This study	https://github.com/aaron-allen/aDN_behaviour and https://github.com/aaron-allen/goodwin-lab-tracking

RESOURCE AVAILABILITY

Lead contact

Further information and requests for resources and reagents should be directed to and will be fulfilled by the Lead Contact, Stephen F. Goodwin (stephen.goodwin@cncb.ox.ac.uk).

Materials availability

This study did not generate new unique reagents.

Data and code availability

Scripts used for behavioral analysis are available from https://github.com/aaron-allen/aDN_behaviour and https://github.com/aaron-allen/goodwin-lab-tracking and those for calcium imaging analysis from https://github.com/AR2202/2-photon. Single neuron and cluster images described in this study will be uploaded to a publicly available database hosted by Virtual Fly Brain (https://v2.virtualflybrain.org) following publication. Requests for further details of the software and raw data should be directed to and will be fulfilled by the Lead Contact, Stephen F. Goodwin (stephen.goodwin@cncb.ox.ac.uk).

EXPERIMENTAL MODEL AND SUBJECT DETAILS

Fly strains

All *Drosophila melanogaster* strains were reared at 25°C and 40%–50% humidity on standard cornmeal-agar food in 12:12 h light: dark cycle. Flies were aged 3-8 days post eclosion. Sexes of the flies used are stated in each Figure and Legend. Flies used for optogenetic activation experiments were transferred to food containing 1 mM all-*trans* retinal during adulthood[39]. For GRASP experiments, flies were reared at 18°C and aged 14-20 days post eclosion[37]. Fly strains used in this study (the full genotype list is available in Table S3) include wild-type *Canton-S*; *dsx^{Gal4}* ; *hs-FLP(22), FRTG13, UAS-mCD8::GFP* and *FRTG13, tubP-Gal80* (Daisuke Yamamoto); *dsx$^{Gal4.DBD}$*; *VGlut$^{dVP16.AD}$*; *VT029314-LexA*; *R35D04-p65.AD* and *R22D06-Gal4.DBD*; *R22D04-Gal4* (BDSC #48981); *GH146-LexA* (Tzumin Lee); *Orco-LexA* (Tzumin Lee); *Otd-FLP* (David Anderson); *UAS > mCherry > ReaChR* (BDSC #53743); *10xUAS-IVS-mCD8::GFP* (BDSC #32186); *UAS > stop > mCD8::GFP* (Barry Dickson); *UAS-DenMark*; *UAS-Syb::EGFP* ; *UAS-CD4::spGFP1-10, lexAop-CD4::spGFP11*; *UAS-GCaMP6f* ; *13XlexAop2-CsChrimson::tdTom)* (BDSC #82138), and *UAS > stop > TNT*. See Table S3 for the concrete genotypes of the flies used in each experiment.

METHOD DETAILS

Generation of mosaic clones

Somatic clones were produced using the MARCM method as described previously[19]. The flies used for MARCM analysis were obtained from crosses between *y, w, hs-FLP(22); FRTG13, UAS-mCD8::GFP; dsx^{Gal4}* and *y, w, hs-FLP(22); FRTG13, tubP-Gal80; UAS-mCD8::GFP*. To generate mosaic clones, chromosomal recombination was induced by applying three heat-shock treatments to embryos and larvae 24, 36 and 48 h after egg-laying at 38 °C each for 10-15 min. In another set of MARCM experiments, the flies were obtained from crosses between *y, hs-flp; FRTG13, UAS-mCD8::GFP; fru^{NP21}* and *y, w, hs-FLP(22); FRTG13, tubP-Gal80; UAS-mCD8::GFP*. Heat-shock was applied to embryos 24 h after egg laying at 38°C for 7 min.

Immunohistochemistry

After a brief pre-wash of adult flies in 100% EtOH to remove hydrophobic cuticular chemical compounds, brains and VNCs were dissected in PBS at RT (20-25°C), collected in 2 mL sample tubes and fixed with 4% formaldehyde (Sigma-Aldrich) in PBS (Sigma-Aldrich) for 20 min at RT. After fixation, tissues were washed in 0.7% PBS/Triton X-100 (Sigma-Aldrich) (PBT) 3 times each for 20 min at RT. After blocking in 10% normal goat serum (Sigma-Aldrich) in PBT (NGS/PBT) overnight (8-12 h) at RT, tissues were incubated in primary antibody solutions for 72 h at 4°C (1:1000, rabbit anti-GFP, Thermo Fisher Scientific; 1:100, mouse anti-GFP, Sigma-Aldrich; 1:1000, rat

anti-mCherry, Thermo Fisher Scientific; 1:10, mouse anti-Brp, Developmental Studies Hybridoma Bank; 1:50, rat anti-CadN, Developmental Studies Hybridoma Bank). After 4 washes in PBT for 1 h each at RT, tissues were incubated in secondary antibody solutions for 48 h at 4°C (1:500, anti-rabbit Alexa Fluor 488, anti-mouse Alexa Fluor 488, anti-mouse Alexa Fluor 546, anti-rat Alexa Fluor 546, anti-rat Alexa Fluor 633, anti-mouse Alexa Fluor 546, anti-mouse Alexa Fluor 633, Thermo Fisher Scientific). After 4 washes in PBT for 1 h each at RT, 70% glycerol in PBS was added to the sample tubes, which were subsequently transferred to −20°C and kept for at least 8 hr for tissue clearing. Specimens were mounted in Vectashield (Vector Laboratories).

Confocal image acquisition and processing

Confocal image stacks were acquired on a Leica TCS SP5 confocal microscope at 1024 × 1024-pixel resolution with a slice size of 1 μm. Water-immersion 25x and oil-immersion 40x objective lenses were used for VNC and brain images, respectively. Z stack images were generated, and signals in unrelated regions, background noise and unexpected tissue debris were erased using Fiji (https://fiji.sc/). Images were registered on to intersex template brain and VNC using the Fiji Computational Morphometry Toolkit (CMTK) Registration GUI (https://github.com/jefferis/fiji-cmtk-gui;)[21]. 3D volume-rendered images were generated using Amira 5.4.2 (Thermo Fisher Scientific; https://www.thermofisher.com/amira-avizo). The schematic drawings of the AOTu and SLP (Figures 2D and 2H) are made by modifying the JFRC2 template brain and neuropil labels downloaded from the Virtual Fly Brain database.[71,72]

Representations of anatomical hemibrain data

Volumetric data of neurons and neuropils was obtained from the hemibrain project's neuPrint neuroglancer plugin (HHMI Janelia Fly EM; GoogleAI; https://neuroglancerhub.github.io/ ; https://neuprint.janelia.org/)[38]. Blender v2.8.2 with navis v2.2.-blender interface (https://pypi.org/project/navis/ ; https://github.com/schlegelp/navis)[73] and custom python-based scripts have been used to assemble and render anatomical 3D representations. Connectivity data and synapse locations were obtained from the neuPrint database and have been processed with navis v2.2 based custom python scripts inside the blender python 3.7 console. All scripts are available upon request.

Neuron identification/classification

Neuron clusters are based on neuPrint v1.1 except for oviDNa2, oviEN and lALT1-lALT5 clusters which are described here.

aDN (541347811, 604070433), oviDNa (550655668), and oviIN (485934965) were identified previously and are annotated on neuPrint. We have compared their morphologies with our light microscopic images in case of aDN, and driver line stacks published previously[56] and hosted on FlyLight (http://splitgal4.janelia.org/cgi-bin/splitgal4.cgi) for the neurons described below.

To identify oviEN and oviDNa2 neurons, we assessed connectivity data of oviDNa, oviIN and aDN to identify SMP550 (452689494) as not only the strongest input to aDN, but also to oviDNa. SMP550 is also the only strongly connected upstream neuron matching the morphology of the oviEN neurons[56] as seen in stable split lines 49443 and 65426 (http://splitgal4.janelia.org/cgi-bin/splitgal4.cgi).

oviDNa and SLP410 (450971893) are downstream neurons of aDN which are morphologically similar. Their morphology matches neurons labeled by SS46540 and SS35666 published in Wang et al.[56] as suggested by neuronbridge (https://neuronbridge.janelia.org). Since Wang et al.[56] suggested 2 oviDNa neurons per hemisphere, we propose that they correspond to the neurons named oviDNa and SLP410 in the hemibrain dataset. SMP550/oviEN are also strong inputs to SLP410/oviDNa2, further corroborating this claim.

The newly identified clusters of VNC ascending neurons projecting through the lALT tract, clusters lALT1-5, are based on clustering of morphological features.

Anatomical and connectivity data of neurons obtained from neuPrint has been used to identify PNs and LH associated neurons following published criteria[52,73,74]. In brief: LH associated neurons have dendritic projections inside the LH and receive olfactory PN input. They also have outputs outside the LH. Projection neurons convey information from sensory processing neuropils to the protocerebrum. They are putatively olfactory when they receive inputs in the antennal lobe. Projection neurons of other, potentially thermo-/hygro-, modalities, ascend from the SEZ which is not present in the hemibrain dataset and have been classified according to Marin et al.[52]

Calcium imaging

In vivo calcium imaging of flies expressing *GCaMP6f* was performed at 5.92 frames per second, 256 X 256 pixels resolution, using a Two-Photon microscope (Scientifica) controlled by ScanImage 3.8 software.[75] Fluorescence was excited by a Ti-Sapphire laser (Chameleon Ultra II, Coherent) at ~140 fs pulses, 80 MHz repetition rate, centered on 910 nm. Male and female flies were kept separately from eclosion. All flies except those used as non-retinal controls were aged on food containing 1mM all-trans-retinal. Mated females were generated by mating virgin females with Canton S males 24-48h before the imaging experiment. 3 to 8-day-old adult virgin male, virgin female and mated female flies were anaesthetized on ice and mounted in a custom 3D-printed recording chamber using dental wax. The head capsule was opened using fine forceps and the brain was bathed in carbogenated (95% O_2, 5% CO_2) haemolymph-like solution containing 103mM NaCl, 3mM KCl, 5mM TES, 26mM $NaHCO_3$, 1mm NaH_2PO_4, 1.5mM $CaCl_2$, 4mM $MgCl_2$, 10mM trehalose, 10mM glucose[29]. For the experiments in Figures 3H, 3I, S2E, and S2F, the recording solution additionally contained 50 μM CGP54626 (Sigma Aldrich) and 150-300 μM Picrotoxin (Sigma Aldrich). Optogenetic stimulation was delivered by a red LED (Multicomp OSW-6338, 630 nm) with a stimulation frequency of 40 Hz and individual pulse duration of 10 ms unless stated otherwise. Images were corrected for X and Y movement using image stabilizer in Fiji (http://www.cs.cmu.edu/~kangli/code/Image_Stabilizer.html). All experiments that showed substantial movement in Z were discarded. Regions of interest (ROI) were

selected manually. Subsequent analysis was carried out using custom scripts in MATLAB and python. To obtain average ΔF/F traces, average fluorescence in the ROI was measured against the average baseline fluorescence from 10 s to 200 ms before the onset of the optogenetic stimulation. Experiments were aligned to the optogenetic stimulus. Four optogenetic stimuli were delivered per fly at an inter-stimulus interval of 20 s, and within-fly averages were subsequently averaged across flies. Within-fly averages were used for subsequent statistical analysis and n is reported as the number of flies. Area under the curve (AUC) was calculated as the integral of the ΔF/F traces from the beginning until 1 s after the end of the optogenetic stimulus with a baseline of 1 s before the stimulus.

Courtship assays

Individual virgin males were collected and aged for 5–7 days post-eclosion while virgin females were aged for 3–5 days post-eclosion at 25°C in groups of 3-5 flies. Courtship assays were carried out at 25°C where individual females were introduced into one side of a round courtship chamber with a retractable divider in the middle (20 mm diameter × 2 mm height) with an individual naive male on the other side. The arenas were constructed out of custom cut acrylic (https://southernacrylics.co.uk/) with 20 chambers per plate and an arrangement of 4x5 chambers. Chamber plates were mounted in a custom 3D printed support and were backlight with the FLFL-Si200-IR24 infrared backlight (http://www.falcon-illumination.com/productdetail_FLFL.php). Videos were recorded using the Basler ace A2440-75um camera (cat# 35927) with a 35mm VIS-NIR fixed focal length lens (cat# 67716) and a UV/VIS cut-off filter (cat# 89834) from Edmund Optics (https://www.edmundoptics.co.uk/). The uncompressed AVI videos were recorded at a resolution of 2400x1600 pixels, 8-bit grayscale, 25 frames per second, for 1 hour using LabVIEW software (https://www.ni.com/en-gb/shop/labview.html).

Automated behavior tracking

Uncompressed AVI video files were converted to μFMF video files using '*any2ufmf*' software (http://ctrax.sourceforge.net/any2ufmf.html). The μFMF video files were tracked with Caltech FlyTracker (http://www.vision.caltech.edu/Tools/FlyTracker/)[69] with the following settings: num_chunks = 1, num_cores = 1, max_minutes = 15, save_JAABA = 1. Courtship behaviors were annotated using the Janelia Automatic Animal Behavior Annotator, JAABA (http://jaaba.sourceforge.net/)[70]. Classifiers were trained for the following behaviors:

- '*Approaching*' – focal fly was approaching the other fly (TP = 96.8%, FN = 3.2%, TN = 98.5%, FP = 1.5%, nP = 2715, nN = 16169).
- '*Facing*' – the focal flies head was oriented toward the other fly, while not being on the opposite side of the chamber (TP = 100.0%, FN = 0.0%, TN = 95.2%, FP = 4.8%, nP = 4490, nN = 7937).
- '*Contact*' – the leg, proboscis, or head of the focal fly contacted any part the other fly (TP = 93.9%, FN = 6.1%, TN = 98.1%, FP = 1.9%, nP = 2684, nN = 10789).
- '*Circling*' – the focal fly walked sideways while facing and being close to the other fly (TP = 71.6%, FN = 28.4%, TN = 98.5%, FP = 1.5%, nP = 638, nN = 9746).
- '*Turning*' – the focal fly turned their body to orient toward the other fly while not moving forward (TP = 89.6%, FN = 10.4%, TN = 96.6%, FP = 3.4%, nP = 414, nN = 1540).
- '*Wing extension*' – the focal fly extended a wing beyond their body (TP = 98.3%, FN = 1.7%, TN = 98.6%, FP = 1.4%, nP = 1575, nN = 10926).

(TP – true positive, FN – false negative, TN – true negative, FN – false negative, nP – number of annotated positive frames, nN – number of annotated negative frames).

Classifiers were applied to the tracking data using the '*JAABADetect*' function.

Female fertility

% Fertility is the proportion of females that produce viable progeny. Females tested for fertility were collected at eclosion, stored in groups of 3-5 and aged for 5 days. They were then introduced individually into food vials containing three wild-type virgin males aged 5-7 days. All vials were scored for presence of larval progeny after 10 days. Vials containing a dead female were discounted.

Female egg-laying preference assay

Pheromone extraction

Pheromones were extracted from the cuticle of five-day old male *Canton-S* flies with n-hexane (Fisher Scientific). The flies were collected under CO_2 anesthesia on the day of eclosion and left to mature in groups of 10-15 in 25 mm x 95 mm rearing vials. Prior to the extraction the flies were anesthetized on ice and transferred into 2 mL glass screw cap vials. 12 μL of n-hexane was added to the vial per fly and the vial was vortexed for 3 min after which the supernatant was carefully transferred into a clean vial. Due to minor evaporation and absorption of the hexane by the fly bodies, the remaining supernatant contained approximately 10 μL of hexane extract per fly, which was the dose used to represent a single fly in the behavioral experiments.

Oviposition experiments

The flies were collected on the day of eclosion and left to mature for five days before mating them in groups of 10 males with 10 females in rearing vials for approximately four hours. All genotypes were mated to *Canton-S* males. After mating all females

were kept on a 3% bacteriological agar substrate overnight (Fisher Scientific) with a small amount of yeast extract paste (Fisher Scientific), to provide food and moisture to enhance oviposition. On the day of the experiment the females were individually transferred into oviposition assays (57 × 38 × 17 mm) containing a 3% agar middle zone unsuitable for oviposition and two oviposition zones of 0.75% agar containing 100 mM of sucrose (Sigma-Aldrich) on either end of the assay. The oviposition zones enclosed a 3 mm filter disc (Chromatography paper, Whatman) containing either the odor treatment or the solvent as control. To prevent odor saturation, the assays were covered with Parafilm (Sigma-Aldrich) that was punctured several times with a fine needle above the oviposition zones. After 24 hours the number of eggs laid per oviposition zone was counted under a stereomicroscope, and the oviposition indices were calculated as follows: (Eggs side A - Eggs side B) / (Eggs side A + Eggs side B).

QUANTIFICATION AND STATISTICAL ANALYSIS

Analysis of courtship data

Analysis of courtship data was conducted in R (https://www.r-project.org/). Unless otherwise stated, all functions are from either '*base*' R or the '*stats*' package. Data wrangling and plotting were conducted using the '*tidyverse*' set of packages[76], including '*readr*', '*dplyr*', '*ggplot2*'. Time to copulation was assessed by manual inspection of the videos. Tracking data was sub-sectioned to remove frames for which the flies were copulating. Courtship initiation was defined as exhibiting any combination of the courtship (JAABA) behaviors (excluding facing) for a total of 3 s over a 6 s window. For all subsequent analyses all frames before courtship initiation were removed. Courtship index is the percentage of time males exhibit any courtship (JAABA) behavior. Similarly, individual courtship (JAABA) behavior indices are defined as the percent of time exhibiting that behavior. Cumulative copulation was analyzed using the Kaplan-Meier method with the '*survfit*' function form the '*survival*' package[77] and plotted with the '*ggsurvplot*' function from the '*survminer*' package[78]. Statistical significances were assessed with a log-rank test using the '*survdiff*' function from '*survival*', and the '*pairwise_survdiff*' function from '*survminer*' for pairwise differences.

Probability density function plots were generated by taking the mean of individual density curves (generated with the '*density*' function with default parameters) per genotype of the '*facing_angle*' and '*dist_to_other*' features generated by the FlyTracker software. Facing angle ('*facing_angle*') is defined as the angle between the line bisecting the focal fly in the direction in which it is facing (see inset in Figure 4E), and the line between the centroids of the two flies. Distance to the other fly ('*dist_to_other*') is the distance between the centroids of the two flies. Statistical differences were assessed using the 2-sided Kolmogorov–Smirnov test[79] using the '*ks.test*' function with default parameters.

Bilateral wing extension was defined as both wings being extended at an angle greater than 15° ('*min_wing_ang*' > 0.26 radians). This value was chosen as 97.5% of individuals had a mean lesser wing angle of less than 15° (Figure S4K). Bilateral bout length and the number of bouts were determined using the '*rle*' package. Unilateral wing extension was defined as one wing extended more than 35° ('*max_wing_ang*' > 0.61 radians) while the other was extended less than 15° ('*min_wing_ang*' < 0.26 radians).

Ipsilateral wing extension was defined as having a left wing angle of greater than 35° ('*wing_l_ang*' > 0.61 radians) while the female was located to the left of the male's body axis, or right wing angle of greater than 35° ('*wing_r_ang*' > 0.61 radians) while the female was to the right of the male's body axis. Vice versa for contralateral wing extension. Wing choice index was defined as the amount of time the ipsilateral wing was extended, minus the amount of time the contralateral wing was extended, all divided by the sum of time either wing was extended[25].

Statistical significance for Figures 4A, 4B, 4G–4K, and S4A–S4J were assessed with one-way ANOVAs conducted using the '*aov*' function, followed by post hoc 2-sided t tests ('*t.test*' function). All statistical values (for ANOVAs, t tests, and other mentioned statistical tests) were adjusted for multiple testing (for each behavior component) and multiple comparison (for each genotype) using the Holm-Bonferroni method ('*p.adjust*' function with *method = "holm"*).

Statistical analysis of oviposition

The oviposition indices were first analyzed for a preference to the pheromone extract over the control treatment using a two-tailed Wilcoxon Signed Rank tests with the null hypothesis assuming no preference to either of the treatments (mu = 0). Afterward, the preferences to the pheromone extracts were compared across genotypes using a generalized linear model (GLM) with a quasibinomial error distribution. In order to use the quasibinimioal error distribution the data was analyzed using a 'cbind' including the number of eggs laid on the side with the pheromone extract and the number of eggs laid on the side with the control treatment, generating values between 0 (preference for control treatment) and 1 (preference for odour treatment). Afterward, a GLM with a negative binomial error distribution was run to test for differences among genotypes for the total number of eggs laid. The model assumptions were checked by estimation of overdispersion and inspections of model residuals. All analyses were carried out in R (v. 3.6.1). The GLMs were performed using lme4 and car[80,81] for model comparison based on $\chi 2$ likelihood ratios[82] and the data was visualized using ggplot2.[76]

Statistical analysis of calcium imaging data

$\Delta F/F$ are reported as mean and SE. Statistical significance was tested by the Kruskal-Wallis test, followed by pairwise comparisons with the Mann-Whitney-U test using the scipy and statsmodels packages in python and significance levels were adjusted for multiple comparisons using the Holm-Bonferroni method. Significance levels are reported as follows: $p \geq 0.01$: ns, $p < 0.01$: $\cdot$, $p < 0.05$: *, $p < 0.01$: **, $p < 0.001$: ***.

Current Biology, Volume *31*

Supplemental Information

A sex-specific switch between visual and olfactory inputs underlies adaptive sex differences in behavior

Tetsuya Nojima, Annika Rings, Aaron M. Allen, Nils Otto, Thomas A. Verschut, Jean-Christophe Billeter, Megan C. Neville, and Stephen F. Goodwin

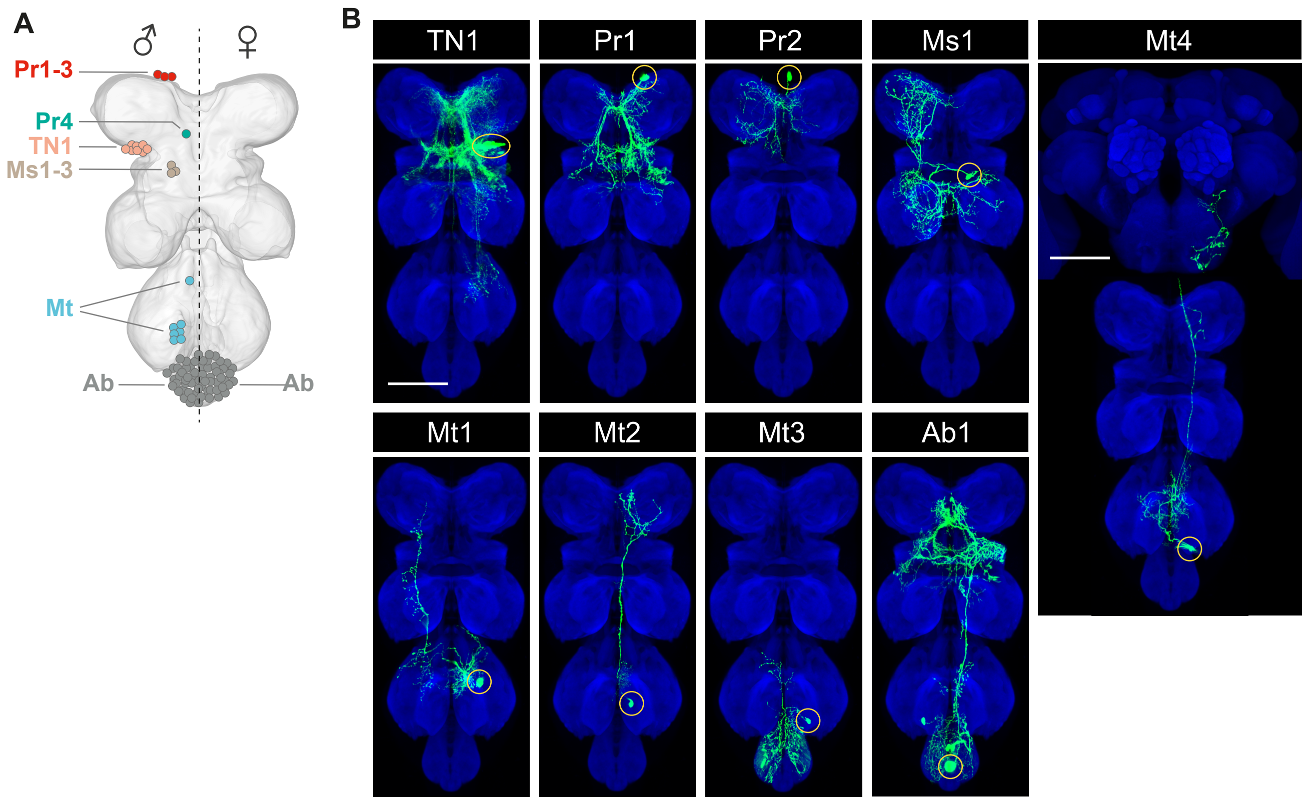
A
♂
♀
Pr1-3
Pr4
TN1
Ms1-3
Mt
Ab
Ab
B
TN1
Pr1
Pr2
Ms1
Mt4
Mt1
Mt2
Mt3
Ab1

Figure S1: Comprehensive single cluster-level mapping of *dsx*-expressing neurons in the male VNC. Related to Figure 1.

(**A**) A schematic drawing representing *dsx* expression pattern in the VNC. Male neurons are shown in the thoracic neuromeres on the left, the absence of female neurons is shown on the right. Neuronal differences between the sexes are not distinguished in the abdominal neuromere. (**B**) Male single neuroblast clones generated by dsx^{Gal4}-driven MARCM. Cell body positions are indicated by yellow circles. Scale bars, 50 µm.

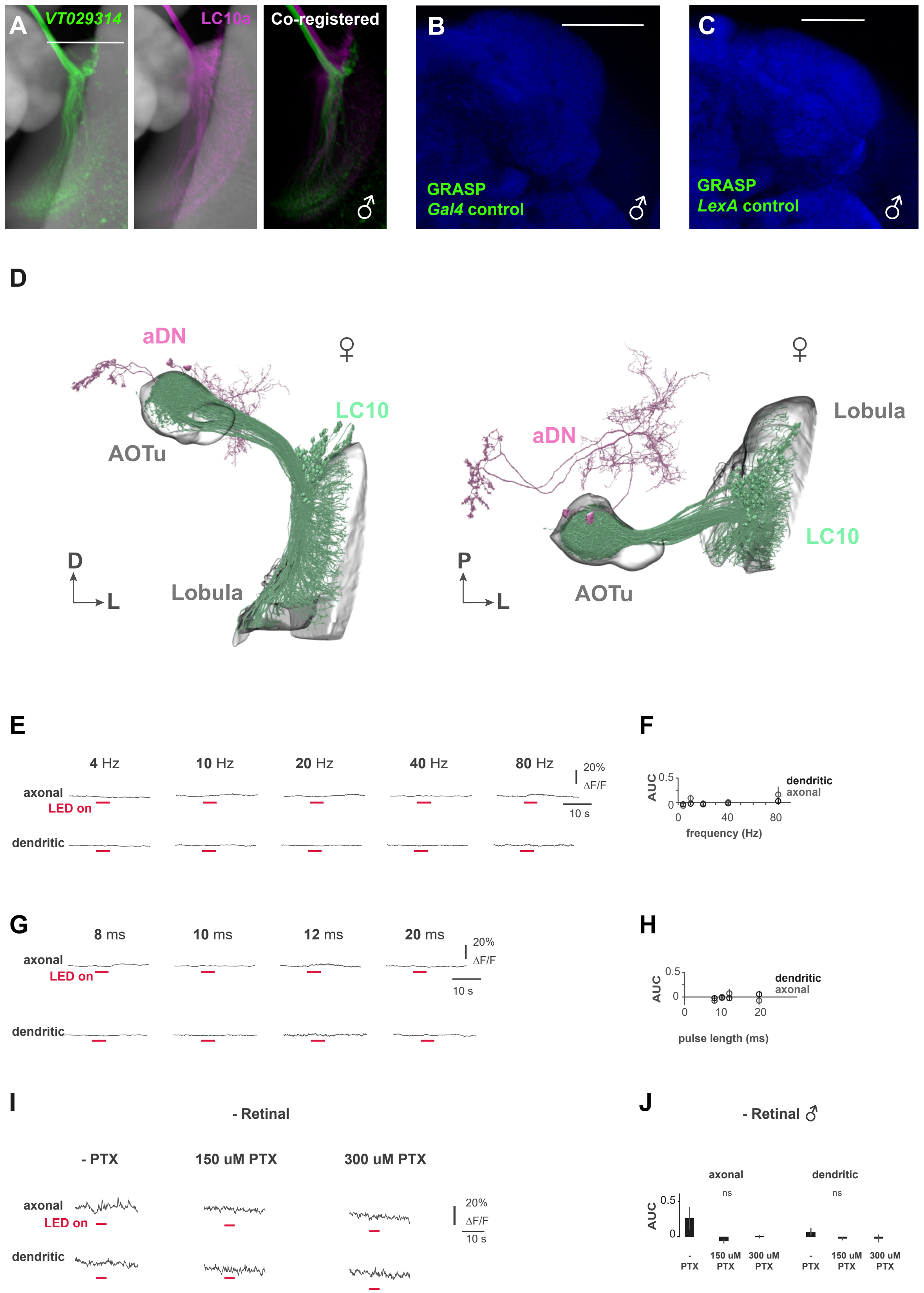
A
VT029314
LC10a
Co-registered
B
GRASP
Gal4 control
C
GRASP
LexA control
D
aDN
LC10
AOTu
Lobula
D
L
P
L
E
4 Hz
10 Hz
20 Hz
40 Hz
80 Hz
20%
ΔF/F
10 s
axonal
LED on
dendritic
F
AUC
dendritic
axonal
frequency (Hz)
G
8 ms
10 ms
12 ms
20 ms
H
pulse length (ms)
I
- Retinal
- PTX
150 uM PTX
300 uM PTX
J
- Retinal
ns
- PTX
150 uM PTX
300 uM PTX

Figure S2: Connectivity between LC10a and aDN is male-specific. Related to Figure 3.

(**A**) LC10 neurons labeled by *VT029314-LexA* (green) and *OL0019B* split-*Gal4* (magenta) are co-registered (right). Scale bar, 50 μm. (**B and C**) Genetic controls for the GRASP experiment either without *LexA* (**B**) or *Gal4* (**C**) driver. Scale bars, 30 μm. (**D**) Positions of LC10 (green) axon terminals and aDN (magenta) dendritic fields in females are analyzed using the EM-level connectomics database neuPrint. Anterior (left) and dorsal (right) views are shown. Two neuropil regions, lobula and AOTu, which are innervated by LC10 dendrites and axons, respectively, are indicated in gray. (**E**) Mean (dark gray line) and standard error (SE; light gray shaded area) of ΔF/F in aDN axonal (top) and dendritic (bottom) compartments in response to a 5 s (40 Hz, 10 ms pulses) optogenetic stimulation of LC10a neurons (pink bar) in control males not fed on all-*trans*-retinal; Left: without picrotoxin (- PTX) and CGP54626 (n = 13 male); Middle: 150 μM picrotoxin and 50 μM CGP54626 (n = 8 male flies); Right: 300 μM picrotoxin and 50 μM CGP54626 (n = 10 and 8 male flies in axonal and dendritic compartments, respectively); (**F**) Mean and SE of the area under the ΔF/F curve (AUC) from beginning until 1s after the end of the stimulus for experiments in (**E**). (**G**) Mean and SE of ΔF/F traces in aDN axonal (top traces) and dendritic (bottom traces) compartments in response to different frequencies (4-80 Hz, 10 ms pulses) of a 5 s optogenetic activation of LC10a neurons in 9-28 males for each frequency. (**H**) AUC vs. frequency for data in (**G**) in axonal (light gray circles) and dendritic (dark gray circles) compartments. (**I**) Mean ΔF/F traces in aDN axonal (top traces) and dendritic (bottom traces) compartments in response to different pulse lengths (8-20 ms, 40 Hz pulses) of a 5 s optogenetic activation of LC10a neurons in 5-28 males for each pulse length. (**J**) AUC vs. pulse length for data in (**I**). ns – *p.adj*>0.05 by Mann-Whitney-U test adjusted for multiple comparisons with the Holm method.

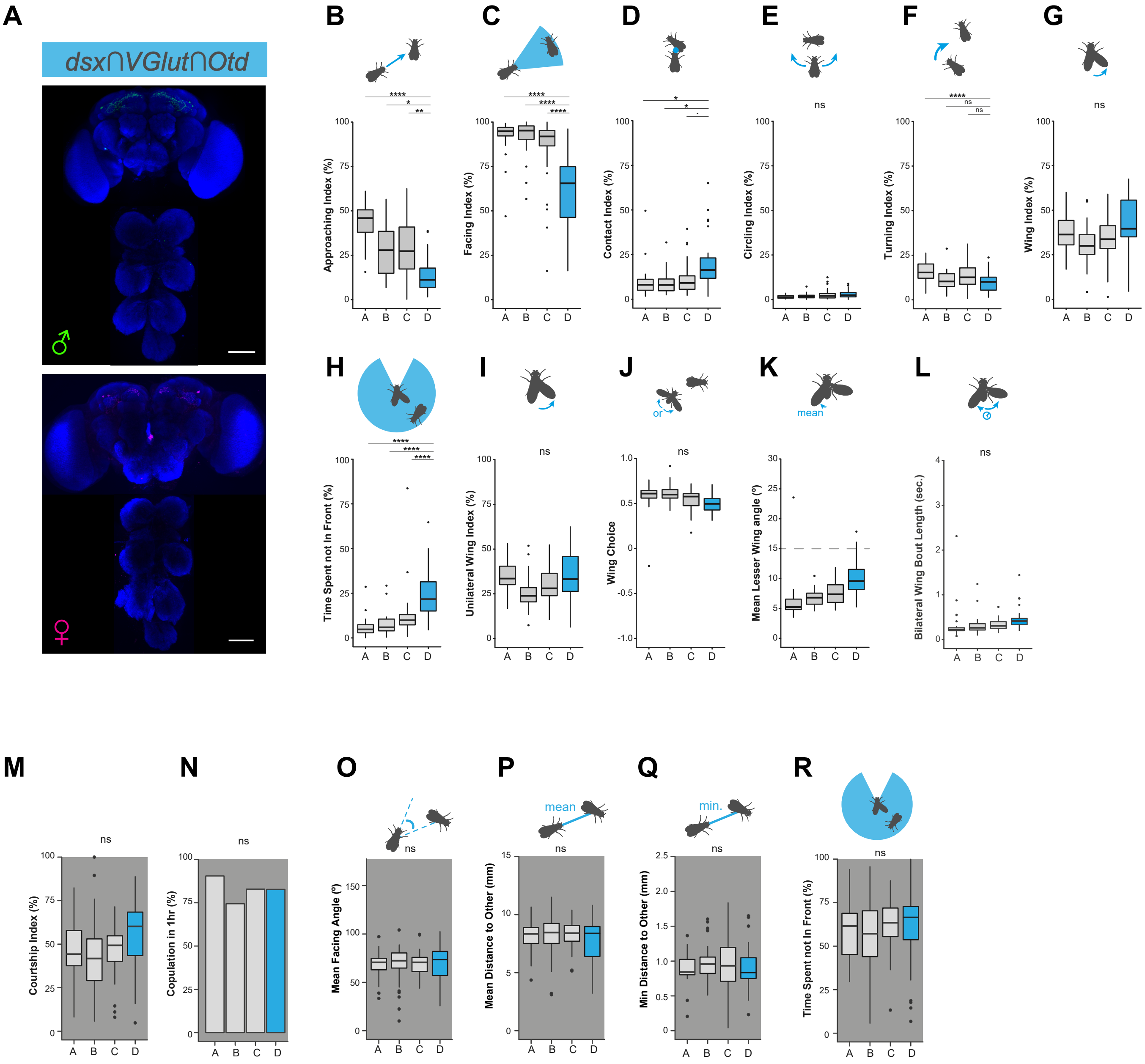
A
dsx∩VGlut∩Otd
B
Approaching Index (%)
C
Facing Index (%)
D
Contact Index (%)
E
Circling Index (%)
F
Turning Index (%)
G
Wing Index (%)
H
Time Spent not In Front (%)
I
Unilateral Wing Index (%)
J
Wing Choice
or
K
Mean Lesser Wing angle (°)
mean
L
Bilateral Wing Bout Length (sec.)
M
Courtship Index (%)
N
Copulation in 1hr (%)
O
Mean Facing Angle (°)
P
Mean Distance to Other (mm)
mean
Q
Min Distance to Other (mm)
min.
R
Time Spent not In Front (%)
ns
A B C D

Figure S3: Silencing male aDN alters visually guided courtship behavior. Related to Figure 4.

(**A**) Spatially restricted expression in aDN cluster. *dsx∩VGlut∩Otd* expression patterns shown using *dsx*$^{Gal4.DBD}$*/VGlut*$^{dVP16.AD}$*/Otd-FLP > UAS>stop>mCD8::GFP* in the male (green) and female (magenta) brains and VNCs. Scale bar, 50 µm. (**B-L**) Schematic representations of the behaviors are inset. (**B-F**) Individual indices of each component of courtship behavior for which a JAABA classifier was trained. The reported *p.adj* are *p* values adjusted (Holm method) for comparison of multiple behaviors. (**B**) Approaching index. ($F_{(3,114)}$ = 26.2, *p.adj <0.0001*). (**C**) Facing index ($F_{(3,114)}$ = 23.7, *p.adj <0.0001*). (**D**) Contact index ($F_{(3,114)}$ = 6.91, *p.adj* = 0.008). (**E**) Circling index ($F_{(3,114)}$ = 4.11, *p.adj* = 0.16). (**F**) Turning index ($F_{(3,114)}$ = 6.61, *p.adj* = 0.011). (**G**) Wing extension index. ($F_{(3,114)}$ = 3.87, *p* = 0.012, *p.adj* = 0.19). (**H**) Percent of time when the female was not in front of the male while the males wing was extended. ($F_{(3,114)}$ = 28.6, *p.adj <0.0001*). (**I**) Unilateral wing index. ($F_{(3,114)}$ = 3.40, *p.adj* = 0.32). (**J**) Wing choice index. ($F_{(3,114)}$ = 4.41, *p.adj* = 0.11). (**K**) Bilateral wing bout length. ($F_{(3,114)}$ = 0.619, *p.adj* = 1.0). (**L**) Mean lesser wing angle. (**M**) Courtship Index (%) in the dark. ($F_{(1,123)}$ = 3.143, *p.adj* = 0.17). (**N**) Copulation (%) over a 60-minute time period in the dark. (Fisher's exact test, *p.adj* = 1.00). (**O**) Mean facing angle in the dark. ($F_{(1,123)}$ = 0.173, *p.adj* = 1.00). (**P**) Mean distance to other in the dark. ($F_{(1,123)}$ = 0.84, *p.adj* = 1.00). (**Q**) Minimum distance to other in the dark. ($F_{(1,123)}$ = 0.453, *p.adj* = 1.00). (**R**) Percentage of time when the female was not in front of the male while the male's wing was extended in the dark. ($F_{(1,123)}$ = 0.536, *p.adj* = 1.00) Full genotypes: A=*Otd-FLP/+; dsx*$^{Gal4.DBD}$*/+* (n_{light}=31, n_{dark}=21), B=*VGlut*$^{dVP16.AD}$*, UAS>stop>TNT/+* (n_{light} =20, n_{dark}=43), C=*VGlut*$^{dVP16.AD}$*, UAS>stop>TNT/+; dsx*$^{Gal4.DBD}$*/+* (n_{light}=34, n_{dark}=35), D=*VGlut*$^{dVP16.AD}$*, UAS>stop>TNT/Otd-FLP; dsx*$^{Gal4.DBD}$*/+* (n_{light} =33, n_{dark}=29). ˙*p.adj*<0.1, **p.adj*<0.05, ***p.adj*<0.01, ****p.adj*<0.001, *****p.adj*<0.0001 by t-test (**B-R**) and adjusted with the Holm method. ns – *p.adj*>0.1.

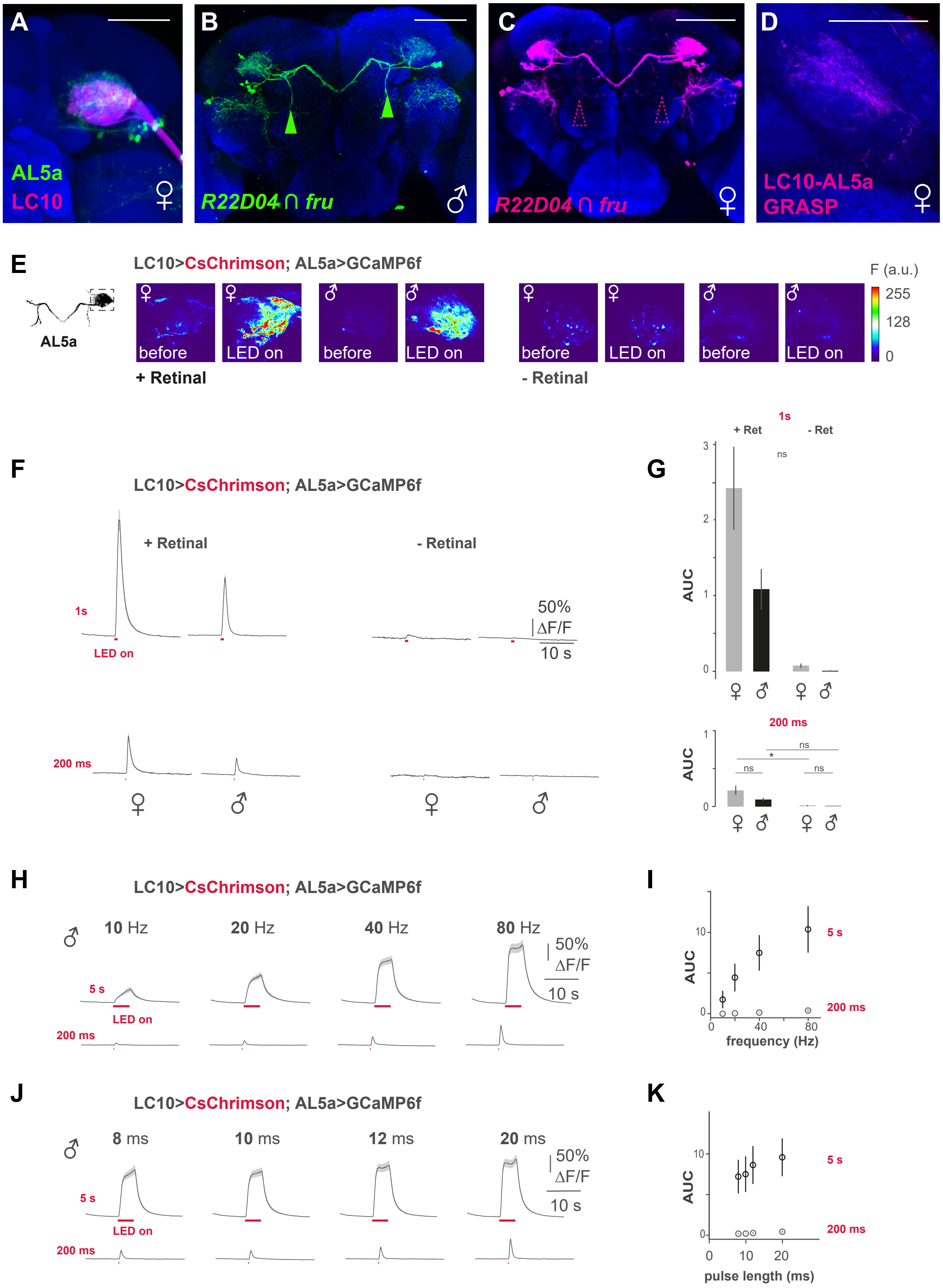
A
AL5a
LC10
♀
B
R22D04 ∩ fru
♂
C
R22D04 ∩ fru
♀
D
LC10-AL5a
GRASP
♀
E
AL5a
LC10>CsChrimson; AL5a>GCaMP6f
F (a.u.)
255
128
0
before
LED on
+ Retinal
- Retinal
F
LC10>CsChrimson; AL5a>GCaMP6f
+ Retinal
- Retinal
1s
LED on
200 ms
50%
ΔF/F
10 s
G
1s
+ Ret
- Ret
ns
AUC
200 ms
ns
*
H
LC10>CsChrimson; AL5a>GCaMP6f
10 Hz
20 Hz
40 Hz
80 Hz
5 s
LED on
200 ms
50%
ΔF/F
10 s
I
AUC
5 s
200 ms
frequency (Hz)
J
LC10>CsChrimson; AL5a>GCaMP6f
8 ms
10 ms
12 ms
20 ms
5 s
LED on
200 ms
50%
ΔF/F
10 s
K
AUC
5 s
200 ms
pulse length (ms)

Figure S4: *fru*-expressing AL5a is an additional downstream cluster of LC10a. Related to Figure 5.

(**A**) The female LC10a (magenta) and AL5a (green) co-registered on to the template brain. Scale bar, 30 μm. (**B,C**) Expression patterns of *GMR22D04-Gal4∩fru*^{FLP} in the male (green) and female (magenta) brains. Solid filled (**B**) and dashed open (**C**) arrowheads indicate the presence and absence, respectively, of male-specific longitudinal neurite branches. Scale bars, 50 μm. (**D**) GRASP signals between LC10a and AL5a in the female (magenta). Scale bar, 30 μm. (**E**) Left: Maximum projection of a confocal stack of mCD8::GFP-labeled AL5a in a male. Recording region in the AOTu for calcium imaging is indicated by a gray box. Right: Example fluorescence recordings from the input sites of AL5a neurons in the AOTu expressing GCaMP6f in males and females before (left panel) and during (right panel) a 5 s (40 Hz, 10 ms) optogenetic stimulation of LC10a neurons expressing CsChrimson. Left (+ Retinal) Right (-Retinal). (**F**) Mean (dark gray) and SE (light gray shaded area) of ΔF/F traces in AL5a neuron input sites in response to a 1 s (top) and 200 ms (bottom) optogenetic activation (pink bar, 40 Hz, 10 ms pulses) of LC10a neurons; Averages of 4 pulses each in retinal fed (+Retinal, left) female (left, n=7) and male (right, n=11) flies and in non-retinal control (-Retinal, right) female (left, n=8) and male (right, n=7) flies. (**G**) Mean and SE of AUC for experiments in (**F**); Top: 1 s optogenetic stimulation; Bottom: 200 ms optogenetic stimulation. (**H**) Mean ΔF/F traces in AL5a neurons in response to different frequencies (10 - 80 Hz, 10 ms pulses) of a 5 s (top) and 200 ms (bottom) optogenetic activation of LC10a in n = 11-13 males for each frequency. (**I**) Mean and SE of AUC for experiments in (**H**) for 5 s (dark gray circles) and 200 ms (light gray circles) optogenetic stimulation of LC10a. (**J**) Mean ΔF/F traces in AL5a neurons in response to different pulse lengths (8 - 20 ms, 40 Hz pulses) of a 5 s (top) and 200 ms (bottom) optogenetic activation of LC10a in 10 -13 males for each pulse length. (**K**) Mean and SE of AUC for experiments in (J) for 5 s (dark gray circles) and 200 ms (light gray circles) optogenetic stimulation of LC10a. ns – *p.adj*>0.05, **p.adj*<0.05, ***p.adj*<0.01, ****p.adj*<0.001, *****p.adj*<0.0001 by Mann-Whitney-U test adjusted for multiple comparisons with the Holm method.

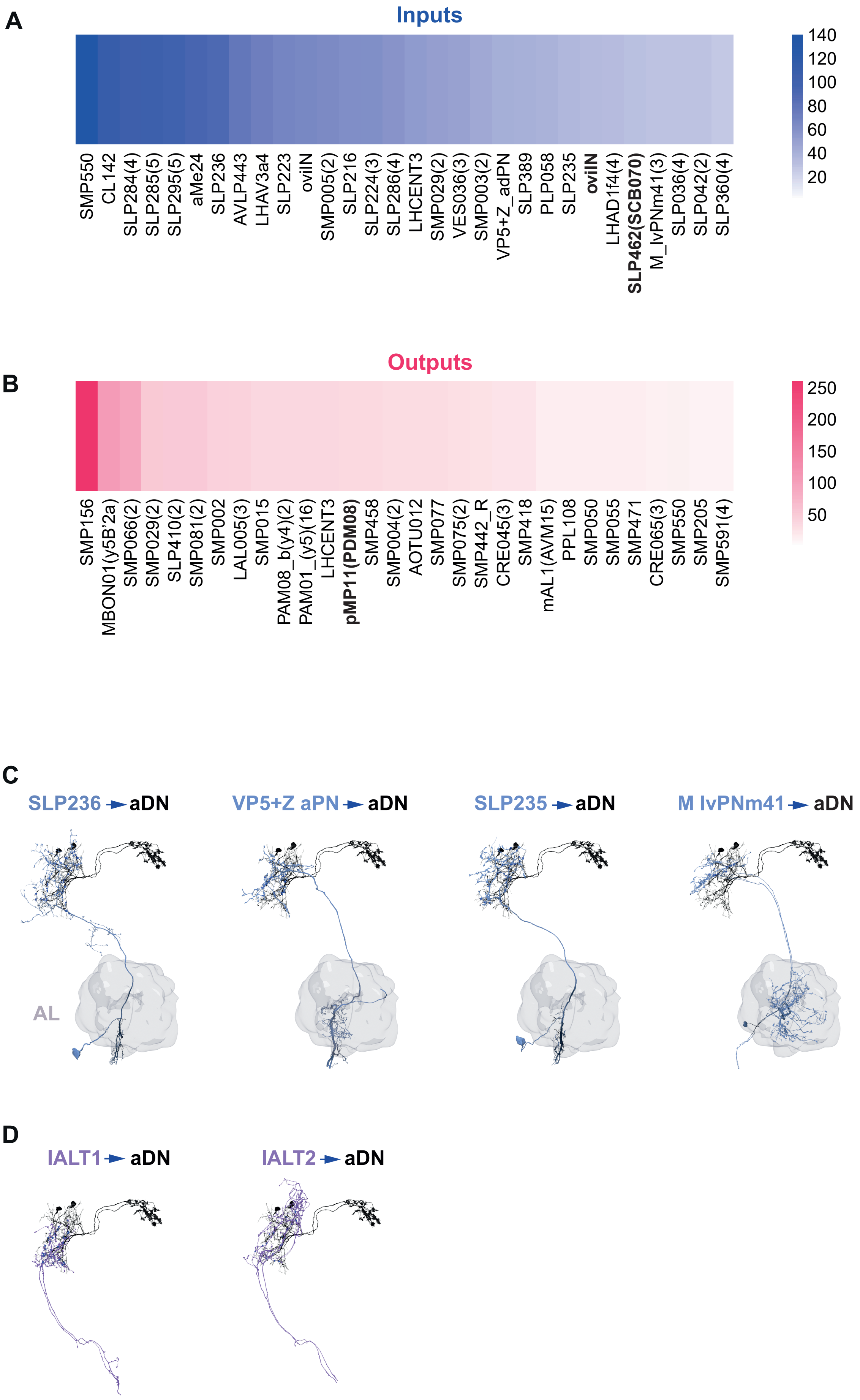

A
Inputs
140
120
100
80
60
40
20
SMP550
CL142
SLP284(4)
SLP285(5)
SLP295(5)
aMe24
SLP236
AVLP443
LHAV3a4
SLP223
oviIN
SMP005(2)
SLP216
SLP224(3)
SLP286(4)
LHCENT3
SMP029(2)
VES036(3)
SMP003(2)
VP5+Z_adPN
SLP389
PLP058
SLP235
oviIN
LHAD1f4(4)
SLP462(SCB070)
M_lvPNm41(3)
SLP036(4)
SLP042(2)
SLP360(4)
B
Outputs
250
200
150
100
50
SMP156
MBON01(y5B'2a)
SMP066(2)
SMP029(2)
SLP410(2)
SMP081(2)
SMP002
LAL005(3)
SMP015
PAM08_b(y4)(2)
PAM01_(y5)(16)
LHCENT3
pMP11(PDM08)
SMP458
SMP004(2)
AOTU012
SMP077
SMP075(2)
SMP442_R
CRE045(3)
SMP418
mAL1(AVM15)
PPL108
SMP050
SMP055
SMP471
CRE065(3)
SMP550
SMP205
SMP591(4)
C
SLP236 aDN
VP5+Z aPN aDN
SLP235 aDN
M lvPNm41 aDN
AL
D
lALT1 aDN
lALT2 aDN

Figure S5: Female aDN connectivity. Related to Figure 6.

(**A and B**) Heatmaps representing number of synaptic connections to both aDN combined input (**A**) and output (**B**) by neuronal types. Neurons labeled in bold are contralateral, all others are ipsilateral. Color scales represent the number of synapses. (**C and D**) 3D reconstructions of volumetric EM data of aDN (2 neurons, grey) and projection neurons in the right-brain hemisphere. (**C**) The multiglomerular multimodal projection neurons VP5+Z aPN (bodyid: 5813063239) and M lvPNm41 (bodyids: 730252777, 1765415970, 1795410644) are shown as well as the uncharacterized non-olfactory projection neurons SLP236 (bodyid: 420274150) and SLP235 (5813056890). Inputs into the aDN are shown in blue. The antennal lobe (AL) is shown. (**D**) The lALT-tract neurons classes lALT1 (bodyid: 888500672) and lALT2 (bodyid: 5901195362) are shown.

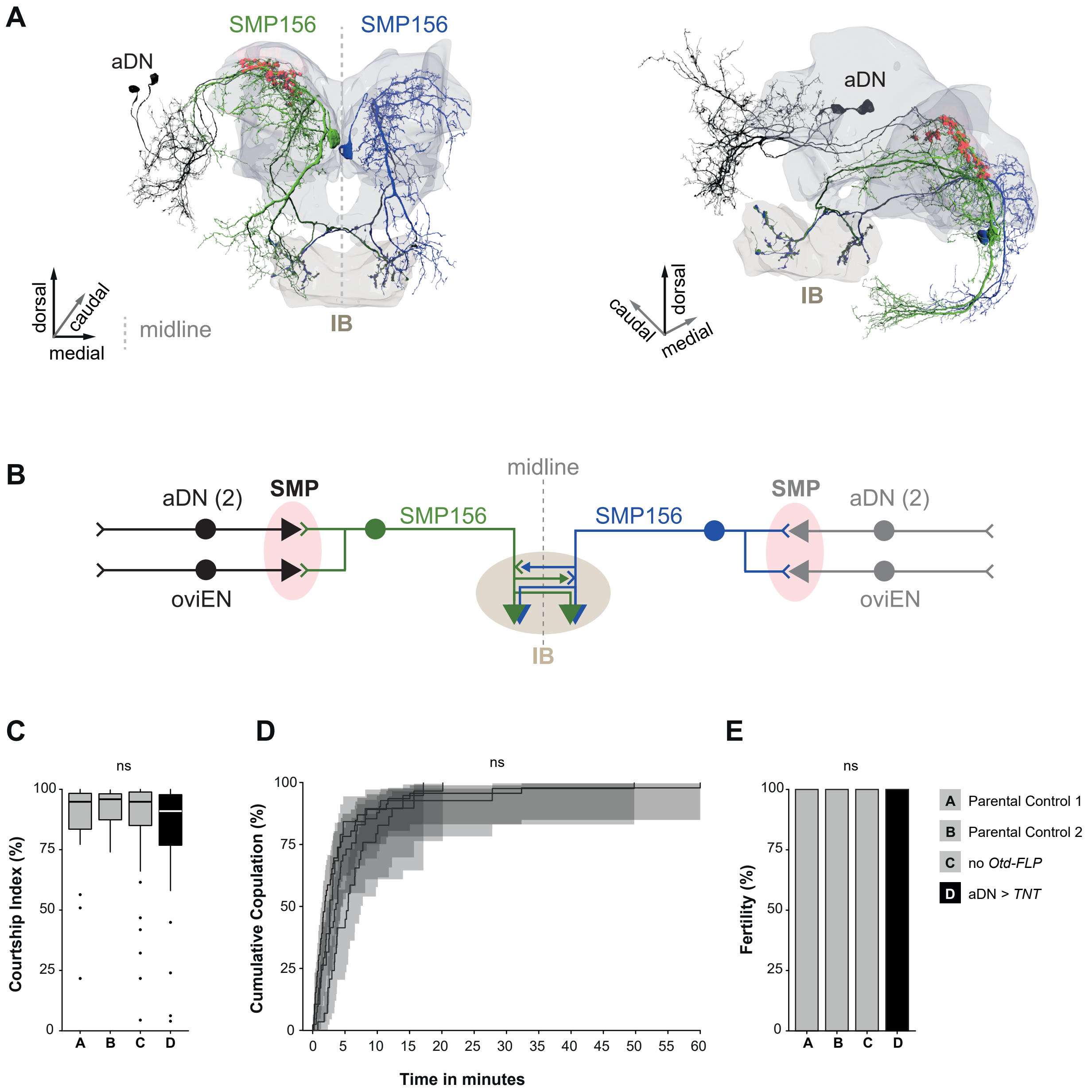
A
SMP156
SMP156
aDN
dorsal
caudal
medial
midline
IB
aDN
IB
caudal
dorsal
medial
B
midline
aDN (2)
SMP
SMP156
SMP156
SMP
aDN (2)
oviEN
oviEN
IB
C
ns
Courtship Index (%)
D
ns
Cumulative Copulation (%)
Time in minutes
E
ns
Fertility (%)
A Parental Control 1
B Parental Control 2
C no Otd-FLP
D aDN > TNT

Figure S6: Female aDNs are not involved in virgin female behavior. Related to Figure 7.

(**A**) 3D reconstructions of volumetric EM data from aDN (2 neurons, grey) and the contra- and ipsilateral downstream neurons SMP156 (bodyids: 5813022337, 673776769). The inferior bridge (IB) and the SMP for both hemispheres are shown as well. Synapses from aDN to SMP156 are shown in red. (**B**) Circuit diagram shows aDN and oviEN inputs to SMP156 that projects to the IB. (**C-E**) Silenced female aDN behaviors (ns – *p.adj*>0.05). (**C**) Control male courtship indices toward female. ($F_{(3,131)} = 1.1$, $p = 0.49$, $p.adj = 1$). (**D**) Cumulative proportional copulation (% ± 95% confidence intervals) over a 60-minute time period. ($\chi^2_{(3)} = 5.5$, $p = 0.14$, $p.adj = 1$). (**E**) Individual female fertility over a 1-week period. (Fisher's Exact Test, $p = 1$).

	**Male**	**Female**
	Mean ± SEM (n)	Mean ± SEM (n)
**pC1**	48 ± 3.1 (10)	6 ± 0.3 (8)
**pC2m**	31 (1)	NA
**pC2l**	44 ± 2.4 (10)	13 ± 0.5 (7)
**pCd-1**	15 (2)	8 ± 1.1 (2)
**pCd-2**	3 (10)	3 (10)
**pMN1**	NA	NA
**pMN2**	NA	1 (10)
**pMN3**	1 (10)	NA
**pLN**	1 (10)	NA
**aDN**	2 (10)	2 (10)
**SN**	1 (10)	NA
**TN1**	24 ± 0.8 (6)	NA
**Pr1**	1 (3)	NA
**Pr2**	1 (1)	NA
**Ms1**	1 (1)	NA
**Mt1**	1 (1)	NA
**Mt2**	1 (1)	NA
**Mt3**	1 (1)	NA
**Mt4**	1 (1)	NA
**Ab1**	1 (1)	NA

Table S1. Numbers of cells in *dsx*$^+$ single clusters. Related to Figure 1.

Figure	Full genotype
1C-1E, S1B	y, w, hs-flp(22); FRTG13, UAS-mCD8::GFP/FRTG13, tubP-Gal80; dsx^{Gal4}/UAS-mCD8::GFP
1C, 2A-2C, 2E-2G, 3B	10xUAS-IVS-mCD8::GFP/VGlut$^{dVP16.AD}$; dsx$^{Gal4.DBD}$/+
1D	10xUAS-IVS-mCD8::GFP/+; dsx$^{Gal4.DBD}$/Trh-p65.AD
2I-2K	UAS-DenMark/VGlut$^{dVP16.AD}$; dsx$^{Gal4.DBD}$/+
2L-2N	UAS-Syb::GFP/VGlut$^{dVP16.AD}$; dsx$^{Gal4.DBD}$/+
3A-3B, 5B-5C, S2A, S4A	R35D04-p65.AD/10xUAS-IVS-mCD8::GFP; R22D06-Gal4.DBD/+
3D-3G	VGlut$^{dVP16.AD}$/VT029314-LexA; dsx$^{Gal4.DBD}$/UAS-CD4::spGFP1-10, lexAop-CD4::spGFP11
3H-3I, S2E-S2J	lexAop-CsChrimson::tdTomato, UAS-GCaMP6f/+; VGlut$^{dVP16.AD}$/VT029314-LexA; dsx$^{Gal4.DBD}$/+
4, 7E-7G, S3B-S3R, S6C-S6E	Otd-FLP/+; dsx$^{Gal4.DBD}$/+
4, 7E-7G, S3B-S3R, S6C-S6E	VGlut$^{dVP16.AD}$, UAS>stop>TNT/+
4, 7E-7G, S3B-S3R, S6C-S6E	VGlut$^{dVP16.AD}$, UAS>stop>TNT/+; dsx$^{Gal4.DBD}$/+
4, 7E-7G, S3B-S3R, S6C-S6E	VGlut$^{dVP16.AD}$, UAS>stop>TNT/Otd-FLP; dsx$^{Gal4.DBD}$/+
5A-5C	y, hs-flp; FRTG13, UAS-mCD8::GFP/FRTG13, tubP-Gal80; fru^{NP21}/UAS-mCD8::GFP
5D-5E, S4A	UAS-DenMark/+; GMR22D04-Gal4/+
5F, S4D	VT029314-LexA/+; GMR22D04-Gal4/UAS-CD4::spGFP1-10, lexAop-CD4::spGFP11
5G-5H, S4E-S4K	lexAop-CsChrimson::tdTomato, UAS-GCaMP6f/+;VT029314-LexA/+; GMR22D04-Gal4/+
6F-6G	lexAop-CsChrimson::tdTomato, UAS-GCaMP6f/+; VGlut$^{dVP16.AD}$/GH146-LexA; dsx$^{Gal4.DBD}$/+
6H-6I	lexAop-CsChrimson::tdTomato, UAS-GCaMP6f/+; VGlut$^{dVP16.AD}$/+; dsx$^{Gal4.DBD}$/Orco-LexA::VP16
S2A	VT029314-LexA/13xlexAop2-IVS-myr::GFP
S2B	VGlut$^{dVP16.AD}$/+; dsx$^{Gal4.DBD}$/UAS-CD4-spGFP1-10, lexAop-CD4-spGFP11
S2C	VT029314-LexA/+; UAS-CD4-spGFP1-10, lexAop-CD4-spGFP11/+
S3A	VGlut$^{dVP16.AD}$/Otd-FLP; dsx$^{Gal4.DBD}$/UAS>stop>mCD8::GFP
S4B-S4C	UAS>stop>mCD8::GFP/+; GMR22D04-Gal4/fru^{FLP}

Table S3. Full genotype list. Related to Figures 1-7 and Figures S1-S4, S6.
